# FGF19-mediated ELF4 overexpression promotes colorectal cancer metastasis through transactivating FGFR4 and SRC

**DOI:** 10.7150/thno.82269

**Published:** 2023-02-22

**Authors:** Xilang Chen, Jie Chen, Weibo Feng, Wenjie Huang, Guodong Wang, Mengyu Sun, Xiangyuan Luo, Yijun Wang, Yongzhan Nie, Daiming Fan, Kaichun Wu, Limin Xia

**Affiliations:** 1 State Key Laboratory of Cancer Biology, National Clinical Research Center for Digestive Diseases and Xijing Hospital of Digestive Diseases, Fourth Military Medical University, Xi'an 710032, Shaanxi Province, China; 2 Department of Gastroenterology, Institute of Liver and Gastrointestinal Diseases, Hubei Key Laboratory of Hepato-Pancreato-Biliary Diseases, Tongji Hospital of Tongji Medical College, Huazhong University of Science and Technology, Wuhan 430030, Hubei Province, China; 3 Hepatic Surgery Center, Tongji Hospital, Tongji Medical College, Huazhong University of Science and Technology; Clinical Medicine Research Center for Hepatic Surgery of Hubei Province; Key Laboratory of Organ Transplantation, Ministry of Education and Ministry of Public Health, Wuhan, Hubei, 430030, China

**Keywords:** E74-like factor 4, Colorectal cancer, Metastasis, Fibroblast growth factor receptor 4, BLU-554

## Abstract

**Background:** Metastasis accounts for the high lethality of colorectal cancer (CRC) patients. Unfortunately, the molecular mechanism manipulating metastasis in CRC is still elusive. Here, we investigated the function of E74-like factor 4 (ELF4), an ETS family member, in facilitating CRC progression.

**Methods:** The expression of ELF4 in human CRC samples and CRC cell lines was determined by quantitative real-time PCR, immunohistochemistry and immunoblotting. The migratory and invasive phenotypes of CRC cells were evaluated by *in vitro* transwell assays and *in vivo* metastatic models. The RNA sequencing was used to explore the downstream targets of ELF4. The luciferase reporter assays and chromatin immunoprecipitation assays were used to ascertain the transcriptional regulation related to ELF4.

**Results:** We found elevated ELF4 was positively correlated with distant metastasis, advanced AJCC stages, and dismal outcomes in CRC patients. ELF4 expression was also an independent predictor of poor prognosis. Overexpression of ELF4 boosted CRC metastasis via transactivating its downstream target genes, fibroblast growth factor receptor 4 (FGFR4) and SRC proto-oncogene, non-receptor tyrosine kinase, *SRC*. Fibroblast growth factor 19 (FGF19) upregulated ELF4 expression through the ERK1/2/SP1 axis. Clinically, ELF4 expression had a positive correlation with FGF19, FGFR4 and SRC, and CRC patients who positively coexpressed FGF19/ELF4, ELF4/FGFR4, or ELF4/SRC exhibited the worst clinical outcomes. Furthermore, the combination of the FGFR4 inhibitor BLU-554 and the SRC inhibitor KX2-391 dramatically suppressed ELF4-mediated CRC metastasis.

**Conclusions:** We demonstrated the essentiality of ELF4 in the metastatic process of CRC, and targeting the ELF4-relevant positive feedback circuit might represent a novel therapeutic strategy.

## Introduction

Colorectal cancer (CRC) is a highly malignant disease, which has taken millions of lives 1. Distant metastasis is the primary reason for CRC-related deaths, and the liver and lung are the most frequently involved organs 2. About 20% of newly diagnosed patients are presented with metastatic CRC (mCRC), which has dismal outcomes 2. Due to the limitations of chemotherapy, the 5-year overall survival (OS) of chemotherapy alone is just 10.8% for mCRC 3. Combination therapy might be a promising way to improve the efficacy of anti-tumor agents and have acceptable toxicity 4. Thus, there is an urgent need for us to unveil the underlying mechanisms of mCRC and develop novel treatment strategies for mCRC.

The human ETS protein family comprises 28 transcription factors that all share the conserved ETS domain. ETS family members are critical regulators of development, cell death, angiogenesis and many other crucial biological processes 5. Increasing evidence indicates that ETS transcriptional factors are involved in tumorigenesis and cancer progression through various mechanisms. For instance, ETV4 increases glycolysis activity and activates Sonic Hedgehog signaling to enhance breast cancer stemness 6. In CRC, ELF3 upregulates the expression of β-catenin and promotes CRC malignant phenotypes 7. As a family member of ETS genes, ELF4 is located on chromosome Xq26, and it plays an essential role in physiological processes like osteogenesis, hematopoiesis and cell cycle regulation 8. Interestingly, several studies demonstrated that the deregulation of ELF4 contributes to the initiation and progression of human cancers. For instance, ELF4 is highly expressed in human glioblastomas (GBM), promotes GBM cell proliferation and stemness, and influences the survival periods of GBM patients 9. In gastric cancer, ELF4 is upregulated by LINC01091, which then transcriptionally upregulates the expression of CDX2 to promote the development of GC 10. In HBV-associated HCC, ELF4 activates telomerase to drive the progression of HCC, and the sphere-forming property of HCC cells is suppressed after ELF4 knockdown 11. Nevertheless, whether ELF4 is involved in the progression of CRC is still elusive.

Fibroblast growth factor receptor 4 (FGFR4) belongs to the tyrosine kinase receptor family, which has been involved in many physiological events, including cell proliferation, differentiation and survival 12. Activation of FGFR4 is initiated by fibroblast growth factors (FGFs) binding to its immunoglobulin domains, resulting in receptor dimerization, tyrosine kinase domains phosphorylation, and finally signaling pathways activation 12. Numerous researches have substantiated the crucial function of FGFR4 in tumor metastasis, including CRC 13. However, the molecular mechanism governing the role of FGFR4 in promoting CRC metastasis is unclear. FGF19 is one of the secreted FGFs, with the highest affinity with FGFR4 among all FGF family members 12-14. It plays a vital role in bile acid synthesis, cellular development and energy homeostasis. It is related to various human diseases, such as obesity, cirrhosis and primary sclerosing cholangitis 15. FGF19 has also been involved in multiple human cancers. In HCC, overexpression of FGF19 is correlated with the adverse outcome of HCC patients. In prostate cancer, FGF19 stimulation enhances the proliferation and invasion of cancer cells, whereas FGF19 knockdown significantly inhibits these malignant phenotypes 16. Contradictorily, FGF19 seems to play tumor-suppressive roles in cholangiocarcinoma and pancreatic carcinoma 15. These findings suggest FGF19 exhibits a complex role in cancer development. Previous finding demonstrates that FGF19 and FGFR4 are coexpressed in CRC and facilitate tumor growth 17. Nevertheless, whether the FGF19-FGFR4 axis is involved in the metastasis of CRC remains unclear, which needs further investigation.

In this study, we uncovered the clinical implications and functions of ELF4 in CRC. FGF19-FGFR4 upregulated ELF4 expression by activating the ERK1/2/SP1 axis, and elevated ELF4 promoted CRC metastasis through upregulating FGFR4 and protein tyrosine kinase SRC expression, which formed the FGF19-ELF4-FGFR4 positive feedback circuit. The FGFR4 inhibitor BLU-554 combined with the SRC inhibitor KX2-391 significantly inhibited ELF4-mediated CRC metastasis.

## Materials and Methods

### Cell lineage

The human CRC lines used in this study including SW480, DiFi, Caco-2, DLD-1, SW620, LoVo, HT29, and HCT116. Those cells were cultured in DMEM culture medium (Gibco) and added with 10% FBS (Gibco) and antibiotics (100ug/ml penicillin-streptomycin) under 5% CO_2_ conditions in 37 °C cell culture incubator.

### In vivo metastatic model

The detailed procedures were described previously 18. Briefly, male 6-week-old BALB/c nude mice were raised and cared for in compliance with our institutional principles for animal care. All experiments involving animals were authorized by the Ethical Committee of the Fourth Military Medical University. Mice were randomly allocated into control or experimental groups. Each group has 10 nude mice.* In vivo* lung metastasis models were constructed via tail vein injections of luciferase-labeled cells (1×10^6^ suspended in 100 μl PBS). To generate liver metastasis mice models, 2×10^6^ luciferase-labeled cells were injected into the spleen of anesthetized mice through a 30-gauge needle. Following the operation in 0 weeks, each mouse received 150 mg/kg of D-luciferin via intraperitoneal injection every other week and bioluminescence signal was detected using an IVIS 100 Imaging System (Xenogen, Hopkinton, MA, USA). The survival status and duration of each mouse were recorded, and all mice were sacrificed at 9 weeks. The lungs and livers of mice were harvested and subjected to further histological examination.

A detailed illustration of the materials and methods was listed in the online supplementary material.

## Results

### Expression profiles of ETS family members in CRC and their influences on CRC cells' migration and invasion

Deregulation of ETS family genes plays a vital role in the initiation, progression and metastasis of human cancers 19. To investigate the potential roles of the ETS family in CRC, we detected the expression levels of 28 ETS family members in 20 pairs of adjacent nontumor tissues, primary, and metastatic CRC tissues. The expression levels of ETS2, ETV4, ETV5, ETV2, ELK1, ETV3, ELF1, ELF4, ELF3, EHF and ETV7 were much higher in CRC than in nontumoral tissues, while ERG, FLI1, SPDEF, ELF2 and SPIB showed the opposite trends. The expression levels of ETS1, ETV1, ELK4, ELK3, ERF, ETV6, SPI1 and GABPA were similar among these 3 kinds of tissues. Besides, the expression of FEV, ETV3L, ELF5, and SPIC was undetected in normal and CRC tissues. Furthermore, among all upregulated genes, the expression levels of ETS2, ETV4, ETV5, ETV2, ELK1, ETV3, ELF4, ELF3, EHF and ETV7 peaked in metastatic CRC, while ELF1 showed no differences between primary and metastatic CRC tissues. Among all downregulated genes, the expression levels of ERG, FLI1, SPDEF, ELF2 and SPIB were much lower in metastatic CRC tissues (**Figure S1**).

To further identify which genes were indispensable for CRC metastasis, we downregulated these 10 ETS genes and upregulated 5 ETS genes in LoVo cells through lentivirus infection, respectively (**Figure S2A, S3A**). Transwell assays demonstrated that the cell mobilities were significantly suppressed after the downregulation of ETV4, ETV5, ELF3, ELF4, ETS2, ELK1, and EHF in LoVo cell lines. In contrast, CRC cells' metastatic and invasive abilities were not changed after the downregulation of ETV2, ETV3 and ETV7 (**Figure S2B**). Furthermore, migratory and invasive properties were significantly suppressed after the upregulation of FLI1, SPDEF and SPIB while enhanced after the upregulation of ERG. ELF2 upregulation didn't change the migrative and invasive phenotype of CRC cells (**Figure S3B**). Besides, ELF4 was the most upregulated ETS gene in our PCR results and had enormous potential for pro-metastasis in CRC cells. Thus, we identified ELF4 as an essential gene for CRC metastasis and focused on the ELF4 gene for further investigation.

### Upregulated ELF4 enhances CRC metastasis and reflects poor prognosis

**Figure 1:**
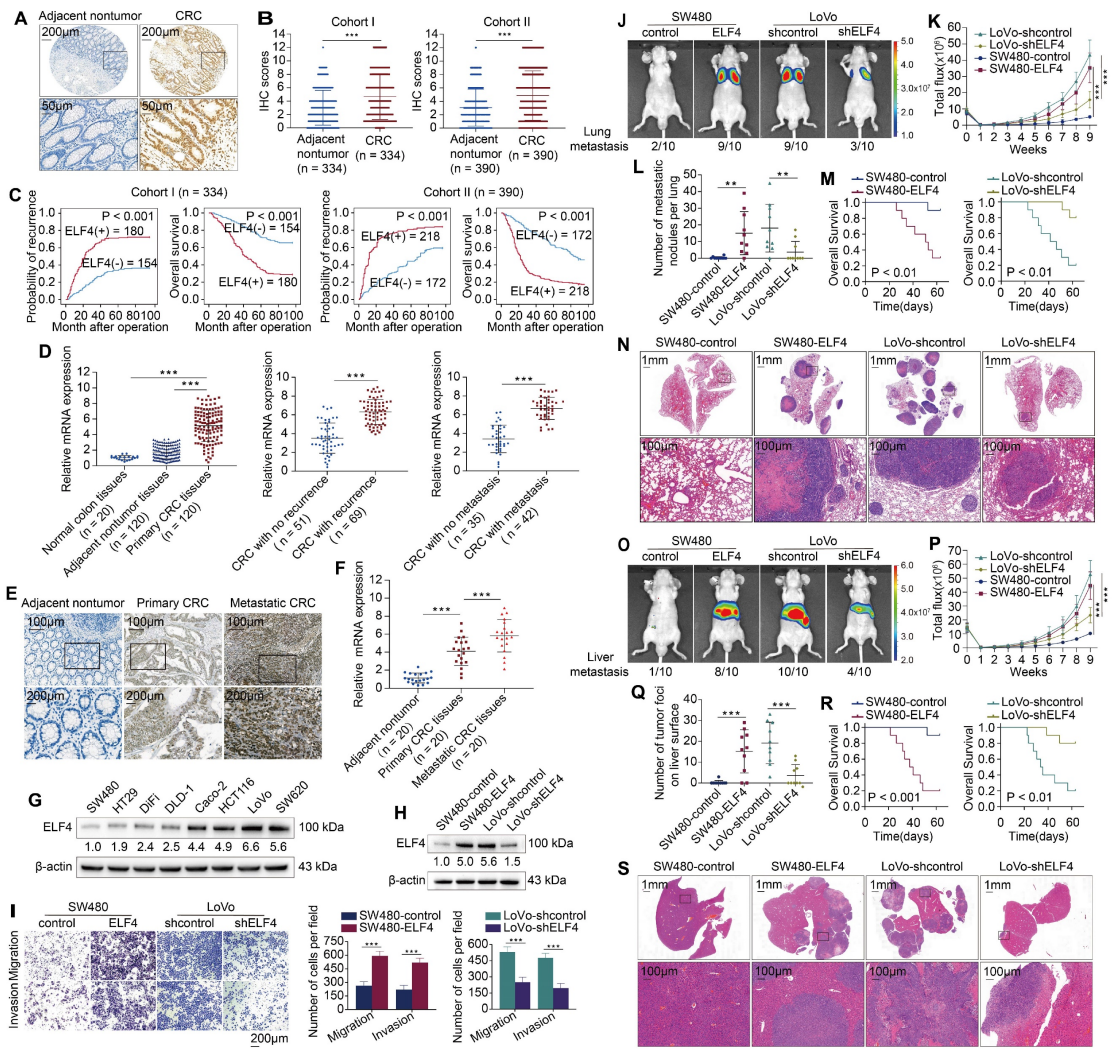
Highly-expressed ELF4 drives CRC metastasis and reflects poor clinical outcomes (A) Representative IHC images of ELF4 expression in CRC and adjacent non-tumor tissues. (B) IHC scores of ELF4 in two independent cohorts derived from CRC patients. (C) KM curves of the association between ELF4 expression and overall survival (OS) or recurrence probabilities of CRC patients. (D) ELF4 expression in the indicated clinical specimens. (E) Representative IHC images of ELF4 expression in nontumor tissues, primary CRC samples, and corresponding metastatic lesions. (F) ELF4 expression in 20 pairs of adjacent nontumor samples, primary CRC samples, and corresponding metastatic lesions. (G) ELF4 expression in multiple CRC cell lines. (H) The efficacy of lentivirus transfection in the indicated CRC cells was determined by immunoblotting. (I) The migratory and invasive phenotypes of the indicated CRC cells were examined by transwell assays. (J-N) ELF4 promotes CRC lung metastasis *in vivo*. (J) Representative bioluminescent graphs and the incidence of pulmonary metastasis in the indicated groups at 9 weeks. (K) The bioluminescent signals of mice in the indicated group were recorded from 0 weeks to 9 weeks. (L) The quantity of pulmonary metastatic nodules of each group. (M) The survival period of mice in each group. (N) Typical histological morphology of pulmonary metastasis in the indicated group. (O-S) ELF4 promotes CRC liver metastasis* in vivo.* (O) Representative bioluminescent graphs and the incidence of liver metastasis in the indicated groups at 9 weeks. (P) The bioluminescent signals of mice in the indicated group were recorded from 0 weeks to 9 weeks. (Q) The quantity of hepatic metastatic nodules in each group. (R) The survival period of mice in each group. (S) Representative histological morphology of liver metastasis in the indicated group. Data are represented as mean±sd. ns, no significance. * p < 0.05, ** p < 0.01. *** p < 0.001.

The overexpression of ELF4 in CRC was also validated through analyzing the TCGA dataset (**Figure S4A**). Immunohistochemistry (IHC) staining was performed to detect ELF4 expression in tissue microarrays constructed from two independent CRC cohorts 18. The results confirmed that ELF4 was highly expressed in CRC samples, while having a relatively low expression level in nontumor specimens (**Figure 1A-B**). In both cohorts, elevated ELF4 was positively correlated with worse tumor differentiation, lymph node metastasis, distant metastasis and higher AJCC stages (**Table S1**). Compared with ELF4-low patients, ELF4-high patients possessed a higher recurrence risk and a diminished lifespan (**Figure 1C**). Furthermore, univariate and multivariate analyses revealed that ELF4 was a crucial independent predictor of poor outcomes (**Table S2**).

Next, the mRNA expression of ELF4 was assessed in 20 nontumor colon samples and 120 primary CRC and corresponding adjacent nontumor specimens. The PCR results showed that ELF4 expression was significantly higher in CRC than in normal colon and adjacent nontumor tissues (**Figure 1D left**). Furthermore, ELF4 was dramatically higher in patients with recurrence or metastasis than in patients without recurrence or metastasis (**Figure 1D middle and right**). The results of the public database were also consistent with our findings. The TCGA dataset showed that higher ELF4 expression was found in CRC samples with lymphatic invasion or perineural invasion compared with samples without invasion, and the results of the GSE41258 dataset indicated ELF4 expression was higher in metastatic liver lesions than in primary CRC specimens (**Figure S4B**). Next, we performed the IHC staining and RT-qPCR of 20 pairs of adjacent nontumor tissues, primary CRC tissues and matched metastatic CRC (mCRC) tissues. The results illustrated that ELF4 increased progressively in nontumor tissues, primary CRC and mCRC (**Figure 1E-F**). Further, the protein levels of ELF4 in CRC cell lines were detected. Interestingly, the results indicated that ELF4 expression was higher in CRC cells with high metastatic properties (LoVo and SW620) than those with low metastatic properties (SW480, DiFi and DLD-1) (**Figure 1G**). The above findings indicated that ELF4 was elevated in highly aggressive CRC cell lines and metastatic CRC tissues, and may play a part in CRC metastasis.

To ascertain whether ELF4 drives CRC metastasis, we transfected SW480 cells with lentiviral ELF4 (LV-ELF4) and transfected LoVo cells with ELF4 shRNA (LV-shELF4). ELF4 overexpression and silencing in the indicated cells were confirmed by immunoblotting (**Figure 1H, Figure S4C**). Transwell assays demonstrated that overexpression of ELF4 significantly enhanced the migrative and invasive abilities of SW480, whereas knockdown of ELF4 in LoVo exhibited the opposite effects (**Figure 1I**).

To elucidate whether ELF4 was related to CRC metastasis *in vivo*, we constructed lung metastatic and liver metastatic models in BALB/c nude mice through tail vein injection and intrasplenic injection, respectively. In lung metastatic models, the ELF4 overexpression group had a higher lung metastasis burden and stronger fluorescence intensity than the SW480-control group (**Figure 1J-L, N**). Consistently, compared with the LoVo-shELF4 group, more metastatic lung nodules and stronger fluorescence intensity were observed in the LoVo-shcontrol group (**Figure 1 J-L, N**). Furthermore, the SW480-control group had a longer overall survival time than SW480-ELF4, while the LoVo-shELF4 group had a prolonged survival time than the LoVo-shcontrol group (**Figure 1M**). Consistent results were also observed in liver metastatic models. The SW480-ELF4 group developed more metastatic liver nodules and stronger fluorescence intensity than the SW480-control group (**Figure 1O-Q, S**). Besides, ELF4 overexpression diminished the survival period of nude mice (**Figure 1R**). Conversely, ELF4 downregulation lowered the number of metastatic nodules in the liver and fluorescence intensity but prolonged the survival time (**Figure 1O-S**). Briefly, these findings demonstrated that ELF4 drove CRC metastasis and was correlated with poor outcomes in CRC patients.

### Pro-metastatic genes FGFR4 and SRC are downstream targets of ELF4

**Figure 2:**
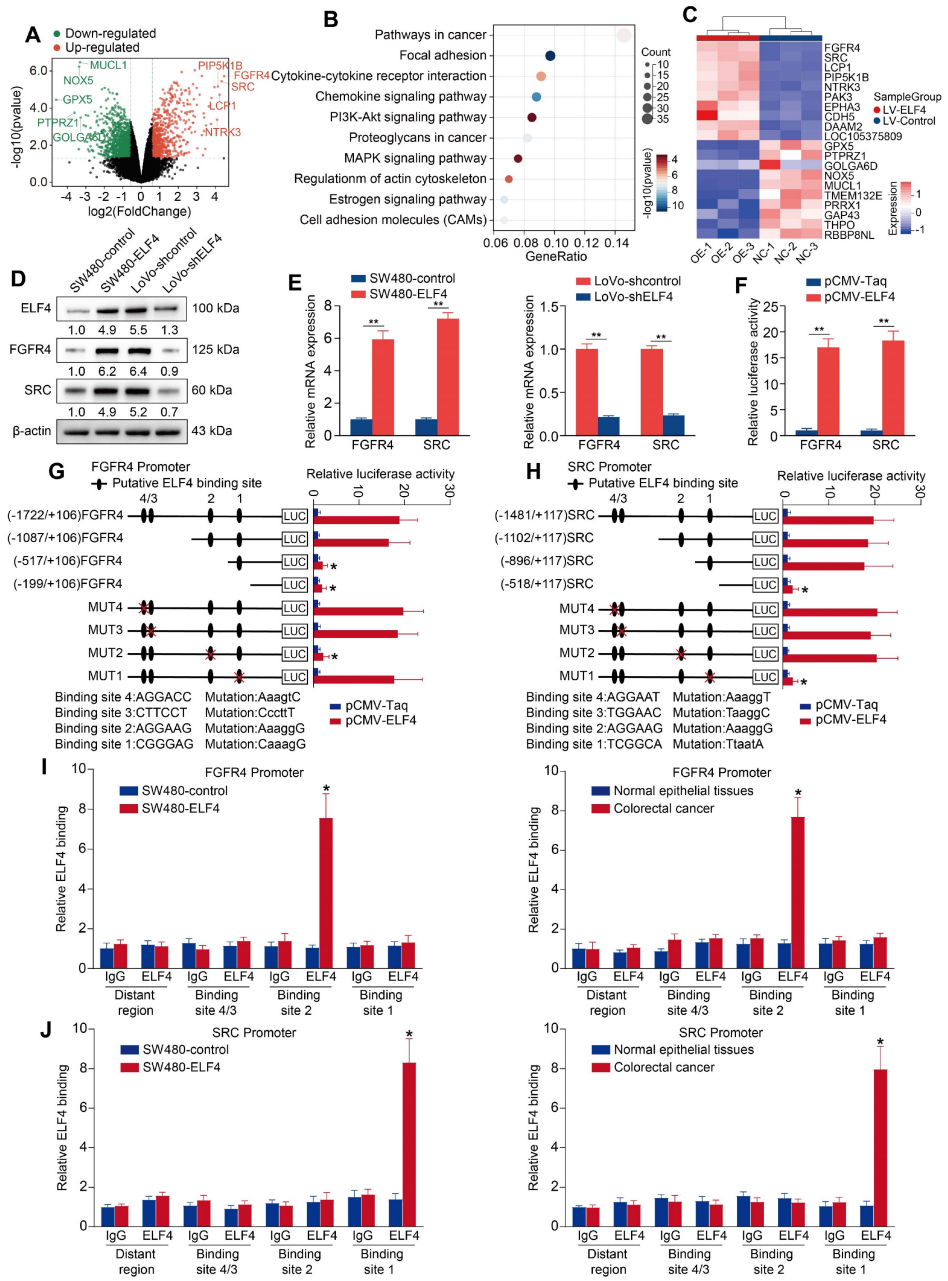
ELF4 transcriptionally upregulates FGFR4 and SRC in CRC (A) The differentially expressed genes (DEGs) between SW480-ELF4 and SW480-control cells were showed by volcano plot. The top 5 most significantly differentially expressed genes were labeled. (B) KEGG analysis of the DEGs between SW480-ELF4 cells and SW480-control cells. (C) Heatmap of the top 10 DEGs. (D-E) The protein and mRNA levels of FGFR4 and SRC in CRC cells transfected with lentivirus were detected by western blotting and RT-qPCR. (F) SW480 cells were co-transfected with pCMV-ELF4 and *FGFR4* or *SRC* promoter luciferase constructs, then promoter activities were analyzed by luciferase reporter assays. (G-H) SW480 cells were co-transfected with pCMV-ELF4 and PGL3-luciferase reporter plasmids containing serially truncated or mutated *FGFR4* (G) or *SRC* (H) promoter constructs and relative luciferase activities were detected. (I-J) ChIP assays demonstrated that ELF4 directly bound to the the *FGFR4* promoter (I) and the *SRC* promoter (J) in both SW480-ELF4 cells and primary CRC specimens. All the data are shown as the mean ± sd. ns, no significance. * p < 0.05, ** p < 0.01. *** p < 0.001.

To further investigate the potential mechanisms of ELF4-mediated CRC metastasis, we extracted total RNA from SW480-control cells and SW480-ELF4 cells for RNA sequencing to determine their transcriptome differences. There were 842 differentially expressed genes (DEGs) from SW480-ELF4 *vs* SW480-control, among which 341 genes were upregulated and 501 genes were downregulated (Fold Change > 2, p < 0.05) (**Figure 2A, Data S1**). Gene ontology (GO) and Kyoto Encyclopedia of Genes and Genomes (KEGG) analysis indicated enriched GO terms and pathways were mainly correlated with tumor metastasis, like focal adhesion, proteoglycans in cancer (**Figure 2B**), and cell migration (**Figure S5, Data S2**). Among 842 DEGs, FGFR4 and SRC were the most upregulated genes upon ELF4 overexpression (**Figure 2C**). Thus, we focused on FGFR4 and SRC for further investigation. PCR and immunoblotting validated that overexpression of ELF4 greatly upregulated the mRNA and protein levels of FGFR4 and SRC, whereas ELF4 silencing significantly downregulated the expression of FGFR4 and SRC (**Figure 2D-E**). Since transcription factors routinely enhance or inhibit the transcription of target genes through binding to their respective promoter, the luciferase reporter assay was applied, which validated that elevated ELF4 enhanced the promoter activities of FGFR4 and SRC (**Figure 2F**).

Several putative ELF4-binding motifs were identified in the promoters of FGFR4 and SRC via the JASPAR database. To determine the roles of these binding sites, we constructed luciferase reporters flanked by truncated or mutated FGFR4 and SRC promoter sequences. The results indicated that loss of the section between -1087 and -517 bp significantly impaired the enhanced activity of the *FGFR4* promoter stimulated by ELF4 overexpression. Furthermore, ELF4-induced FGFR4 promoter activity was significantly abolished by mutating ELF4-binding site 2 in the -1087 to -517 bp region (**Figure 2G**). The same method was used to determine ELF4-dependent transcriptional regulation in the SRC promoter. The results of combined depletion and mutated assays indicated that putative ELF4-binding site 1 located in the sequence between -896 and -518 bp was necessary for ELF4-mediated SRC activation (**Figure 2H**). In addition, chromatin immunoprecipitation (ChIP) confirmed that endogenous ELF4 bound to the identified motifs in the FGFR4 and SRC promoters (**Figure 2I-J**). These results indicated that the effects of ELF4 on CRC metastasis may be mediated by FGFR4 and SRC.

### FGFR4 and SRC act as downstream effectors of ELF4 to promote CRC metastasis

**Figure 3:**
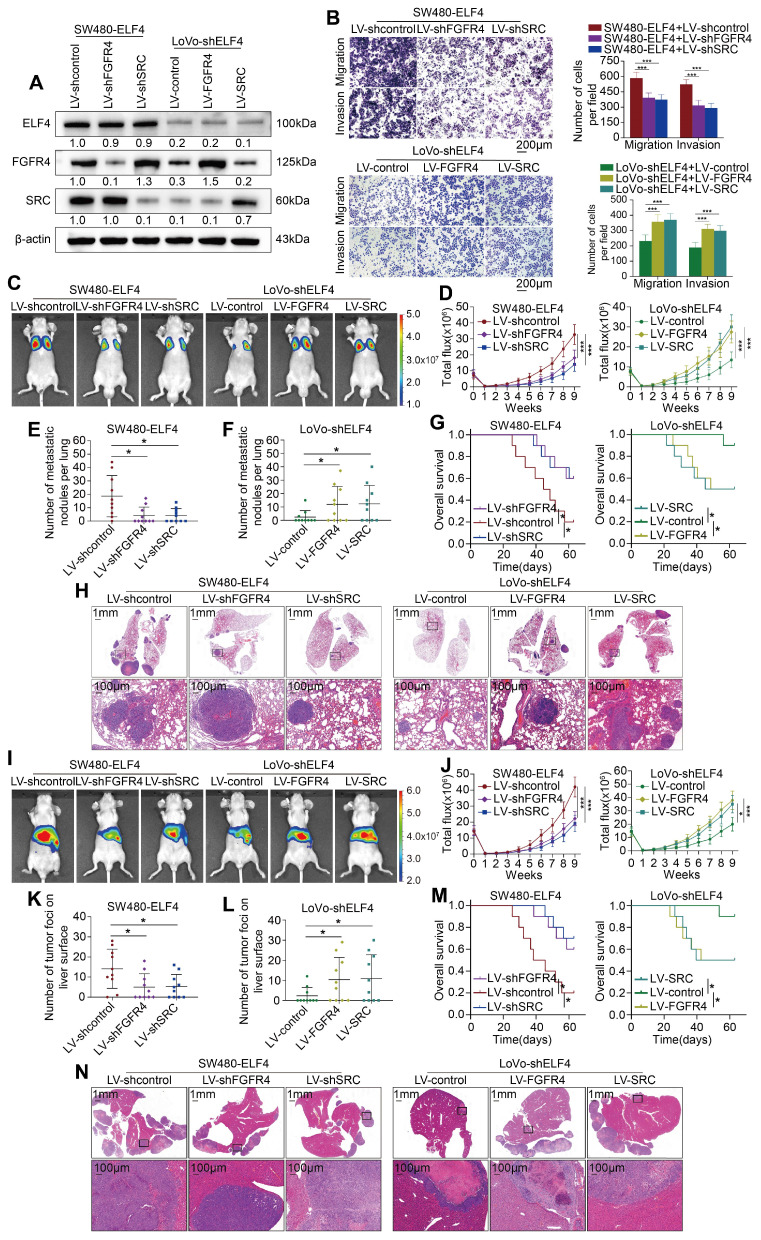
ELF4 facilitates CRC invasion and metastasis by upregulating FGFR4 and SRC (A) ELF4, FGFR4 and SRC expression in the established CRC cell lines was detected by immunoblotting. (B) FGFR4 or SRC downregulation impaired the migratory and invasive phenotypes of SW480-ELF4 cells, while upregulation of FGFR4 or SRC partially rescued the declined migratory and invasive abilities of LoVo-shELF4 cells. (C-H) Pulmonary metastatic assays demonstrated that FGFR4 and SRC were essential for ELF4-mediated CRC lung metastasis. (C) Typical bioluminescent graphs of the different groups at 9 weeks after tail vein injection. (D) The bioluminescent signals of mice in each group were recorded from 0 weeks to 9 weeks. (E) The quantity of pulmonary metastatic nodules in SW480-ELF4 group. (F) The quantity of pulmonary metastatic nodules in LoVo-shELF4 group. (G) Survival period of mice in each group. (H) Typical histological morphology of pulmonary tissues from the different groups. (I-N) Liver metastatic assays demonstrated that FGFR4 and SRC were essential for ELF4-mediated CRC liver metastasis. (I) Typical bioluminescent graphs of the indicated groups at 9 weeks after intrasplenic injection. (J) The bioluminescent signals of mice in each group were recorded from 0 weeks to 9 weeks. (K) The quantity of hepatic metastatic nodules in SW480-ELF4 group. (L) The quantity of hepatic metastatic nodules in LoVo-shELF4 group. (M) Survival period of mice in each group. (N) Typical histological morphology of murine livers from different groups. All the data are shown as the mean ± sd. ns, no significance. * p < 0.05, ** p < 0.01. *** p < 0.001.

To evaluate the roles of FGFR4 and SRC in ELF4-mediated CRC metastasis and invasion, we knocked down the FGFR4 and SRC genes in SW480-ELF4 and upregulated FGFR4 and SRC in LoVo-shELF4 (**Figure 3A**). Transwell assays demonstrated that downregulation of FGFR4 and SRC abrogated ELF4-induced migration and invasion abilities, while upregulation of FGFR4 and SRC attenuated the inhibitory effects caused by ELF4 silencing (**Figure 3B**).

To confirm the role of FGFR4 and SRC in CRC metastasis mediated by ELF4 *in vivo*, we constructed lung metastatic and liver metastatic models in BALB/c nude mice. We found that knockdown of FGFR4 and SRC in SW480-ELF4 cells resulted in fewer incidences of lung metastasis, decreased lung metastatic lesions and weaker fluorescence intensity compared with control cells. Furthermore, the survival period of the SW480-ELF4 group was significantly prolonged after the knockdown of FGFR4 and SRC genes (**Figure 3C-H**). Conversely, the suppressive effects of ELF4 depletion in LoVo cells were partially rescued by upregulation of FGFR4 and SRC. Overexpression of FGFR4 and SRC in LoVo-shELF4 cells promoted CRC cells' lung metastases and strengthened the intensity of fluorescence in the lung, which shortened the OS of nude mice (**Figure 3C-H**). Similar results were observed in liver metastatic models. The SW480-ELF4 group with FGFR4 and SRC knockdown demonstrated fewer liver metastatic nodules and weaker fluorescence intensity, leading to extended OS (**Figure 3I-N**). On the contrary, overexpression of FGFR4 and SRC in LoVo-shELF4 exhibited more liver metastatic nodules and stronger fluorescence intensity, which shortened the OS time (**Figure 3I-N**). Taken together, these findings indicated that FGFR4 and SRC acted as downstream effectors of ELF4 to facilitate CRC metastasis.

### ELF4 expression is positively associated with FGFR4 and SRC expression in human CRC specimens

**Figure 4:**
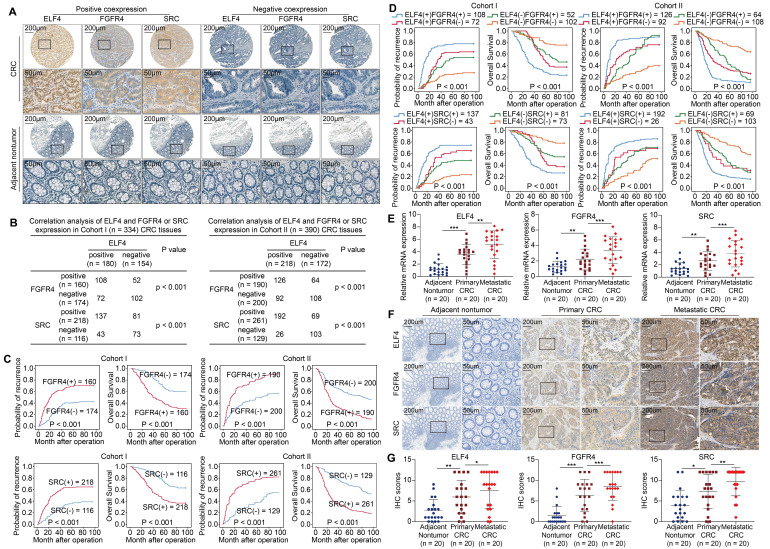
ELF4 expression is positively associated with FGFR4 and SRC expression in CRC specimens (A) Representative IHC images of ELF4, FGFR4 and SRC expression in CRC specimens and adjacent nontumor tissues. (B) Correlation analysis of ELF4 expression and FGFR4 or SRC expression in CRC cohorts. (C) The KM curves of the association between FGFR4 or SRC expression and OS or recurrence rates in two independent CRC cohorts. (D) The association between ELF4 (+)/FGFR4 (+) or ELF4 (+)/SRC (+) and OS or recurrence in two independent CRC cohorts. (E) The relative mRNA expression of ELF4, FGFR4 and SRC in colonic nontumor samples, primary CRC, and corresponding metastatic CRC lesions. (F) Representative IHC images of ELF4, FGFR4 and SRC in normal colonic tissues, primary tumors, and corresponding metastatic lesions. (G) IHC scores of ELF4, FGFR4 and SRC in colonic nontumor tissues, primary tumors, and corresponding metastatic lesions. All the data are shown as the mean ± sd. ns, no significance. * p < 0.05, ** p < 0.01. *** p < 0.001.

To elucidate the clinical significance of FGFR4 and SRC, we analyzed the expression profiles of FGFR4 and SRC in CRC cohorts. The results revealed that FGFR4 and SRC were markedly upregulated in primary CRC compared to adjacent nontumor tissues (**Figure 4A**). Correlation analysis demonstrated that ELF4 was positively associated with FGFR4 and SRC (**Figure 4B**). The expression of FGFR4 or SRC positively correlated with higher AJCC stages, lymph node metastasis, and distant metastasis (**Table S3-S4**). Additionally, compared to patients having negative FGFR4 or SRC expression, those having positive FGFR4 or SRC expression demonstrated higher relapse rates and reduced OS (**Figure 4C**). Furthermore, patients with dual positive ELF4/FGFR4 or ELF4/SRC expression had the worst outcome in both CRC cohorts (**Figure 4D**).

Besides, we assessed the mRNA levels of ELF4, FGFR4 and SRC in 20 matched adjacent nontumor, primary and metastatic CRC specimens. ELF4, FGFR4 and SRC expression were higher in primary CRC compared to adjacent nontumor tissues and peaked in the metastatic specimens (**Figure 4E**). Consistently, the results of IHC also showed trends similar to PCR results (**Figure 4F-G**).

### FGF19-FGFR4 upregulate ELF4 expression through ERK1/2-SP1 signaling pathway

Since FGFR4 is relevant to ELF4-mediated CRC metastasis, its highest affinitive ligand, FGF19, caught our eye. As a prominent oncogene, FGF19 has been implicated in the progression of numerous carcinomas, like thyroid cancer, gastric cancer and lung cancer 20-22. FGF19 is also elevated in CRC and a subset of colon cancer cell lines 17. Stimulating CRC cells with recombinant FGF19 protein significantly promotes CRC proliferation and dissemination, and this enhanced effect is almost entirely reversed by anti-FGF19 monoclonal antibody. Similar findings were made in mouse models implanted with FGF19 knockdown CRC cells 23. These findings highlight the potential role of FGF19 in promoting CRC metastasis. However, the underlying mechanism remains elusive. Thus, we performed bioinformatic analyses of the TCGA-COAD dataset and found that FGF19 was overexpressed in the ELF4-elevated group, and ELF4 was also upregulated in the FGF19-high group (**Figure S6A**). Considering the potential roles of FGF19 in CRC, we wonder if FGF19 and its specific receptor FGFR4 could regulate ELF4 expression and finally facilitate CRC metastasis.

**Figure 5:**
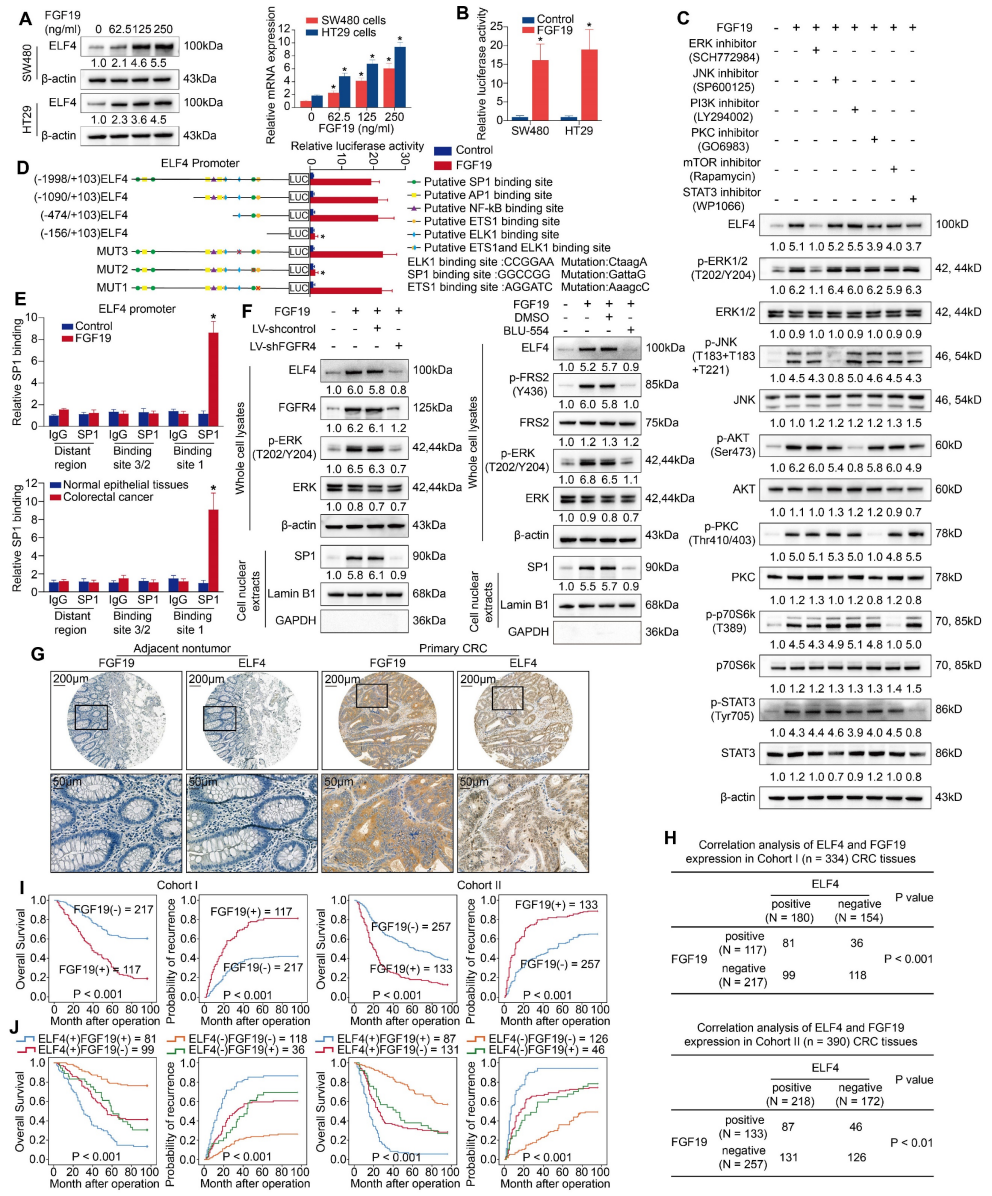
FGF19 upregulates ELF4 expression via the FGFR4-ERK1/2-SP1 signaling pathway (A) SW480 and HT29 cells were exposed to FGF19 of gradient concentrations for 24 hours, and then the mRNA and protein expression of ELF4 in the indicated cells was examined. (B) The ELF4 expression plasmids and the reporter constructs were transfected into SW480 and HT29 cells, then relative luciferase activites were analyzed after FGF19 treatment (250ng/ml, 24h). (C) SW480 cells were exposed to ERK, JNK, PI3K, PKC, mTOR, or STAT3 pathway inhibitors in advance and then treated with or without FGF19. The protein levels of ELF4 and of total and phosphorylated ERK, JNK, AKT, PKC, P70S6K and STAT3 were examined by immunoblotting. (D) SW480 cells were transfected with PGL3-luciferase reporter plasmids containing serially truncated or mutated ELF4 promoter constructs, then cells were exposed to FGF19 (250 ng/ml, 24h), and promoter activities were detected. (E) ChIP assays demonstrated that SP1 could bind to the ELF4 promoter in FGF19-treated SW480 cells and primary CRC specimens. (F) The protein levels of ELF4, p-ERK1/2 and nuclear SP1 in the SW480 cells after transfection with FGFR4 shRNA or exposure to the specific FGFR4 inhibitor, BLU-554, in the presence or absence of FGF19. (G) Representative IHC images of FGF19 and ELF4 expression in adjacent nontumor tissues and CRC tissues. (H) Correlation analysis of FGF19 expression and ELF4 expression in cohort I (upper) and cohort II (lower). (I) The association between FGF19 mRNA level and OS or recurrence of patients in CRC cohorts. (J) The correlation between FGF19/ELF4 co-expression and OS or recurrence of CRC patients. All the data are shown as the mean ± sd. ns, no significance. * p < 0.05.

To explore whether FGF19 triggered ELF4 overexpression, two cell lines with low intrinsic ELF4 expression, SW480 cells and HT29 cells, were exposed to FGF19 for 24 hours. Then, ELF4 expression was assessed by RT-PCR and WB. FGF19 treatment significantly upregulated expression of ELF4 in a dose-dependent manner (**Figure 5A**). The luciferase reporter assay also indicated that the transcriptional activity of the *ELF4* promoter was immensely enhanced after FGF19 treatment (**Figure 5B**). These results illustrated that FGF19 treatment could upregulate the expression of ELF4.

FGF19 is predominantly binding to FGFR4, which is known to stimulate multiple signaling pathways 24. To ascertain which signaling pathway is critical for FGF19-induced ELF4 elevation, we treated SW480 cells with selective inhibitors for FGF19/FGFR4-related downstream signaling pathways. FGF19-induced ELF4 upregulation was significantly abolished after treatment with the ERK inhibitor (SCH772984), while no apparent changes were observed after exposure to other pathway inhibitors (**Figure 5C**). The results of bioinformatic analyses in the TCGA-COAD dataset demonstrated that ERK/MAPK signaling pathway was enriched in the FGF19-high group (**Figure S6B**). These findings suggested that ERK signaling was necessary for FGF19-mediated ELF4 overexpression.

The downstream effector of the ERK signaling involved in FGF19-induced ELF4 expression is still elusive. We analyzed the sequence on the ELF4 promoter and identified some presumed binding sites of transcription factors correlated with the ERK pathway. Next, we constructed luciferase reporter plasmids flanked by truncated or mutated ELF4 promoter sequences and transfected these constructs into SW480 cells. The results indicated that loss of the section between -474 bp and -156 bp significantly diminished the enhanced activity of the *ELF4* promoter induced by FGF19 in SW480 cells, implying this region was critical for FGF19-induced ELF4 expression. Furthermore, ELF4 promoter activity was significantly abolished by disruption of the SP1 binding site located in the -474 bp to -156 bp region, while mutation of the ETS1 or ELK1 binding site in this region had no obvious effects (**Figure 5D**). ChIP assays also validated that SP1 could bind to the ELF4 promoter (**Figure 5E**). To further validate the function of FGF19 is dependent on FGFR4, SW480 cells were transfected with lentivirus shFGFR4 or exposed to BLU-554 (FGFR4 specific inhibitor). The influences of FGFR4 blockade on the ERK1/2/SP1/ELF4 axis were assessed by immunoblotting. To ensure the nuclear protein has been successfully extracted, we chose GAPDH as cytoplasmic reference. The quality of nuclear extracts was confirmed by immunoblot (**Figure 5F**). The phosphorylation of ERK, nuclear translocation of SP1 and ELF4 expression were significantly increased after FGF19 treatment. In contrast, the activation of the FGFR4/ERK1/2/SP1 axis was largely impaired by FGFR4 knockdown or BLU-554 treatment, and the expression of ELF4 was also abolished (**Figure 5F**). The above findings suggested that the ERK1/2/SP1 axis was essential for FGF19-FGFR4 induced ELF4 overexpression.

IHC staining was applied to determine the clinical association of FGF19 and ELF4 in two CRC cohorts. The result indicated FGF19 expression was much higher in CRC than nontumor tissues (**Figure 5G**), and FGF19 had a positive association with worse tumor differentiation, more frequent lymph node metastasis, an increased incidence of distant metastasis and higher AJCC staging (**Table S5**). Correlation analysis indicated that ELF4 positively correlated with FGF19 in CRC cohorts (**Figure 5H**). Survival analysis revealed that high expression of FGF19 reflected a worse prognosis in both two cohorts (**Figure 5I**). The subgroup with positive co-expression of ELF4 and FGF19 demonstrated the worst prognosis among all the groups (**Figure 5J**).

### ELF4 is essential for FGF19-induced CRC metastasis

**Figure 6:**
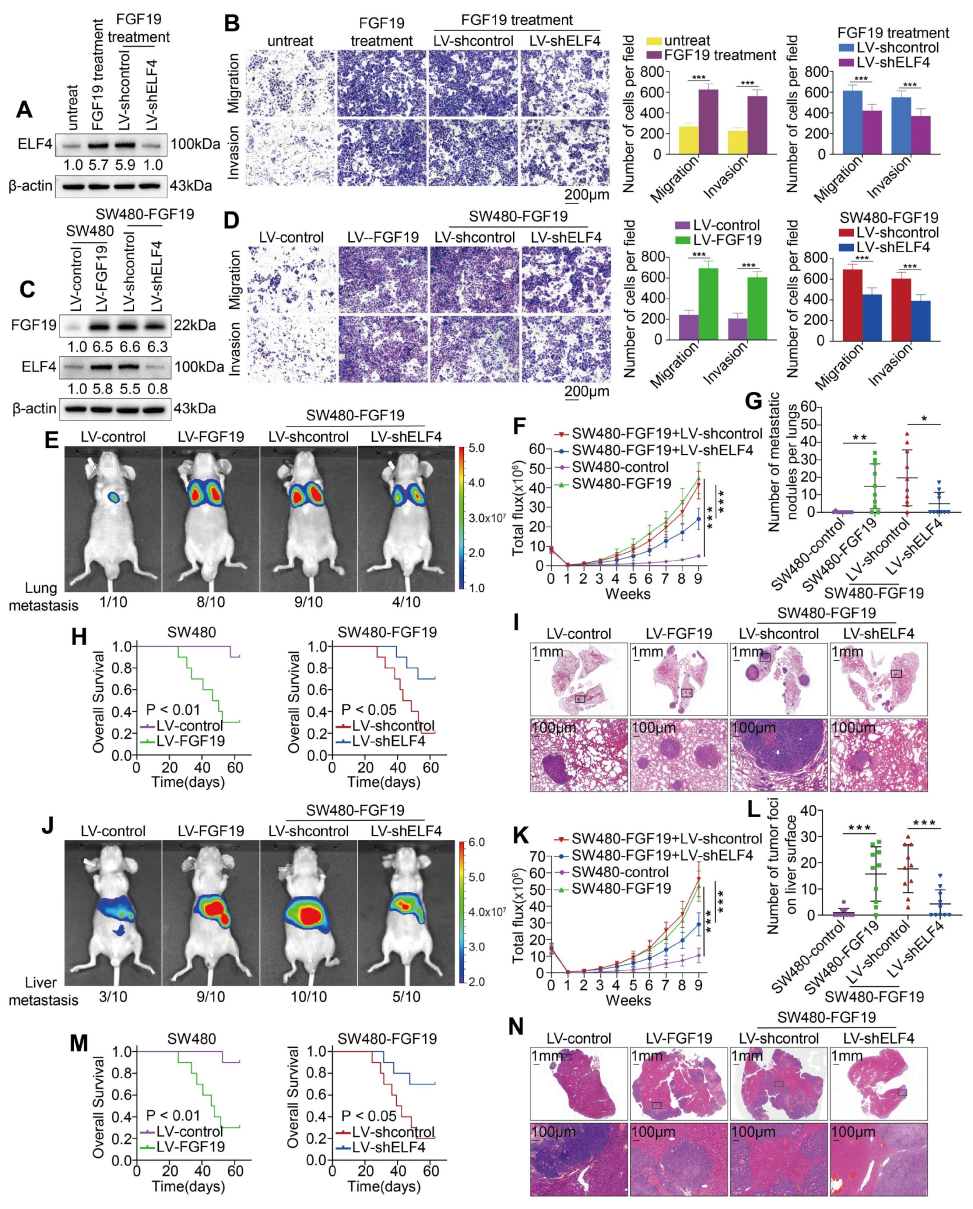
ELF4 is indispensable for FGF19-induced CRC invasion and metastasis (A) SW480-shcontrol and SW480-shELF4 cells were established via lentiviral transfection and then were exposed to FGF19 (250ng/ml, 24 h). The ELF4 protein was detected by immunoblotting. (B) FGF19 exposure dramatically promoted the migration and invasion of SW480 cells while ELF4 silencing impaired FGF19-induced migratory and invasive phenotypes. (C) SW480 cells were transfected with LV-FGF19 lentiviral vectors to construct FGF19-overexpressing SW480 cells (SW480-FGF19), and ELF4 expression was further knockdown via lentivirus-mediated shRNA in SW480-FGF19 cells. FGF19 and ELF4 expression in the indicated cell lines were examined by Western blotting. (D) FGF19 overexpression significantly promoted the migration and invasion of SW480 cells while ELF4 knockdown impaired FGF19-induced migration and invasion in SW480-FGF19 cells. (E-I) Pulmonary metastatic assays demonstrated that ELF4 knockdown significantly impaired FGF19-induced CRC pulmonary metastasis. (E) Representative bioluminescent images and the incidence of pulmonary metastasis in the indicated groups at 9 weeks. (F) The bioluminescent signals of mice in each group were recorded from 0 weeks to 9 weeks. (G) The quantity of lung metastatic nodules of each group. (H) Survival period of mice in each group. (I) Typical histological morphology of pulmonary tissues from the indicated groups. (J-N) Liver metastatic assays demonstrated that ELF4 knockdown significantly impaired FGF19-induced CRC liver metastasis. (J) Representative bioluminescent images and the incidence of liver metastasis in the indicated groups at 9 weeks. (K) The bioluminescent signals of mice in each group were recorded from 0 weeks to 9 weeks. (L) The quantity of liver metastatic nodes of each group. (M) Survival period of mice in each group. (N) Typical histological morphology of hepatic tissues from the different groups. All the data are shown as the mean ± sd. ns, no significance. * p < 0.05, ** p < 0.01. *** p < 0.001.

To ascertain the roles of ELF4 in FGF19-induced CRC metastasis, we established SW480-shELF4 cells and exposed the cells to FGF19 (250 ng/ml, 24h) (**Figure 6A**). FGF19 exposure markedly facilitated the malignant behavior of SW480 cells, while this enhanced effect was further inhibited by ELF4 knockdown (**Figure 6B**). Subsequently, we knocked down ELF4 expression in the FGF19-overexpressing SW480 cell lines (SW480-FGF19). The expression level of FGF19 and the efficacy of ELF4 knockdown in SW480 cells were confirmed by western blotting (**Figure 6C**). Elevated expression of FGF19 enhanced the migration and invasion properties of SW480 cells, whereas the knockdown of ELF4 significantly attenuated the migratory and invasive capabilities promoted by FGF19 (**Figure 6D**).

We also constructed *in vivo* metastatic assays to investigate the role of ELF4 in FGF19-mediated metastasis. *In vivo* lung metastasis assays showed the SW480-FGF19 group demonstrated more lung metastatic foci, a higher incidence of lung metastasis, and stronger fluorescence intensity than the control group (**Figure E-G, I**). Furthermore, the survival period was significantly shortened by overexpression of FGF19 (**Figure 6H**). However, the downregulation of ELF4 significantly reduced the incidence of lung metastasis and the number of metastatic lung foci increased by FGF19, which increased the survival period of mice (**Figure 6E-I**). Similar findings were observed in the liver metastatic models (**Figure 6J-N**). These findings demonstrated that ELF4 was crucial for FGF19-induced CRC metastasis and invasion.

### Combined therapy with FGFR4 inhibitor BLU-554 and SRC inhibitor KX2-391 significantly suppressed ELF4-mediated CRC invasion and metastasis

**Figure 7:**
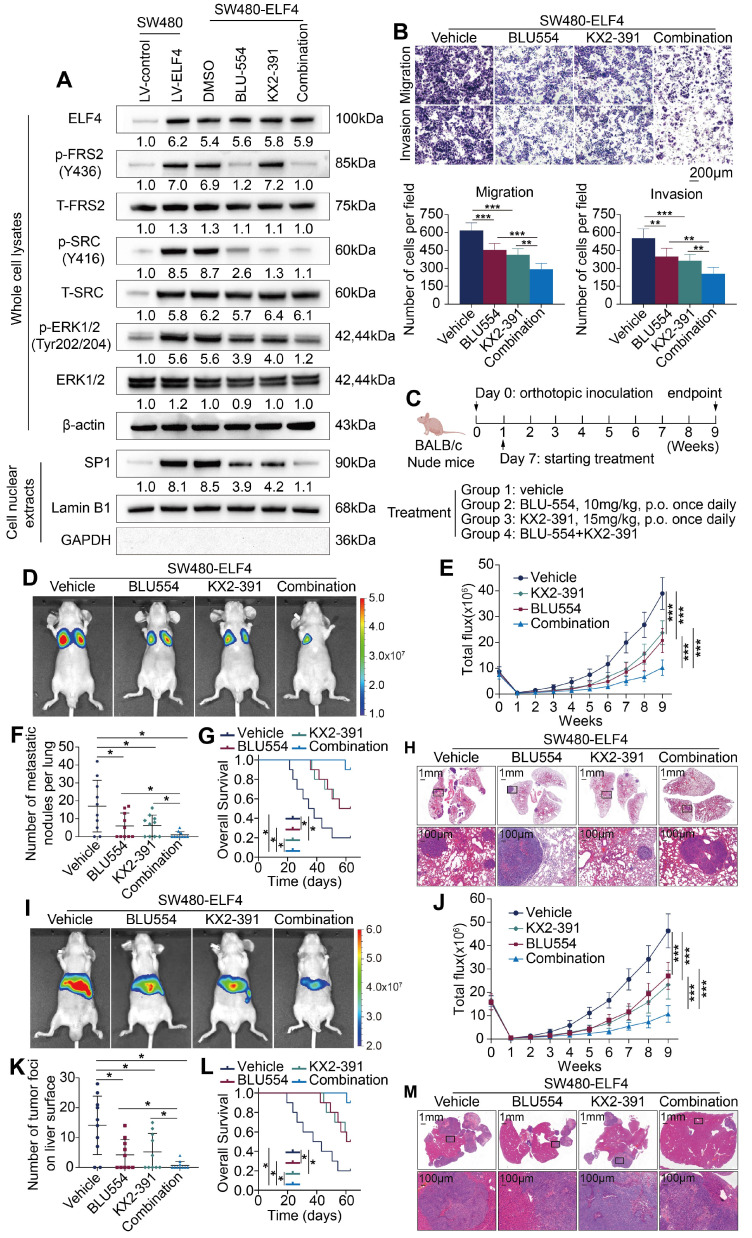
Combination therapy of FGFR4 inhibitor BLU-554 and SRC inhibitor KX2-391 dramatically suppressed ELF4-mediated CRC metastasis (A) SW480-ELF4 cells were treated with BLU-554 (100 nmol/L), KX2-391 (50 nmol/L) alone or combined treatment with KX2-391 plus BLU-554, then the protein levels of ELF4 and the members of relative signaling pathways in the cells receiving distinct treatments were detected by immunoblotting. (B) Combination of BLU-554 and KX2-391 significantly restrained the migratory and invasive phenotypes of SW480-ELF4 cells compared with the vehicle or the monotherapy group. (C) The schematic diagram illustrated the design of the *in vivo* assay. Nude mice were randomly divided into 4 groups (10 mice in each group): vehicle, BLU-554, KX2-391, and BLU-554 plus KX2-391. Treatments were initiated 1 week after inoculation. BLU-554 (10 mg/kg per mouse) and KX2-391 (15 mg/kg per mouse) were administered daily by oral gavage. (D-H) Administration of BLU-554 and KX2-391 significantly impaired ELF4-mediated CRC lung metastasis compared with the vehicle or the monotherapy group. (D) Representative bioluminescent images and the incidence of pulmonary metastasis in the indicated groups at 9 weeks after tail vein injection. (E) The bioluminescent signals of mice in each group were recorded from 0 weeks to 9 weeks. (F) The number of pulmonary metastatic nodules in mice receiving the indicated treatments. (G) Survival period of mice in each group. (H) Typical histological morphology of pulmonary tissues derived from the indicated groups. (I-M) Administration of BLU-554 and KX2-391 significantly impaired ELF4-mediated CRC liver metastasis compared with the vehicle or the monotherapy group. (I) Typical bioluminescent graphs of the indicated groups at 9 weeks after intrasplenic injection. (J) The bioluminescent signals of mice in each group were recorded from 0 weeks to 9 weeks. (K) The number of hepatic metastatic nodules in mice receiving the indicated treatments. (L) Survival period of mice in each group. (M) Typical histological morphology of hepatic tissues derived from the indicated groups. All the data are shown as the mean ± sd. ns, no significance. * p < 0.05, ** p < 0.01. *** p < 0.001.

Our above findings demonstrated that FGF19-mediated ELF4 upregulation enhanced CRC metastatic properties through transactivating FGFR4 and SRC. Therefore, we aimed to investigate whether the combination of BLU-554, a highly specific inhibitor of FGFR4 25, and KX2-391, a novel inhibitor of SRC kinase 26, could affect ELF4-mediated CRC migration and invasion. We first assessed the effects of BLU-554 (100 nmol/L) or/and KX2-391 (50 nmol/L) on protein levels involved in ELF4-relevant pathways. The results indicated that both BLU-554 and KX2-391 effectively restrained the activation of ERK1/2/SP1, whereas combined treatment with BLU-554 and KX2-391 caused more pronounced effects than a single agent (**Figure 7A**). Transwell assays indicated that BLU-554 or KX2-391 treatment alone partially suppressed the migration and invasion properties of SW480-ELF4 cells *in vitro*. However, the combination of BLU-554 and KX2-391 significantly suppressed the migratory and invasive phenotypes of SW480-ELF4 cells compared with the control or monotherapy groups (**Figure 7B**). Next, we performed *in vivo* assays to investigate the roles of BLU-554 or KX2-391 treatment in suppressing ELF4-mediated CRC metastasis in nude mice (**Figure 7C**). Both the results of lung metastasis assays (**Figure 7D-H**) and liver metastasis assays (**Figure 7I-M**) indicated that combination therapy of BLU-554 and KX2-391 markedly reduced the metastasis nodules, the incidence of metastasis, the fluorescence intensity, and more importantly, extended the survival period of the mice compared with vehicle or single-agent therapy. These findings demonstrated that the combination of BLU-554 and KX2-391 was an effective treatment to suppress ELF4-mediated CRC metastasis.

## Discussion

Distant metastasis is the leading cause of CRC-related death. However, about 60% of CRC patients are diagnosed when the lesion is not localized 27. Metastatic CRC (mCRC) is a highly aggressive disease with critically limited overall survival. Consequently, unveiling the potential mechanisms underlying CRC metastasis is vital for developing novel therapies and prolonging the lifespans of CRC patients.

Increasing evidence indicates that ELF4 serves as an oncogene in the progression of multiple cancers. For example, high ELF4 expression promotes neuroblastoma proliferation and maintains poorly differentiated phenotype 28. In endometrial cancer, ELF4 acts as the effector of TRIB3 to drive tumorigenesis 29. On the contrary, ELF4 can inhibit the development of lung cancer and oral carcinoma 30, implying a context-dependent role of ELF4 in cancer. A previous study found that ELF4 expression is significantly suppressed in colon tissues derived from ulcerative colitis, and that reduced ELF4 leads to susceptibility to colitis-associated cancer 31. However, we demonstrated the ectopic expression of ELF4 in CRC, especially in mCRC and CRC cells with high metastatic abilities. The differences in the disease stages may be responsible for the discrepancy. In our study, elevated ELF4 promoted migration and invasion of CRC both *in vitro* and* in vivo*. Clinically, upregulated ELF4 reflected a dismal prognosis and multivariate Cox analyses indicated that ELF4 could serve as an independent prognostic factor for poor outcomes in CRC patients.

Next, we explored the mechanism of ELF4-mediated metastasis. Through analyzing the results of RNA-seq, we found FGFR4 and SRC were the most significantly upregulated genes after ELF4 overexpression. FGFR4 is implicated in nutrient metabolism, tissue repair, and bile acid production in physiological condition 32. Overexpression of FGFR4 has also been involved in the progression of CRC. Upregulated FGFR4 enhances the ability of proliferation and metastasis in CRC and correlates with poor 5-year survival of CRC patients 13. Previous studies reported that accumulated FGFR4 facilitates CRC metastasis through regulating the expression of snail and E-cadherin 33, resulting in the reduced efficacy of cetuximab 34. However, the mechanisms behind FGFR4 upregulation and its role in CRC need further exploration. SRC belongs to the non-receptor tyrosine kinases family and is implicated in the signal transduction initiated by growth factors and cytokines. It is aberrantly upregulated and hyperactivated in most human cancers and correlates with disease progression, including CRC 35. The activity and expression level of the SRC gene product, pp60^c-src^, are dramatically upregulated in liver metastatic lesions compared with primary CRC, highlighting the potential roles of SRC in CRC metastasis 36. It has been reported that overexpressed SRC facilitates the metastatic process of CRC cells 37 and tremendously decreases 5-year survival rates 38*.* Thus, targeting SRC may bring satisfactory clinical benefits to CRC patients. These findings collectively indicated that FGFR4 and SRC exert a crucial role in the metastatic process of CRC. Our study illustrated that elevated ELF4 exerted pro-metastatic effects in CRC by transcriptionally activating its downstream target genes, FGFR4 and SRC. Downregulation of FGFR4 or SRC expression impaired ELF4-mediated CRC metastasis, whereas overexpression of FGFR4 or SRC reversed the impaired metastatic abilities induced by ELF4 knockdown. In clinical CRC specimens, ELF4 was positively correlated with the expression of FGFR4 or SRC. Survival analysis revealed that CRC patients with ELF4/FGFR4 or ELF4/SRC positive coexpression possessed the worst prognosis among all patients in our CRC cohorts. The above findings suggested that ELF4 fostered CRC metastasis and invasion through transactivating FGFR4 and SRC.

FGF19, a specific ligand of FGFR4, is also implicated in various biological and pathological processes 39. The binding of FGF19 and FGFR4 is indispensable for the sorafenib resistance 40. Aberrant activation of the FGF19-FGFR4 axis also enhances metastatic properties in multiple cancers, such as ovarian cancer and HCC 15. In CRC, the FGF19-FGFR4 axis promotes tumor growth through beta-catenin signaling, and the employment of anti-FGF19 antibody (1A6) effectively abolishes this effect 41. However, whether FGF19/FGFR4 is involved in CRC metastasis is largely unknown. Our study identified FGF19 as the upstream regulator of ELF4 and demonstrated that silencing ELF4 inhibited CRC cells' migration and invasion induced by FGF19. We also found that FGF19 was elevated in CRC specimens and reflected poor outcomes in CRC patients. Mechanistically, the FGF19-FGFR4 axis regulated the expression of ELF4 through the ERK1/2-SP1 pathway, which may account for the high expression of ELF4 in CRC. Upregulated ELF4 directly bound to the promoter of FGFR4 and transcriptionally upregulated its expression. Accumulated FGFR4 in the cell membrane caused CRC cells to be more sensitive to FGF19 stimulation, which formed a positive feedback circuit and facilitated CRC cell metastasis. Such a positive feedback loop might be vital in the progression of CRC and could serve as novel targets for developing CRC therapeutic strategies.

ELF4 has long been in the spotlight as an attractive therapeutic target. Downregulation of ELF4 in pancreatic cancer may enhance the efficacy of oncolytic adenovirus treatment 42. Silencing ELF4 in macrophages can improve response to PD1 blockade therapy in lung adenocarcinoma 43. Considering the importance of the FGF19/ELF4/FGFR4 positive feedback circuit in CRC metastasis, we attempted to develop a novel therapeutic strategy to break this loop and achieve satisfactory clinical results. Because there were no available ELF4 inhibitors, we concentrated on the FGFR4 and SRC inhibitors. BLU-554, a highly specific inhibitor of FGFR4, shows potent antitumor effects both *in vitro* and *in vivo* 25. Our study indicated that FGFR4 was a transcriptional target of ELF4, so using BLU-554 may be feasible for treating CRC. KX2-391, also known as Tirbanibulin, is a novel selective SRC inhibitor and has been approved for treating actinic keratosis and psoriasis 44. Numerous studies demonstrated that KX2-391 exhibits robust antitumor effects in multiple cancers, including CRC 45. Besides, through analyzing the dose-response file from the public database, Doris et al. discovered that multiple human cancer cells with high ELF4 expression only showed response to dasatinib, WH-4-023, and ponatinib (all targeting SRC), while being resistant to the majority of anti-cancer agents 46. Combined with the discovery that ELF4 was highly expressed in CRC, these findings highlight the potential for treating ELF4-overexpressing CRC with KX2-391. Unfortunately, the results of phase I/II clinical trials indicated that KX2-391 treatment provided no or limited benefit to patients with solid tumors 47. Similar results were found in another SRC inhibitor, dasatinib 48. On the one hand, this unsatisfactory effect of SRC inhibitors may be due to the lack of patient stratification based on ELF4 expression in the treatment cohorts. On the other hand, a previous study revealed the crosstalk between FGFR4 and SRC, which indicated that FGFR4, SRC and STAT3 could collectively form an endosomal complex to modulate the expression and activity of each other in HCC 49. The above finding implied that the reciprocal relationship between FGFR4 and SRC in human cancer may be responsible for the limited clinical benefits obtained from the FGFR4 or SRC inhibitor monotherapy. Besides, the reciprocal relationship of FGF19/FGFR4 and other genes was also found in multiple cancers. Our team previously elaborated the SRY-related high-mobility group box 18 (SOX18) gene acts as the downstream executor of FGF19/FGFR4 and SOX18 in turn upregulates the expression of FGFR4, which formed FGF19-SOX18-FGFR4 positive circuit to facilitate the metastasis of hepatocellular carcinoma 50. In Helicobacter pylori-mediated gastric cancer, the FGF19/FGFR4 axis increased the level of STAT3, while elevated STAT3 could promote the transcription of FGFR4 and form the FGF19-STAT3-FGFR4 positive feedforward loop 51. In lung cancer, GLI Family Zinc Finger 2 (GLI2) acts as the downstream gene of FGF19/FGFR4, and GLI2 can directly transcriptionally upregulate FGF19 expression, forming the FGF19-GLI2-FGF19 feedback loop to promote metastasis 52. Based on these facts, we speculated that the efficacy of simultaneously inhibiting FGFR4 and SRC may be superior to targeting FGFR4 or SRC alone. Our study indicated that combined usage of BLU-554 and KX2-391 significantly inhibited ELF4-mediated CRC metastasis *in vitro* and *in vivo* compared with vehicle or monotherapy treatment, highlighting the potential for BLU-554 and KX2-391 combination therapy in the treatment of mCRC. These results provided a rationale for the combination of BLU-554 and KX2-391 in treating ELF4-mediated CRC metastasis.

Unfortunately, there are still distant metastases after extended exposure to both BLU-554 and KX2-391, the mechanisms of which may include activation of alternative signaling pathways and drug target mutation. Previous study suggested that the c-Met signaling parallelly activated in gastric cancer cell lines may confer resistance to Src inhibitors 53. PI3K and MEK pathways were also reported to be reactivated following prolonged Src inhibitor treatment 54. The drug treatment may also select for clonal expansion of the tumor subtypes dependent on other oncogenic factors, such as protein kinase Cι, leading to eventual tumor progression and mortality 55. The inhibitory effect of BLU-554 is exerted by binding to the Cys552 of the ATP pocket of FGFR4 56. However, FGFR4 mutations may occur during treatment with BLU-554, leading to acquired drug resistance and compromised inhibitor efficacy. HCC patients who initially responded but ultimately progressed on BLU-554 treatment were reported to harbor gatekeeper and hinge-1 mutations in the tyrosine kinase domain of FGFR4, which may disturb the binding of BLU-554 57. Even worse, FGFR4 mutations were observed to autoactivate the receptor and facilitate the metastasis of cancer 58, 59. The functional status of autophagy pathway may also play a role in the resistance to the inhibitors 60. Although these findings on other tumor types may provide clues to the residual malignancy of CRC, further studies should be performed to address these issues and to design novel inhibitors capable of overcoming the limited efficacy.

**Figure 8:**
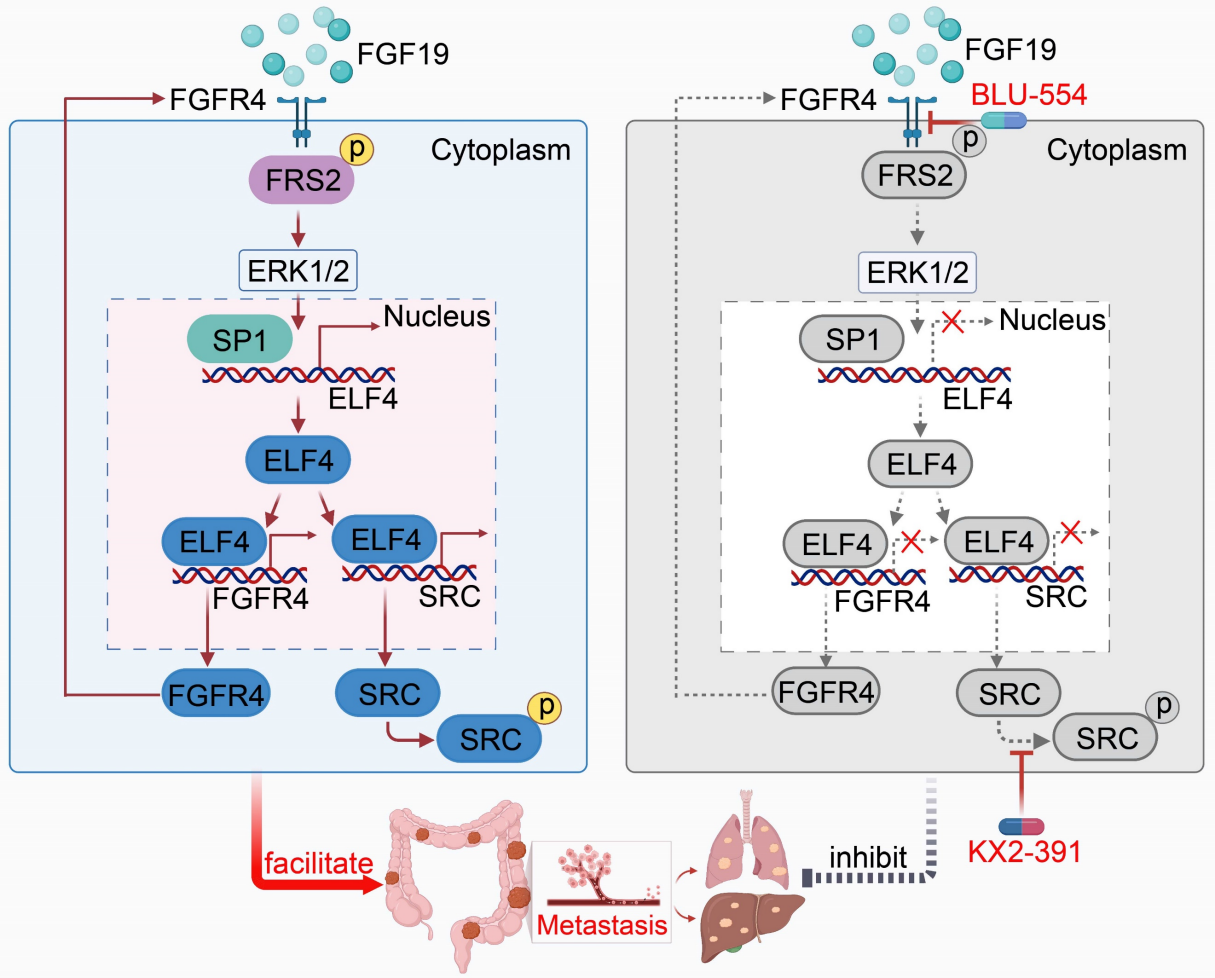
An illustration depicting the function of the FGF19-ELF4-FGFR4 positive feedback circuit in CRC metastasis. The FGF19-FGFR4 axis upregulates ELF4 expression by activating the ERK1/2/SP1 signaling pathway. ELF4 upregulation boosts CRC invasion and metastasis via transactivating FGFR4 and SRC. Combined treatment of FGFR4 inhibitor, BLU-554, and SRC inhibitor, KX2-391, effectively breaks the FGF19-ELF4-FGFR4 positive feedback circuit and has potent inhibitory effects on ELF4-mediated CRC metastasis.

Taken together, we found the FGF19-FGFR4 upregulated the expression of ELF4 via the ERK1/2/SP1 axis, and overexpression of ELF4 facilitated CRC metastasis through transactivating FGFR4 and SRC, which formed a positive feedback loop. Combined targeting of FGFR4 and SRC effectively broke the positive feedback circuit and markedly suppressed CRC metastasis. Our study may provide new insights for understanding the potential mechanism of CRC metastasis and may help develop promising strategies for CRC treatment (Figure 8).

## Supplementary Material

Supplementary methods, figures, tables, and data.

## Abbreviations

CRC: colorectal cancer
ELF4: E74-like factor 4
FGFR4: fibroblast growth factor receptor 4
FGF19: fibroblast growth factor 19
SRC: SRC proto-oncogene, non-receptor tyrosine kinase
IHC: immunohistochemistry
mRNA: messenger RNA
shRNA: short hairpin RNA
BLI: bioluminescent imaging
ChIP: chromatin immunoprecipitation
H&E: hematoxylin and eosin
ERK: extracellular regulated protein kinase
PI3K: phosphoinositide 3-kinase
mTOR: mechanistic target of rapamycin kinase
SP1: Sp1 transcription factor
ETS1: ETS Proto-Oncogene 1, Transcription Factor
ELK1: ETS Transcription Factor ELK1
siRNA: small interfering RNA
EMT: epithelial-mesenchymal transition
TNM: tumor-nodule-metastasis
GO: Gene ontology (GO)
KEGG: Kyoto Encyclopedia of Genes and Genomes
OS: overall survival
KM: Kaplan-Meier
