# Supplementary material for: FGF19-mediated ELF4 overexpression promotes colorectal cancer metastasis through transactivating FGFR4 and SRC
Source: Theranostics. 2023 Feb 22;13(4):1401–18. doi: 10.7150/thno.82269 (PMC10008733; doi:10.7150/thno.82269)
Supplement: Supplementary file 1 — Supplementary methods, figures, tables, and data. [file thnov13p1401s1.pdf]

Supplementary materials for

FGF19-mediated ELF4 overexpression promotes colorectal cancer metastasis through transactivating FGFR4 and SRC

Xilang Chen[1,#], Jie Chen[1,#], Weibo Feng[1,#], Wenjie Huang[2,3,#], Guodong Wang[1,#], Mengyu Sun[2], Xiangyuan Luo[2], Yijun Wang[2], Yongzhan Nie[1], Daiming Fan[1,*], Kaichun Wu[1,*], Limin Xia[1,2,*]

***Correspondence:** Dr. Limin Xia (xialimin@tjh.tjmu.edu.cn)

This PDF file includes:

Materials and Methods

Supplementary Figures 1 to 6

Supplementary Tables 1 to 8

Supplementary Data 1 to 2

References

Materials and Methods

Cell culture

All cells used in this study were obtained from the American Tissue Type Culture Collection. Each cell line was tested and authenticated by their manufacturers. The cells were cultured in Dulbecco's modified Eagle's medium (Gibco, Thermo Fisher Scientific, Cambridge, MA, USA) supplemented with 10% fetal bovine serum (FBS, Gibco), 100 μg/ml penicillin, and 100 μg/ml streptomycin (Gibco) in a 5% CO_2 atmosphere at 37 °C. These above cell lines were authenticated by short tandem repeats (STRs) DNA profiling. All cells were tested for mycoplasma contamination before use with the Universal Mycoplasma Detection Kit (ATCC 30-1012K) and were not contaminated by mycoplasma.

Plasmid construction

All plasmid generation was carried out based on previously published standard procedures. Briefly, for example, the ELF4 gene complete CDS construct, pCMV-ELF4, was generated by using cDNA from human PBMCs. It was generated with forward and reverse primers incorporating EcoRI and XhoI sites at the 5' and 3'-ends, respectively. The final polymerase chain reaction (PCR) product was cloned into the EcoRI and XhoI sites of the pCMV-Tag2B vector. Besides, human genomic DNA was used to produce the FGFR4 promoter construct, (-1722/+106) FGFR4, which corresponds to the -1722 to +106 sequence (relative to the transcriptional start site) of the 5'-flanking region of the human FGFR4 gene. Forward and reverse primers containing the SacI and XhoI

sites at the 5' and 3'-ends, respectively, were used to build the construct. The final PCR product was cloned into the SacI and XhoI sites of the pGL3-Basic vector (Promega). Similarly, progressive deletion constructs of the FGFR4 promoter, (-1087/+106)FGFR4, (-517/+106)FGFR4, (-199/+106)FGFR4, were generated using the (-1722/+106)FGFR4 construct as the template. The QuikChange II Site-Directed Mutagenesis Kit (Stratagene, CA, USA) was used to mutate the ELF4 binding sites in the FGFR4 promoter. The sequence integrity of all constructs was verified by gene sequencing. All promoter constructs used in this experiment were generated similarly. All primers are listed in **Table S6**.

Construction of lentivirus and stable cell lines

Lentivirus production was performed according to a previously described protocol. Lentiviral vectors encoding shRNAs were generated using PLKO.1-TRC (Addgene) and designated as LV-shELF4, LV-shFGFR4, LV-shSRC, and LV-shcontrol. "LV-shcontrol" is a non-target shRNA control. The vector "pLKO.1-puro Non-Target shRNA Control Plasmid DNA" (purchased from Sigma, SHC016) contains an shRNA insert that does not target any known genes from any species. Lentiviral vectors encoding the human ELF4, FGFR4, SRC and FGF19 genes were constructed in pLenti-CMV-GFP-Neo or pLenti-CMV-GFP-Puro (Addgene) and designated as LV-ELF4, LV-FGFR4, LV-SRC and LV-FGF19. An empty vector was used as the negative control and was designated as LV-control. The lentivirus and cell infection were produced according to the lentiviral vector protocol recommended by Addgene. Briefly, the

lentiviral plasmid and packaging plasmids pMD2. G and psPAX2 (Addgene plasmid #12259 and #12260) were transfected into HEK-293T cells with transfection reagent (Lipofectamine®3000, Thermo Fisher Scientific) and OPTI-MEM media (Invitrogen, Waltham, MA, USA). The lentiviruses were harvested twice on days 4 and 5. Viruses were filtered with a 0.45-μm filter and stored at -80 °C. Lentiviral infection of target cells were performed in cell culture media with 5 μg/ml polybrene (Sigma H9268). Seventy-two hours after infection, cells were selected for 2 weeks using 2.5 μg/ml puromycin (OriGene). Selected pools of cells were used for the following experiments. All shRNA was listed in **Table S7**.

Transient transfection

A total of 1×10^5 serum-starved cells were plated in each well of a 24-well plate and allowed to attach for 12-24 hours. Then, a mixture of Lipofectamine 2000 (Invitrogen, USA) containing 0.02 μg of the pRL-TK plasmids, 0.18 μg of the promoter reporter plasmids and 0.6 μg of the expression vector plasmids was used to co-transfect cells for 5 hours based on the manufacturer's instructions. The cells were then washed and incubated with 1% FBS-supplemented fresh medium for 48 hours.

Luciferase reporter assay

The Dual Luciferase Assay (Promega, USA) was used to quantify luciferase activity following the manufacturer's instructions. Transfected cells were subjected to cell lysis in a culture dish with lysis buffer. Subsequent lysates were transferred to an Eppendorf

microcentrifuge before being centrifuged for 1 minute at maximum speed. The efficiency of transfection was normalized to Renilla activity, and relative luciferase activity was quantified with a Modulus TM TD20/20 Luminometer (Turner Biosystems, USA).

Reagents

ERK inhibitor SCH772984, JNK inhibitor SP600125, PI3K inhibitor LY294002, PKC inhibitor GO6983, mTOR inhibitor Rapamycin, and STAT3 inhibitor WP1066 were purchased from MedChemExpress. KX2-391 and BLU-554 were purchased from Selleck (Houston, TX). Recombinant human FGF19 protein was provided by R&D SYSTEMS. The agents were used under the standard protocols.

***In vitro* migration and invasion assays**

The invasive and migratory capabilities of each cell line were assessed with an 8-μm pore, 24-well Transwell plate (Corning, USA). For invasion assays, chamber inserts were first coated with 60 μL of Matrigel (Corning, 200 mg/mL) and left to dry overnight under sterile conditions. The next day, the uppermost chamber was plated at a cell density of 1×10^5. For cell migration assays, the upper chamber, which was lined with a noncoated membrane, was plated with cells at a density of 1×10^5. To examine the effect of SRC and FGFR4 inhibitors on migration and invasion of CRC cells, the cells were pre-treated with KX2-391 (50 nmol/L) for 24 hours, or BLU-554 (100 nmol/L) for 48 hours. Each assay was repeated thrice, and three different inserts were used to obtain a mean cell number in five fields per membrane.

Western blot analyses

The detailed procedures were described previously [1]. Proteins from lysed cells were fractionated by SDS-PAGE and transferred to nitrocellulose membranes. Nonspecific binding sites were blocked with 5% milk in TBST (120 mM Tris–HCl (pH 7.4), 150 mM NaCl, and 0.05% Tween 20) for 1 hour at room temperature. Blots were incubated with a specific antibody overnight at 4 °C. Western blotting of β-actin on the same membrane was used as a loading control. The membranes were incubated with primary antibodies overnight at 4 °C. The membranes were then washed with PBS 3 times and incubated with an HRP-conjugated secondary antibody. Proteins were visualized using a Immobilon^{TM} Western Chemiluminescent HRP substrate (Millipore, USA).

The primary antibodies used are listed below.

Antibodies	Source
anti-ELF4	Invitrogen, OTI2D8
anti-FGFR4	R&D, MAB6852
anti-SRC	Cell Signaling Technology, 2109
anti-FGF19	Cell Signaling Technology, 83348
anti-SP1	Cell Signaling Technology, 9389
anti-Lamin B1	Cell Signaling Technology, 17416
anti-PKCζ	Cell Signaling Technology, C24E6
anti-p-PKCζ(Thr410/403)	Cell Signaling Technology, 9378
anti-Akt(pan)	Cell Signaling Technology, 4685

anti-p-Akt (Ser-473)	Cell Signaling Technology, 4060
anti-JNK	Cell Signaling Technology, 9252
anti-p-JNK(T183+T183+T221)	Cell Signaling Technology, 9255
anti-p-p70S6K(T389)	Cell Signaling Technology, 9234
anti-p70S6K	Cell Signaling Technology, 2708
anti-p-ERK1/2(T202/Y204)	Cell Signaling Technology, 4370
anti-ERK1/2	Cell Signaling Technology, 9102
anti-p-STAT3 (Tyr705)	Cell Signaling Technology, 9145
anti-STAT3	Cell Signaling Technology, 9139
anti-FRS2	Abcam, ab183492
Anti-p-FRS2(Y436)	Cell Signaling Technology, 3861
anti-β-actin	Proteintech, 66009-1-Ig

Real-time qPCR

The RNeasy Plus Mini Kit (50) kit (Qiagen, Hilden, Germany) was used to extract total RNA, which was then reverse transcribed with the Advantage RT-for-PCR Kit (Qiagen) in accordance with the manufacturer's protocols. The target sequence was amplified with real-time PCR with the SYBR Green PCR Kit (Qiagen). The cycling parameters used were 95 °C for 15 s, 55-60 °C for 15 s, and 72 °C for 15 s for 45 cycles. Melting curve analyses were performed, and Ct values were determined during the exponential amplification phase of real-time PCR. SDS 1.9.1 software (Applied Biosystems, Massachusetts, USA) was used to evaluate amplification plots. The $2^{-\Delta\Delta Ct}$

method was used to determine relative fold changes in target gene expression in cell lines, which was normalized to expression levels in corresponding control cells (defined as 1.0). The equation used was $2^{-\Delta\Delta Ct}$ ($\Delta Ct = \Delta Ct^{target} - \Delta Ct^{GAPDH}$; $\Delta\Delta Ct = \Delta Ct^{expressing\ vector} - \Delta Ct^{control\ vector}$). When calculating relative expression levels in surgically extracted CRC samples, relative fold changes in target gene expression were normalized to expression values in normal colon epithelial tissues (defined as 1.0) using the following equation: $2^{-\Delta\Delta Ct}$ ($\Delta\Delta Ct = \Delta Ct^{tumor} - \Delta Ct^{nontumor}$). All experiments were performed in duplicate. **Table S6** lists all sequences of all primers used.

Patients and follow-up

The details of construction of CRC cohorts were described previously [2]. Written informed consent was obtained from each patient, and ethical approval was obtained from the Ethics Committee of the Fourth Military Medical University. Cohort I included freshly sampled CRC tissues with healthy adjacent tissues collected between January 2005 and December 2007 from 334 adult patients who underwent surgery at Xijing Hospital of the Fourth Military Medical University (Xi'an, China). Cohort II included CRC tissue samples that were surgically resected from 390 adult CRC patients between January 2005 and December 2007 at the Tongji Hospital of Tongji Medical College (Wuhan, China). All patients were staged pathologically based on the American Joint Committee on Cancer (AJCC)/International Union against Cancer criteria. All patients were preoperative radiotherapy- and chemotherapy-naïve; However, those with stage II–IV disease received postoperative adjuvant chemotherapy. No patients were treated

with postoperative radiotherapy. Primary tumor samples along with dissected regional lymph nodes were subjected to histomorphological analysis via hematoxylin–eosin (H&E) staining performed by the Department of Pathology of Xijing and Tongji Hospital. ELF4 mRNA expression was assessed in 120 pairs of frozen fresh CRC tissues and peripheral nontumor tissues that were collected during surgical resection and frozen in liquid nitrogen.

The information collected during the follow-up period included the incidence of disease recurrence and the presence of distant metastasis as confirmed by imaging and procedural data (position emission tomography, ultrasonography, magnetic resonance imaging, computed tomography and endoscopy) or pathological data (biopsies and cytologic analysis). Overall survival time was defined as the period between surgical resection and death. The duration of disease-free survival was defined as the period between surgical resection and the emergence of either distant CRC metastasis or CRC recurrence, the occurrence of another noncolorectal cancer (with the exception of carcinoma in situ of the cervix and skin basal cell carcinoma) or death from any cause without documentation of a cancer-related event. Patients were followed up for a minimum of 8 years, with follow-up data collected via questionnaire letters and telephone inquiry; patient databases were updated every 3 months. Patient deaths were determined by a corroborative history from the family and verified by reviewing public records.

Furthermore, 20 pairs of fresh CRC tissues and adjacent nontumor tissue samples and 20 matched metastatic CRC tissues were collected after surgical resection for

further investigations.

Construction of tissue microarrays and immunohistochemical (IHC) staining

Tissue microarrays were constructed with the sampled human CRC tissues and their respective adjacent healthy tissues (Shanghai Biochip Co., Ltd. Shanghai, China). These microarrays were analyzed for ELF4 (Invitrogen, OTI2D8, 1/200), FGFR4 (Abcam, ab262838, 1/200), SRC (Cell signaling technology, #2109, 1/400), and FGF19 (cell signaling technology, #83348, 1/200) expression. All tissue microarrays were independently scored based on the degree of target protein expression and staining intensity by two pathologists who were blinded to the sample identities.

Sections of samples that were 4-μm thick were embedded in paraffin and used for IHC staining; paraffin-embedded sections were routinely processed. Briefly, slides were first incubated on a 60 °C heating panel for one hour before the paraffin was removed with xylene, and the sections were rehydrated using the gradient ethanol immersion technique. The sections were exposed to 3% (vol/vol) hydrogen peroxide for 12 minutes in methanol to quench endogenous peroxidase activity. The sections were then washed with PBS thrice for three minutes each time. Subsequently, the slides were placed into a microwave for half an hour while submerged in a 0.01 mol/L citrate buffer solution (pH 6.0). After the final PBS (pH 7.4, 0.01 mol/L) wash, the slides were incubated overnight with their primary antibodies diluted in PBS containing 1% (wt/vol) bovine serum albumin in a damp chamber at 4 °C. To produce negative controls, the same procedure was repeated on a separate set of slides, but preimmune mouse serum was used in place of the primary antibody. The following day, the slides were first

washed thrice for 5 minutes each with PBS and exposed to a peroxidase-conjugated second antibody for half an hour (Dako, Carpinteria, CA, USA) at room temperature, followed by another three cycles of 5-minute PBS washes. Diaminobenzidine exposure for 2 minutes was used to visualize the reaction product, and images were captured with a DP70 digital camera-equipped light microscope (Olympus, Japan).

Analyses were performed by two independent observers who were blinded to the clinical outcome. The percentage of positive cells was scored on a scale of 0 to 4: 0 (negative), 1 (1%-25%), 2 (26%-50%), 3 (51%-75%), or 4 (76%-100%). The intensity of the immunostaining was scored on a scale of 0 to 3: 0 (negative), 1 (weak), 2 (medium) or 3 (strong). The product of the above two scores was used as the total immuno-activity score, which ranged from 0 to 12. The cut-off point for a 'high' score was a final score equal to or greater than 4, while scores from 0 to 3 were considered 'low'.

***In vivo* metastatic model and bioluminescence imaging**

Six-week-old BALB/c nude mice were cared for and maintained based on our institution's protocols for ethical animal care. The Committee on the Use of Live Animals in Teaching and Research (CULATR) of the Fourth Military Medical University approved all animal experiments. Mice were randomly assigned into experimental or control groups, blinding was not possible. In the tail vein injection-based *in vivo* metastasis assays, 10 mice in each group received tail vein injections of 1×10^6 cells in 100 μL of phosphate-buffered saline (PBS). In the intrasplenic injection-

based *in vivo* metastasis assays, the mice were first anesthetized by intraperitoneal injection (0.01 mL/mg) of a mixture of Zoletil (30 mg/kg) and Rompun (10 mg/kg). Spleens were exteriorized via a small left abdominal flank incision. A single intrasplenic injection of 2×10^6 luciferase-labeled cells in 50 μL of Hank's balanced salt solution (HBSS) (Gibco) was administered with a 30-gauge needle. Gentle pressure was applied to the injection site with a cotton swab for one minute to staunch bleeding and to prevent leakage of tumor cells. Spleens were carefully reinserted into the abdominal cavity, and the wound was sutured using 6-0 black silk (10 mice per group). To examine the effect of SRC and FGFR4 inhibitors on migration and invasion of CRC cells *in vivo*, mice were randomized into 4 treatment groups of 10 mice each: vehicle, BLU-554, KX2-391, and BLU-554 plus KX2-391. Treatments were initiated 1 weeks after inoculation. BLU-554 (10 mg/kg per mouse) [2], KX2-391 (15 mg/kg per mouse) [3] were administered every day by oral gavage. Every week, the mice received intraperitoneal injections of 150 mg/kg of D-luciferin, and images were acquired 10 minutes after injection with an IVIS 100 Imaging System (Xenogen, Hopkinton, MA, USA). Each image was acquired within 2 minutes. The survival durations of the mice were monitored, and 9 weeks after tail vein and intrasplenic injections, all mice were sacrificed for further histological examination for lung and liver metastases.

Chromatin immunoprecipitation (ChIP) assays

1×10^7 CRC cells were used per ChIP analysis. Cells were cross-linked in 1% formaldehyde at 37 °C for 10 min. After washing with PBS, the cells were resuspended

in 300 μl of lysis buffer. The DNA was sheared to small fragments by sonication. Sonicated chromatin was diluted to a final SDS concentration of 0.1% and aliquots were rotated with antibody O/N at 4 °C. The recovered supernatants were incubated with specific antibodies or an isotype control IgG for 2 hr in the presence of herring sperm DNA and Protein A/G Magnetic beads (Thermo Fisher). These antibodies are anti-ELF4 (Santa Cruz, sc-390689), and anti-SP1 (CST, #9389). The immunoprecipitated DNA was retrieved from the beads with 1% SDS and a 1.1 M NaHCO3 solution at 65 °C for 6 hr. The DNA was then purified using a PCR Purification Kit (Qiagen, USA). The primers are shown in **Table S6**.

For ChIP assays of tissues, cells were first separated from six pairs of fresh frozen CRC tissues and normal colon tissues collected after surgical resection. In detail, surgically extracted tumor tissues were first washed by 1×cold, PBS, 5min, for three times and added to medium supplemented with antibiotic and antifungal agents. Use a clean razor blade to cut a pie of tissue (around $5mm^3$) into small piece (typical $1mm^3$ or smaller). Then, digestion the tissues with DNase I (20 mg/mL; Sigma-Aldrich) and collagenase (1.5 mg/mL; Sigma-Aldrich) and placed on table concentrator, 37°C, for 1h. At the end of the hour, we filtered the dissociated cells through 100-μm-pore filters rinsed with fresh media. The 1×red cell lysis was added to the tissues and incubated for 5 minutes to lysis the red blood cell, followed by another rinse. The dissociated cells were crosslinked using 1% formaldehyde for 10 minutes at 37°C. After cell lysis, the DNA was fragmented by sonication. Anti-ELF4 (Santa Cruz, sc-390689), and anti-SP1 (CST, #9389), or IgG (negative control) antibody was used to immunoprecipitated the

fragment DNA. Then, qRT-PCR was used to amplify the corresponding binding site on the promoters.

RNA-sequencing and bioinformatic analysis

Total RNA was extracted from negative control and ELF4 overexpression groups of SW480 cells using TRIzol reagent (Invitrogen), and each group was prepared with three parallel replicates. The quality control, library construction, and RNA sequencing were all performed in Beijing Genomic Institute (BGI). For data analysis, differential expression analysis of the two groups was performed using the DESeq2 R package (1.20.0). The cutoff value of differentially expressed genes (DEGs) was set as $|\log2[\text{fold change (FC)}]| > 1$ and $p < 0.05$. The heatmap of DEGs was drawn by the pheatmap R package. The volcano plot was performed using the ggplot2 R package. Gene Ontology (GO) and Kyoto Encyclopedia of Genes and Genomes (KEGG) pathways analyses for DEGs were performed using the clusterProfiler R package. The list of DEGs was provided in the **Data S1**. The results of GO and KEGG analyses were provided in the **Data S2**.

TCGA (The Cancer Genome Atlas) and GSE (Genomic Spatial Event) data analyses

CRC transcriptomic profiles were obtained from the TCGA, GEO (GSE41258), while colon tissues from Genotype-Tissue Expression (GTEx) were chose as controls.

Quantification and Statistical Analysis

The quantitative data were compared between groups using the Student's t-test. Categorical data were analyzed using the Fisher's exact test. The cumulative recurrence and survival rates were determined using the Kaplan-Meier method and log-rank test. The Cox proportional hazards model was used to determine the independent factors that influence survival and recurrence based on the variables that had been selected from the univariate analyses. The difference of tumor growth [measured by total flux (photon/sec)] between groups was analyzed by two-way ANOVA. A value of $p < 0.05$ was considered to be significant. All the analyses were performed using the SPSS software (version 19.0).

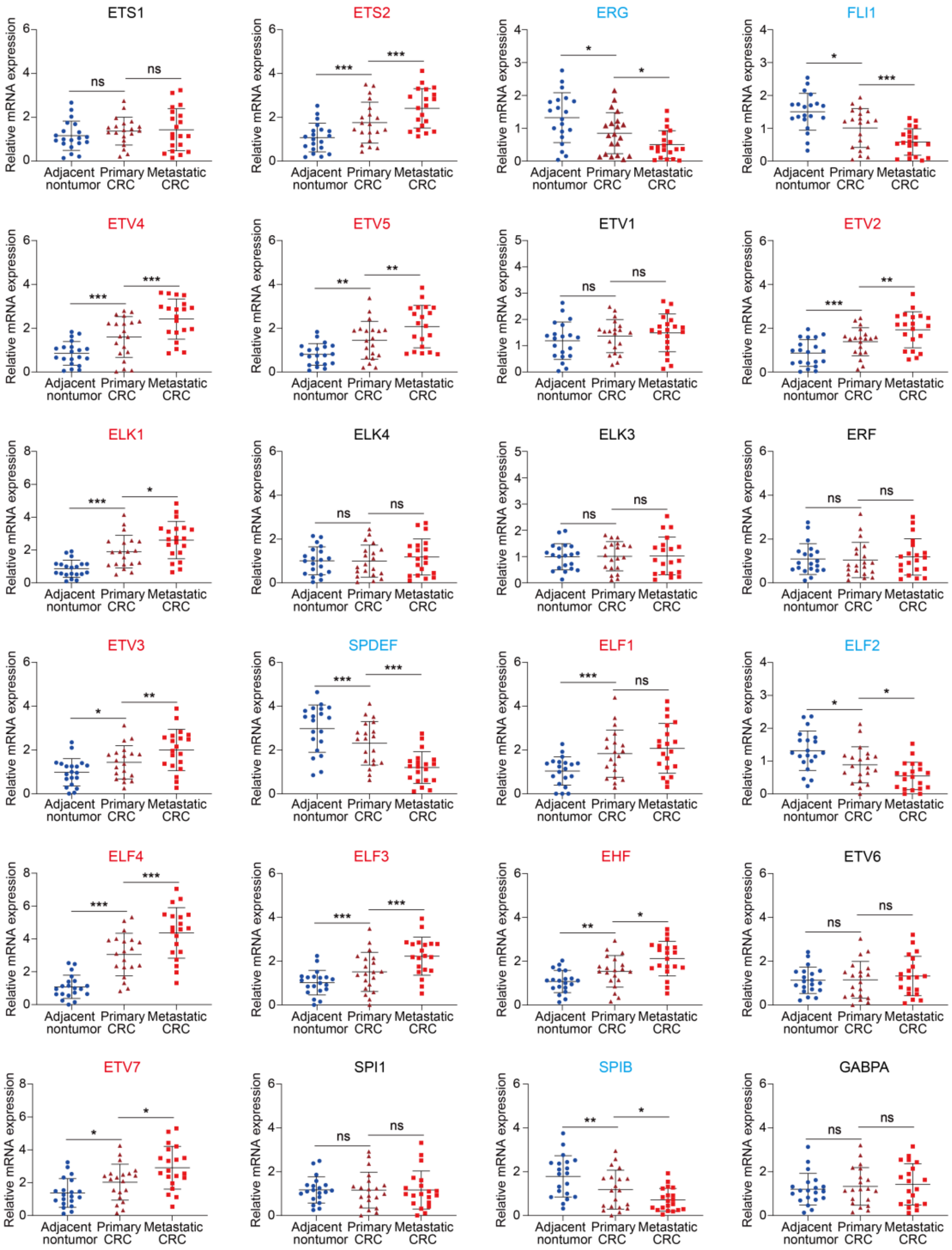

Supplementary Figure 1 RT-qPCR was used to analyze the expression of ETS family members in adjacent nontumorous (n = 20) and 20 pairs of primary and metastatic CRC tissues. * P<0.05, ** P<0.01, *** P<0.001.

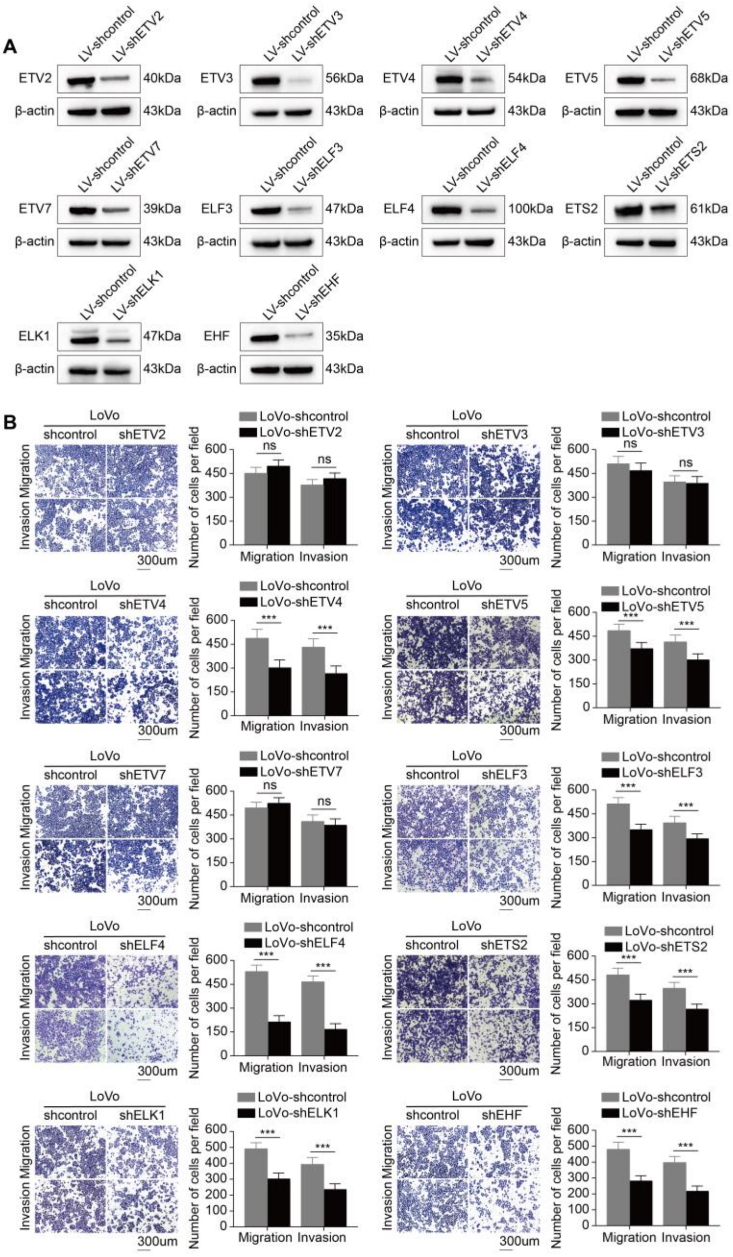

Supplementary Figure 2

(A) The protein level of ETV2, ETV3, ETV4, ETV5, ETV7, ELF3, ELF4, ETS2, ELK1 and EHF in LoVo cells transfected with corresponding lentivirus was detected by western blotting.

(B) The transwell assays were used to analyze the migrative and invasive ability after downregulating the expression of these genes in LoVo cells. * P<0.05, ** P<0.01, *** P<0.001.

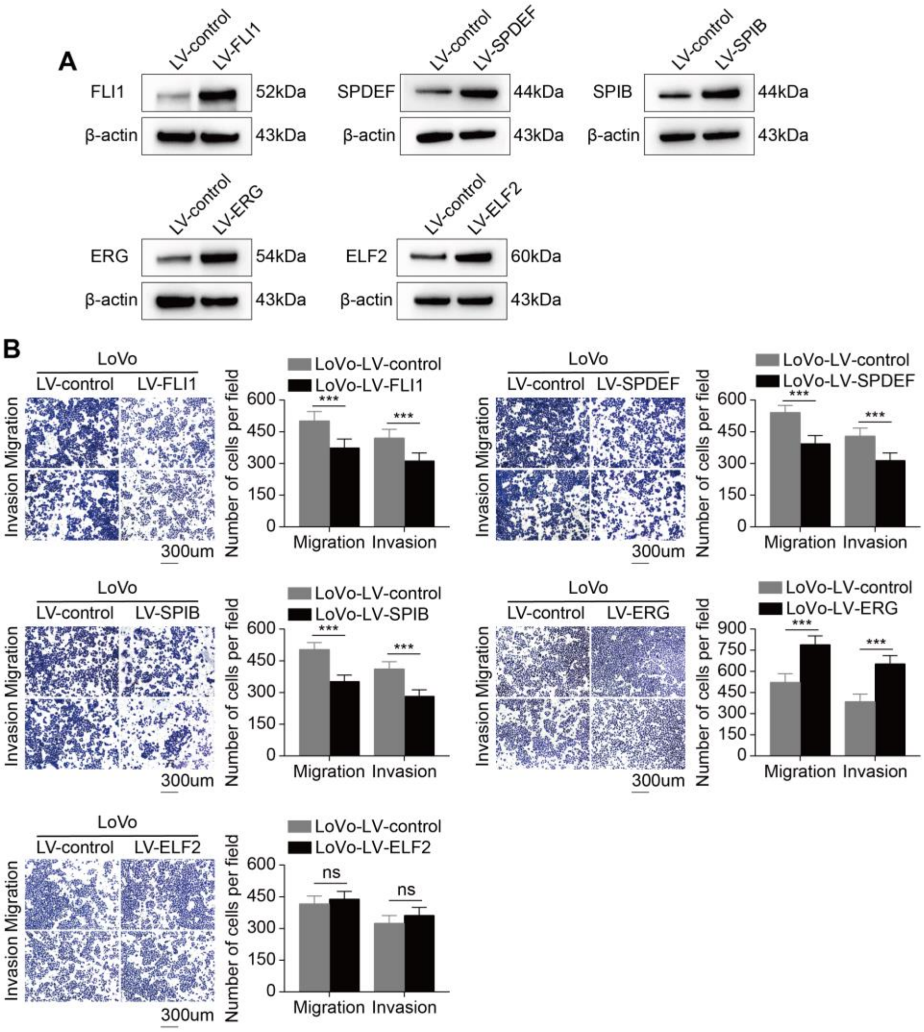

Supplementary Figure 3

(A) The protein level of FLI1, SPDEF, SPIB, ERG and ELF2 in LoVo cells transfected with corresponding lentivirus was detected by western blotting.

(B) The transwell assays were used to analyze the migrative and invasive ability after upregulating the expression of these genes in LoVo cells. * P<0.05, ** P<0.01, *** P<0.001.

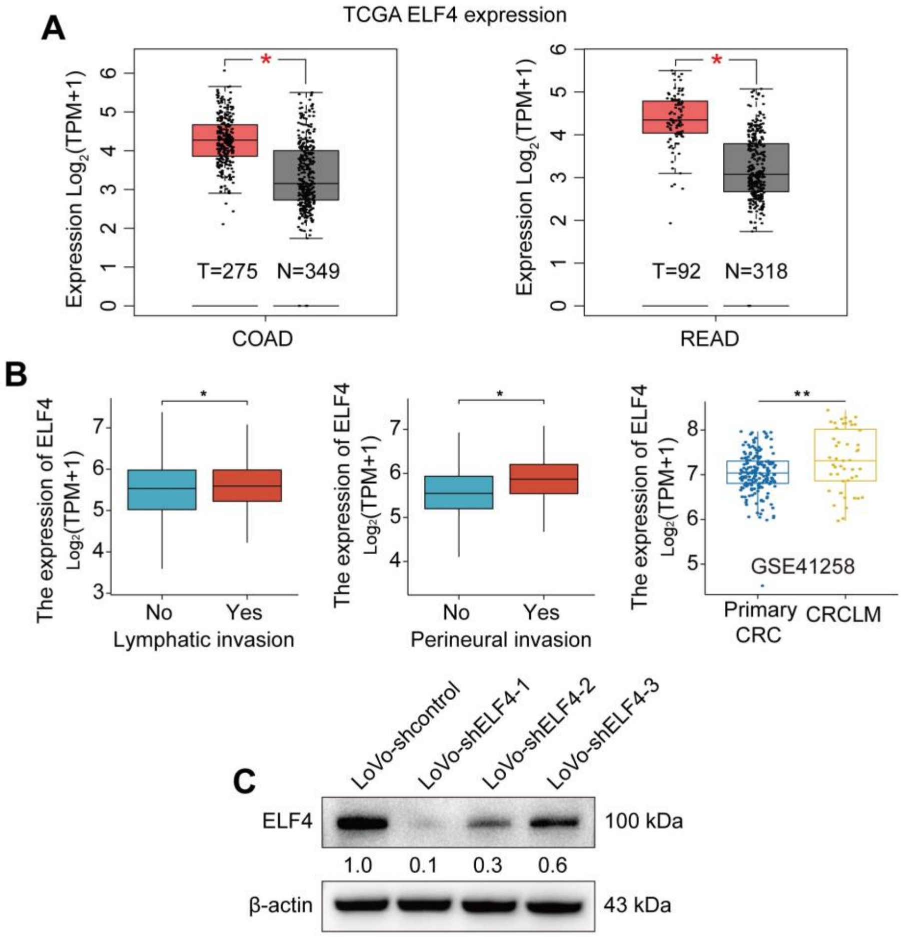

Supplementary Figure 4

(A) Left: The box-plot for differential expression of ELF4 in colon cancer and normal colon tissues (data was extracted from the TCGA-COAD dataset) was shown. The red box showed the expression level of ELF4 in tumor samples (n= 275), and the gray box exhibited the ELF4 expression in normal specimens (n=349). Right: The box-plot for differential expression of ELF4 in rectum cancer and normal tissues was shown by analyzing the TCGA-READ dataset. The red box showed the expression level of ELF4 in tumor samples (n= 92), and the gray box exhibited the ELF4 expression in normal specimens (n=318).

(B) Left: The mRNA expression of ELF4 in CRC patients with or without lymphatic

invasion (data from TCGA-COAD dataset). Middle: The mRNA expression of ELF4 in CRC patients with or without perineural invasion (data from TCGA-COAD dataset). Right: The mRNA expression of ELF4 in CRC patients with or without liver metastasis (data from the GSE41258 dataset).

(C) The protein level of ELF4 was detected by western blotting in the indicated cells.

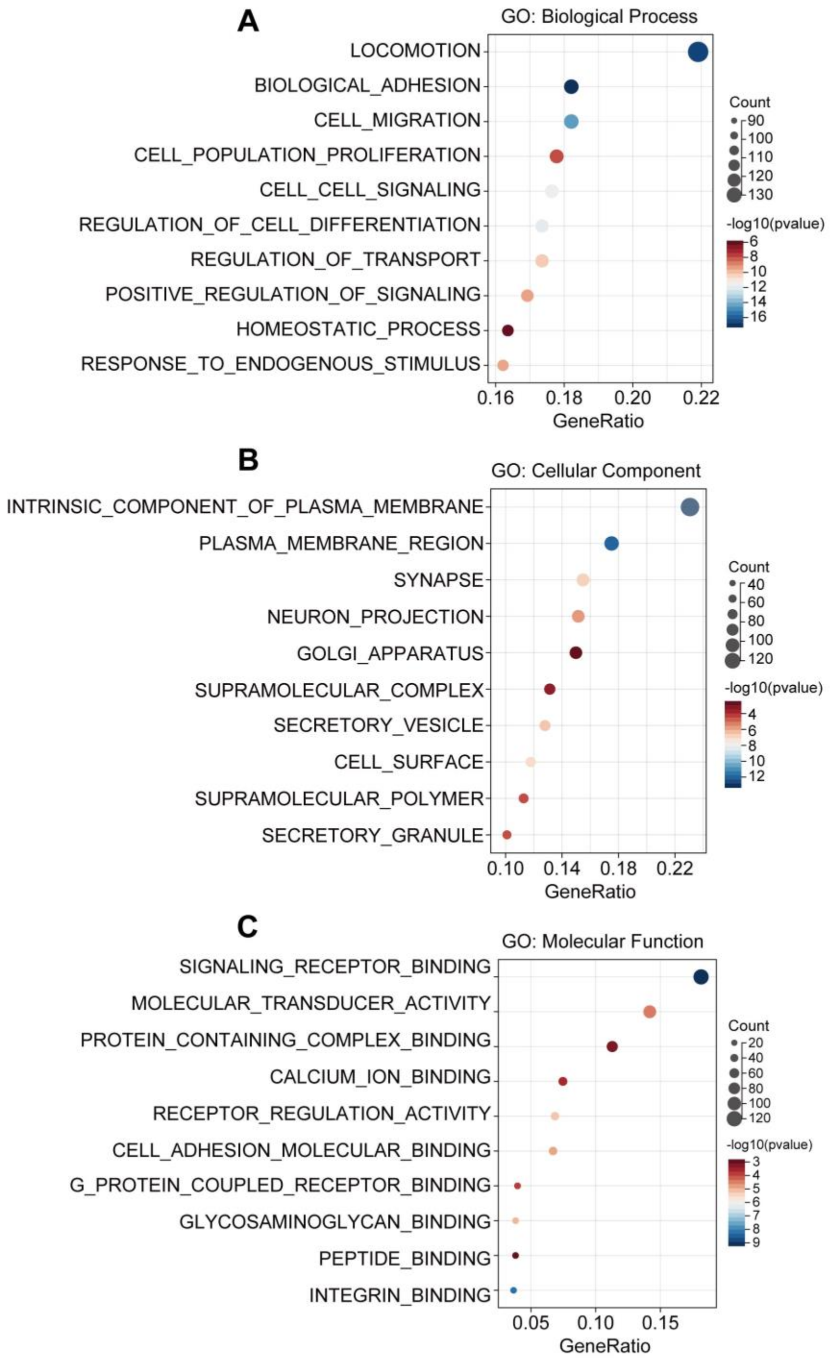

Supplementary Figure 5 Gene Ontology (GO) analyses of the differentially expressed genes between SW480-ELF4 and SW480-control cells were shown.

(A) The top 10 significantly enriched GO biological process terms.

(B) The top 10 significantly enriched GO cellular component terms.

(C) The top 10 significantly enriched GO molecular function terms.

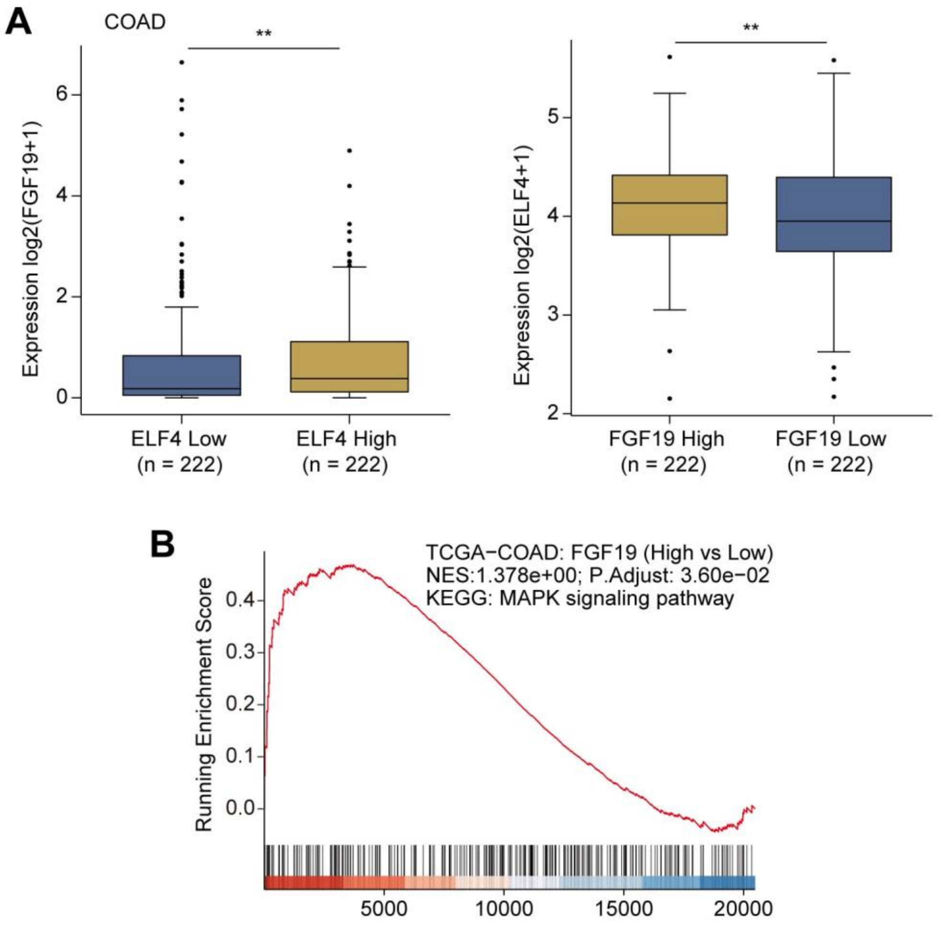

Supplementary Figure 6

(A) The correlation between ELF4 and FGF19 expression in COAD. Analyses were performed by CAMOIP website (http://www.camoip.net/).

(B) GSEA analysis revealed that high FGF19 expression in COAD was related to MAPK signaling pathway ($P<0.05$). Analyses were performed by CAMOIP website (http://www.camoip.net/).

Supplementary Table 1. Correlation between ELF4 expression and clinicopathological characteristics of CRCs in two independent cohorts of human CRC tissues

Clinicopathological variables		Cohort I (n = 334) Tumor ELF4 expression Negative (n = 154)	Positive (n =180)	p Value	Cohort II (n = 390) Tumor ELF4 expression Negative (n = 172)	Positive (n = 218)	p Value
Age		67.36(9.38)	65.02(12.56)	0.057	67.94(10.74)	67.13(12.17)	0.496
Sex	female	77	75		73	104	
	male	77	105	0.127	99	114	0.300
Tumor location	right colon	71	90		68	97	
	left colon	50	67		76	92	
	rectum	33	23	0.105	28	29	0.541
Tumor size	< 5cm	62	82		63	86	
	≥ 5cm	92	98	0.330	109	132	0.569
Tumor differentiation	well or moderate	131	95		127	97	
	poor	23	85	< 0.001	45	121	< 0.001
Tumor invasion	T1- T3	136	118		145	168	
	T4	18	62	< 0.001	27	50	0.075
Lymph node metastasis	absent	110	75		152	74	
	present	44	105	< 0.001	20	144	< 0.001
Distant metastasis	absent	149	120		165	153	
	present	5	60	< 0.001	7	65	< 0.001
AJCC stage	Stage I- II	110	69		151	68	
	Stage III- IV	44	111	< 0.001	21	150	< 0.001

Supplementary Table 2. Univariate and multivariate analysis of factors associated with survival and recurrence in two independent cohorts of human CRC tissues

	Time To Recurrence		Overall Survival	
Clinical Variables	HR (95% CI)	P value	HR (95% CI)	P value
**Cohort I (n = 334)**				
**Univariate analysis**				
Age	0.993(0.980-1.006)	0.280	0.993(0.980-1.006)	0.271
Sex (female versus male)	1.232(0.924-1.642)	0.156	1.160(0.866-1.552)	0.319
Tumor size (< 5 versus ≥ 5 cm)	0.797(0.594-1.069)	0.130	0.791(0.587-1.065)	0.123
Tumor differentiation (well/moderate versus poor)	0.141(0.104-0.192)	< 0.001	0.147(0.108-0.200)	< 0.001
Tumor invasion (T1-T3 versus T4)	0.346(0.255-0.467)	< 0.001	0.355(0.261-0.482)	< 0.001
Lymph node metastasis (absent versus present)	0.072(0.049-0.105)	< 0.001	0.075(0.051-0.110)	< 0.001
Distant metastasis (absent versus present)	0.110(0.078-0.155)	< 0.001	0.113(0.080-0.159)	< 0.001
AJCC stage (I-II versus III-IV)	0.068(0.046-0.101)	< 0.001	0.071(0.048-0.105)	< 0.001
ELF4 expression (negative versus positive)	0.327(0.239-0.449)	< 0.001	0.325(0.236-0.449)	< 0.001
**Multivariate analysis**				
Tumor differentiation (well/moderate versus poor)	0.829 (0.563-1.221)	0.342	0.880(0.593-1.305)	0.524
Tumor invasion (T1-T3 versus T4)	0.746(0.534-1.042)	0.085	0.769(0.546-1.083)	0.133
Lymph node metastasis (absent versus present)	0.333(0.130-0.853)	0.022	0.334(0.128-0.870)	0.025
Distant metastasis (absent versus present)	0.445(0.301-0.658)	< 0.001	0.436(0.294-0.648)	< 0.001
AJCC stage (I-II versus III-IV)	0.286(0.106-0.768)	0.013	0.294(0.107-0.809)	0.018
ELF4 expression (negative versus positive)	0.634(0.447-0.900)	0.011	0.676(0.470-0.973)	0.035
**Cohort II (n = 390)**				
**Univariate analysis**				
Age	0.998(0.988-1.009)	0.774	1.000(0.989-1.011)	0.951
Sex (female versus male)	1.070(0.847-1.351)	0.569	1.116(0.880-1.415)	0.367
Tumor size (< 5 versus ≥ 5 cm)	0.901(0.709-1.146)	0.396	0.877(0.686-1.122)	0.297
Tumor differentiation (well/moderate versus poor)	0.469(0.370-0.593)	< 0.001	0.449(0.353-0.571)	< 0.001
Tumor invasion (T1-T3 versus T4)	0.605(0.461-0.796)	< 0.001	0.607(0.459-0.803)	< 0.001
Lymph node metastasis (absent versus present)	0.193(0.150-0.248)	< 0.001	0.172(0.132-0.222)	< 0.001
Distant metastasis (absent versus present)	0.130(0.095-0.178)	< 0.001	0.111(0.081-0.154)	< 0.001
AJCC stage (I-II versus III-IV)	0.179(0.138-0.230)	< 0.001	0.159(0.122-0.207)	< 0.001
ELF4 expression (negative versus positive)	0.382(0.299-0.489)	< 0.001	0.355(0.275-0.457)	< 0.001

**Multivariate analysis**				
Tumor differentiation (well/moderate versus poor)	0.828(0.636-1.080)	0.164	0.839(0.639-1.101)	0.205
Tumor invasion (T1-T3 versus T4)	0.698(0.526-0.928)	0.013	0.710(0.531-0.949)	0.020
Lymph node metastasis (absent versus present)	1.402(0.653-3.014)	0.386	1.175(0.546-2.526)	0.680
Distant metastasis (absent versus present)	0.364(0.257-0.514)	< 0.001	0.319(0.224-0.454)	< 0.001
AJCC stage (I-II versus III-IV)	0.189(0.085-0.423)	< 0.001	0.204(0.091-0.456)	< 0.001
ELF4 expression (negative versus positive)	0.710(0.536-0.941)	0.017	0.679(0.509-0.907)	0.009

Supplementary Table 3. Correlation between FGFR4 expression and clinicopathological characteristics of CRCs in two independent cohorts of human CRC tissues

		Cohort I (n = 334)			Cohort II (n = 390)		
		Tumor FGFR4 expression			Tumor FGFR4 expression		
		Negative	Positive		Negative	Positive	
Clinicopathological variables		(n = 174)	(n = 160)	p Value	(n = 200)	(n = 190)	p Value
Age		66.68(11.03)	65.46(11.49)	0.322	67.33(11.65)	67.66(11.48)	0.776
Sex	female	82	70		94	83	
	male	92	90	0.536	106	107	0.511
Tumor location	right colon	88	73		78	87	
	left colon	50	67		91	77	
	rectum	36	20	0.020	31	26	0.398
Tumor size	＜ 5cm	77	67		78	71	
	⩾ 5cm	97	93	0.661	122	119	0.74
Tumor differentiation	well or moderate	137	89		131	93	
	poor	37	71	< 0.001	69	97	0.001
Tumor invasion	T1-T3	138	116		164	149	
	T4	36	44	0.145	36	41	0.375
Lymph node metastasis	absent	116	69		139	87	
	present	58	91	< 0.001	61	103	< 0.001
Distant metastasis	absent	157	112		178	140	
	present	17	48	< 0.001	22	50	< 0.001
AJCC stage	Stage I- II	113	66		137	82	
	Stage III- IV	61	94	< 0.001	63	108	< 0.001

Supplementary Table 4. Correlation between SRC expression and clinicopathological characteristics of CRCs in two independent cohorts of human CRC tissues

Clinicopathological variables		Cohort I (n = 334) Tumor SRC expression Negative (n = 116)	Cohort I (n = 334) Tumor SRC expression Positive (n = 218)	p Value	Cohort II (n = 390) Tumor SRC expression Negative (n = 129)	Cohort II (n = 390) Tumor SRC expression Positive (n = 261)	p Value
Age		67.12(11.34)	65.56(11.20)	0.227	66.35(11.16)	68.05(11.72)	0.172
Sex	female	53	99		58	119	
	male	63	119	0.961	71	142	0.906
Tumor location	right colon	56	105		49	116	
	left colon	39	78		55	113	
	rectum	21	35	0.865	25	32	0.146
Tumor size	＜ 5cm	53	91		47	102	
	⩾ 5cm	63	127	0.488	82	159	0.613
Tumor differentiation	well or moderate	97	129		95	129	
	poor	19	89	< 0.001	34	132	< 0.001
Tumor invasion	T1-T3	104	150		105	208	
	T4	12	68	< 0.001	24	53	0.691
Lymph node metastasis	absent	83	102		103	123	
	present	33	116	< 0.001	26	138	< 0.001
Distant metastasis	absent	110	159		123	195	
	present	6	59	< 0.001	6	66	< 0.001
AJCC stage	Stage I- II	83	96		102	117	
	Stage III- IV	33	122	< 0.001	27	144	< 0.001

Supplementary Table 5. Correlation between FGF19 expression and clinicopathological characteristics of CRCs in two independent cohorts of human CRC tissues

Clinicopathological variables		Cohort I (n = 334) Tumor FGF19 expression Negative (n = 217)	Positive (n = 117)	p Value	Cohort II (n = 390) Tumor FGF19 expression Negative (n = 257)	Positive (n = 133)	p Value
Age		66.12(11.42)	66.05(10.99)	0.955	67.84(11.19)	66.80(12.24)	0.397
Sex	female	96	56		114	63	
	male	121	61	0.526	143	70	0.571
Tumor location	right colon	114	47		96	69	
	left colon	53	64		122	46	
	rectum	50	6	< 0.001	39	18	0.019
Tumor size	＜ 5cm	92	52		105	44	
	≥ 5cm	125	65	0.718	152	89	0.134
Tumor differentiation	well or moderate	165	61		174	50	
	poor	52	56	< 0.001	83	83	< 0.001
Tumor invasion	T1-T3	173	81		211	102	
	T4	44	36	0.032	46	31	0.203
Lymph node metastasis	absent	142	43		173	53	
	present	75	74	< 0.001	84	80	< 0.001
Distant metastasis	absent	191	78		225	93	
	present	26	39	< 0.001	32	40	< 0.001
AJCC stage	Stage I- II	136	43		169	50	
	Stage III- IV	81	74	< 0.001	88	83	< 0.001

Supplementary Table 6. Primer sequences used in the study

Primer name	Primer sequences	Enzyme
Primers for real-time PCR:		
ELF4 sense:	5'- CATCATAACAGACGGGACCTTG -3'	
ELF4 antisense:	5'- GCTGGGAGACTCCATATTGAGTA -3'	
FGFR4 sense:	5’-GAGGGGCCGCCTAGAGATT-3’	
FGFR4 antisense:	5’-CAGGACGATCATGGAGCCT-3’	
SRC sense:	5’- GAGCGGCTCCAGATTGTCAA-3’	
SRC antisense:	5’- CTGGGGATGTAGCCTGTCTGT-3’	
β-actin sense:	5’- CTCCATCCTGGCCTCGCTGT -3’	
β-actin antisense:	5’- GCTGTCACCTTCACCGTTCC -3’	
Primers for FGFR4 promoter construct:		
(-1722/+106) FGFR4 sense:	5’-TATAGAGCTCGGCCTTACAGTTATGCAA-3’	SacI
(-1087/+106) FGFR4 sense:	5’-TATAGAGCTCCCGTCATTGTGCCCGGCCAT -3’	SacI
(-517/+106) FGFR4 sense:	5’-TATAGAGCTCAAGTATCACTCCGCAGGT -3’	SacI
(-199/+106) FGFR4 sense:	5’-TATAGAGCTCCACCCCTGGCCACACCT -3’	SacI
Antisense:	5’-ATATGCTAGCACCTGGCTCCTCCGCCGCCTC-3’	Nhel
Primers for FGFR4 promoter site-directed mutagenesis:		
ELF4 binding site:		
binding site 4 mutation sense:	5’-GGGGACAACCAaagtCTGAGCCTCAG-3’	
binding site 4 mutation antisense:	5’-CTGAGGCTCAGacttTGGTTGTCCCC-3’	
binding site 3 mutation sense:	5’-GCTGCCTCGACccttTGGCTCAAGCA-3’	
binding site 3 mutation antisense:	5’-TGCTTGAGCCAaaggGTCGAGGCAGC-3’	
binding site 2 mutation sense:	5’-GGGCCATTCCAaaggGAGAGAATGGT-3’	
binding site 2 mutation antisense:	5’-ACCATTCTCTCccttTGGAATGGCCC-3’	
binding site 1 mutation sense:	5’-GGCGGGGTCCCaaagGTGGGCTGACT-3’	
binding site 1 mutation antisense:	5’-AGTCAGCCCACctttGGGACCCCGCC-3’	
Primers used for ChIP in the FGFR4 promoter:		
distant region sense:	5’- TCAGGAGACAAGGAGGTTGC -3’	
distant region antisense:	5’- GGCTTTCATTCACCTTCCCG -3’	
binding site 4/3 sense:	5’- GACAGGGTCTACACTAGCT -3’	
binding site 4/3 antisense:	5’- GCCAGAGGATTGCTTGAG -3’	
binding site 2 sense:	5’- AAGGAAGTGAGCTGAGAATA -3’	
binding site 2 antisense:	5’- ATACATAGATTGCCATGACC -3’	
binding site 1 sense:	5’-CAGGCATTCCCTGAAGGGTG-3’	
binding site 1 antisense:	5’-ACCCAGTCAGCCCACTCC-3’	
Primers for SRC promoter construct:		
(-1481/+117) SRC sense:	5’-TATAGAGCTCGACTATACAACCAGCACTCA-3’	SacI
(-1102/+117) SRC sense:	5’- TATAGAGCTCGAACTTGAACTCTTGTCTGTAG-3’	SacI
(-896/+117) SRC sense:	5’- TATAGAGCTCGAAGAACAAGGCGAGGTAG-3’	SacI
(-518/+117) SRC sense:	5’- TATAGAGCTCCTGAGGCTTCTTACATTATCTG-3’	SacI
Antisense:	5’- ATATGCTAGCAGGTTGTGCTAGATGAATGG-3’	Nhel
Primers for SRC promoter site-directed mutagenesis:		
ELF4 binding site:		

binding site 4 mutation sense:	5’-GATGATTTGCAaaggTTAAGCAATGA-3’	
binding site 4 mutation antisense:	5’-TCATTGCTTAAccttTGCAAATCATC-3’	
binding site 3 mutation sense:	5’-TGCACAATCCTaaggCTTGCCAGGGT-3’	
binding site 3 mutation antisense:	5’-ACCCTGGCAAGccttAGGATTGTGCA-3’	
binding site 2 mutation sense:	5’-AGGGCGTGTCAaaggGCATGTTGTGG-3’	
binding site 2 mutation antisense:	5’-CCACAACATGCccttTGACACGCCCT-3’	
binding site 1 mutation sense:	5’-GCCCTTGAAGTtaatAGTGGCTTCAG-3’	
binding site 1 mutation antisense:	5’-CTGAAGCCACTattaACTTCAAGGGC-3’	
Primers used for ChIP in the SRC promoter:		
distant region sense:	5’- GTAGAGACACACTTGAGCCT -3’	
distant region antisense:	5’- CCTTCAAACGGCATTAACCAC -3’	
binding site 4/3 sense:	5’- GCTGTCCATTAACGATGATT-3’	
binding site 4/3 antisense:	5’- GGCAAGTTCCAGGATTGT-3’	
binding site 2 sense:	5’- CACACTCAGAACGGACTTA-3’	
binding site 2 antisense:	5’- CCTCGCCTTGTTCTTCAA-3’	
binding site 1 sense:	5’- ACGCTCAATAATGGAACCA-3’	
binding site 1 antisense:	5’- GAGAGGAGGCAACTTAGAC -3’	
Primers for ELF4 promoter construct:		
(-1998/+103) ELF4 sense:	5’-TATAGCTAGCTCATTCAGTCCTTGCCAC-3’	NheI
(-1090/+103) ELF4 sense:	5’-TATAGCTAGCAGATACCCCATTTACCCT-3’	NheI
(-474/+103) ELF4 sense:	5’-TATAGCTAGCCGTGTCTGTCCATTCG-3’	NheI
(-156/+103) ELF4 sense:	5’-TATAGCTAGCCGCCTGAGATGATCCTT-3’	NheI
antisense:	5’-ATATCTCGAGGCCTCAACATCAATACCG-3’	XhoI
Primers for ELF4 promoter site-directed mutagenesis:		
ELK1 binding site:		
binding site mutation sense:	5’-TTAGGGACATCtaagACCTACCCCAA-3’	
binding site mutation antisense:	5’-TTGGGGTAGGTcttaGATGTCCCTAA-3’	
SP1 binding site:		
binding site mutation sense:	5’-AGGTCTCAGGGattaGCGCCCCAGTC-3’	
binding site mutation antisense:	5’-GACTGGGGCGCtaatCCCTGAGACCT-3’	
ETS1 binding site:		
binding site mutation sense:	5’-GTCCAGCCACAaagcCTTTCCTTGGG-3’	
binding site mutation antisense:	5’-CCCAAGGAAAGgcttTGTGGCTGGAC-3’	
Primers used for ChIP in the ELF4 promoter:		
distant region sense:	5’- GCTCATGTTTGTAATCCCGACAC-3’	
distant region antisense:	5’- GGGACTACAGATGCACACCA -3’	
binding site 3/2 sense:	5’- ATCTCATTCAGTCCTTGCCAC-3’	
binding site 3/2 antisense:	5’- TTACAGGTGTGAGCCACCG-3’	
binding site 1 sense:	5’- TCTGAGTATCCAGGAGGTC-3’	
binding site 1 antisense:	5’- GAGTGGTGGTGGATGATG-3’	

Supplementary Table 7. Knockdown shRNA sequences used in this study

Gene name	Sequence
ELF4	
shRNA-1	CCGGCCCTGATTTACTGCATCTGTACTCGAGTACAGATGCAGTAAATCAGGGTTTTTG
shRNA-2	CCGGCGCGGAAGTCTTACTCAATATCTCGAGATATTGAGTAAGACTTCCGCGTTTTTG
shRNA-3	CCGGGCACTAAGATACTACTACCAACTCGAGTTGGTAGTAGTATCTTAGTGCTTTTTG
FGFR4	
shRNA-1	CCGGCCCTCGAATAGGCACAGTTACCTCGAGGTAACTGTGCCTATTCGAGGGTTTTTG
shRNA-2	CCGGCCTATGTGCAAGTCCTAAAGACTCGAGTCTTTAGGACTTGCACATAGGTTTTTG
shRNA-3	CCGGGCCGACACAAGAACATCATCACTCGAGTGATGATGTTCTTGTGTCGGCTTTTTG
SRC	
shRNA-1	CCGGGACAGACCTGTCCTTCAAGAACTCGAGTTCTTGAAGGACAGGTCTGTCTTTTTG
shRNA-2	CCGGGTCATGAAGAAGCTGAGGCATCTCGAGATGCCTCAGCTTCTTCATGACTTTTTG
shRNA-3	CCGGTGCGACGATGACTGGGACTTTCTCGAGAAAGTCCCAGTCATCGTCGCATTTTTG

Supplementary Table 8

ELF4 Promoter (-2200 ~ +200)

>NC_000023.11:c130113927-130111728 Homo sapiens chromosome X, GRCh38.p14 Primary Assembly

-2200 AGGCTTCAGCCTTGGTTCAAACTACATTCACTTACGGGAGGGATCCACCATTAGTCCTGAGCAACTTCAGCCCTGGCCCT

-2120 GTCATACTTTCTCCTGAGAGCTGGGTTGAGAAAGGGAAATGAAGATGAACATGACACCTAAGAGAGCTAACATTTATTGA

-2040 AGACTTACTGGGCACCAGACACTGCCATAGACATTTGTTATCTCATTCAGTCCTTGCCACAGCCTTGGGAGGTAGGTTCAT

-1959 ATTGTCTCCAATTTAAAAATAAGAAAAGTGAGGCTGGGGGCAGGGAAGTGCAGATGGTTAATGGGTACAAAAATATAGTTA

SP1 binding site 3

-1878 GAATGAGTAAGATCTAGTACCTGATAACACTACAGGGTGCCTGCAGTCAACAATAATTTATTGCACATTTAAAAATAACTA

AP-1 binding site 3

-1797 GGGCCAGGCGCGGTGGCTCACACCTGTAATCCCAGCACTTTGGGAAGCCAAGGCAGGCGCATCACCTGAGGTCAGGAGTT

SP1 binding site 2

-1717 TGAGACCAGCCTGACCAACATGGAGAAACCCCATCTCTACTAAAAATACAAAAATTAGCCAGGTGTGGTGGCGTATGCCTA

-1636 TAATCCCAGTTACTCGGGAGGCTGAGGCAGGAGAATCGCTTGAACCCAGGAGGCAGAGGTTGCGGTGAGCCGAGATCAC

-1557 ACCATTGCACTCCAGCCTGGGCAAGAAGAGTGAAATTCCGTCTCAAAAAAAAAAAAAATCTAAACTAAAAGAGTATAACTG

-1477 GATTGTATATAACACAAAGAAAGAGGCCGGGCGCAGTGGCTCACGCCTGTAATCCCAGCACTTTAGGAGGCCGAGGCGGG

-1397 TGGATGACGAGGTCAGGAGATCGAGACCATCCTGACTAACACAGTGAACCCCTGCCTCTACTAAAAAATACAAAAAATTA

-1317 GCCGGGCATGGTGGCGGGTTCCTGTAGTCCCAGCTACTCAGGAGGCTGAGGCAGGAGAATGGCATGAACCCGGGAGGTG

-1238 GAGCTTGCAGTGAGCCGAGATCGCGCCACTGCACTCCAGTCTGGGAGAAAGAGCGAGACTCCATCTCAAAAAAAAAAAA

-1159 AAAAAAAAAGAAGGAAAAAAAAAAAAAGAAAAAGAAAACACACAAAGAAAGGAGAAATGCTTGAGGTGACAGATACC

-1083 CCATTTACCCTGATGTGATTATTACACATTGCATGCCTGTATCAAAACATCTCATGTAACCCATAAATACACACACCTACAA

-1001 TGTACCCATAAAAATAAAAAACAAAAATAAAAAAATTAATTTATTTAATTTATTTATTTTTTTGAGATGGAGTCTCACTGTG

-919 TCATCCAGGCTGGAGTGCAGTGGCACGATCTCGGCTCACTGCAACCTCTGCCTCCCAGGTTCAAGTGACTCTCCTGCCTCA

AP-1 binding site 2

-838 GCCTCCCGAGTAGCTGGAACTACAGGCATGCACCACCACACCGGCTAATTTTTGTATTTTTAGTAGAGATGGGGTTTCACCA

NF-κB binding site 1

-756 TGTTGGCCAGGCTGGTCTTGAACTCCTGACCTCAAGTGATCCACCCCGCTCGGCATCCCAAAGTGCTGGGATTACAGGCAT

-675 GAGTCACCACACCCAGCCTAGTTTTTGTTTTTGTTCTGTTTTAAAGAAGAAAAGTGAGGCTCAGAGAGTTTAAGTGACTT

AP-1 binding site 1

-595 GCCTGAAGTCACACAGCTGGTAAATAGTGAGCTGGAATAGGAACCCAGGTCTGTCTGAGGTTACAGACCACTGAGCTCTT

ETS1 and ELK1binding site

-515 AGCAAAATGGGAAAGTGTGTGTGTGTGCACGCGTGTGCAGACGTGTCTGTCCATTCGTGAATTTCAAAGTCAAGGTAAAG

-435 AGGGCTTAGGGACATCCGGAACCTACCCCAAATTTGAGAGGGGACCTCGTGGATGAGAGGCCAGCAGAGAACTGTGTGTC

ELK1 binding site 1

-355 TCCAGGATCTGGAATCCCCTGGGAAAAGCATGCCCGCTTGGAAAAGACAAAGTGCTTGTTTTGGGGACTTGATTCTGAGT

-275 ATCCAGGAGGTCTCAGGGGCCGGCGCCCCAGTCCAGCCACAGGATCTTTCCTTGGGAACTTGTAATCTGCAGCTGCCTTTG

SP1 binding site 1 **ETS1 binding site 1**

-194 TGGCCATCATCCACCACCACTCCTGTCCCCCAGTCCTGCGCCTGAGATGATCCTTTCCCAAGGCAGGAGAAGCAGGCCTTT

-33 CTGCCAAGGCTTAGGCCTTGCTGCCCCCACCCCCAAAAAGGCCACGTGGAACTATACAGAGTAACAATTCCCAGCGCACT

Transcription starting site (nucleotide +1)

+48 CTCTCCAGAGCCTCCTATCAGCAGACCCACGTAGGCGGCGGTATTGATGTTGAGGCAGGTCAGAGAGGGCAAAGGGGAGA

+128 TAGAAGGGGAGCGTGCCTGAGTGGCTTTCAAGTGGGCGCGTGGAGCAGAGCAGCCTGGCTTTGGACAGGGGAA

(a) Transcription factors binding sites within the promoter regions of *ELF4*. The arrow represents the transcription starting site.

FGFR4 Promoter (-1999 ~ +201)

>NC_000005.10:177084916-177087115 Homo sapiens chromosome 5, GRCh38.p14 Primary Assembly

-1999 CCCACCTAAGCCTCCCAAAGTGCTGAGATTACAGGCGTGAGCCACCGTGCCCAGCCTAATATATATTTTTAATAGAGACG

-1919 AGATCTCACTATGTTGCCCAGGCTGGCCTCGAACTCCTGAGTTCAAGCGATCCTCCCGATTCAGCCTCCCACAGTGCTGG

-1839 GATTATAGGCATGAGCCACCATGCCTGGCCTCAATTATTTTCTGATTTATCTTTGGGAGGAGACCATTCTTTCGCTTAAGAA

-1757 GAAAAAGAAAAAAACAATTTTAAGTCTCTTTAATGGGCCTTACAGTTATGCAAAGATATGCCACTATCCCTTTGGGAGTGA

-1676 GGGTGGAAAGAACTAGACTTGCCTGCGTAACAATGAGAAAGGAAGTTGCTTCTTAAGACAGGGTCTACACTAGCTAGAG

-1597 GGGACAACCAGGACCTGAGCCTCAGACAGGGTCTCGCTCCATAGCCTGTTGCCCAGGCTACAGTGCAGTGGAGCGATCA

ELF4 binding site 4

-1518 CGGCTCACTGCTGCCTCGACTTCCTGGGCTCAAGCAATCCTCTGGCCTCAGCCTCCCAAGTAGCTGGGACCACAGGTGCA

ELF4 binding site 3

-1438 CCCAGCCCATTTCCCTTCCTCTCAAGGTGCCTTAAGAGAAGGAGAAGGCTTGAGATTCAGGTGAGGGATGGGCAGAGATC

-1358 AGAGGCTGCCTGGCCTCCTGGTTTGGCCACAGTCTAGATGCTCAGGAGAGAAGTGCTTACAGGTGAGGCAAACATGAGCC

-1278 AGGTGGGCACGGAGCTATCTGGGAGCACCTACTGTATGCCAGACACTTGAACTCTAATGGTGAGGTCTGAGGACACAGTT

-1198 CCTCTACTGAAAAATAGAGACAGGGCAACTATGTTGCCAGGGCTGGCCTCACACTCCTGAATTCAAGCTGTCCTCCTACC

-1118 TCAGTCTCCTAGTAACTAGAACTACAGGCACCCGTCATTGTGCCCGGCCATTCCACAGCTATTTACATAATTAACTGTTCAG

-1036 TTACAATTCTGGCAAGCGTGCTGAAGAAATAACAGGTCCTAAATAATTACAGCTAGTATTACAAAGCAAGCGCTGCATGCC

-955 AGGCTTACACATACTGCTAACATGTCTACTTTTTGCAACAACCTCTTGTGGGTGAGACTATCATTTGCCTGTTTTACTGAAA

-873 GGACAGTGAAGACTGAGGCCTTGTGTGATGGTGCTGAGAGCTGGGCCTATGACGTCCAGCCCTGTAGTCTGTGCTCTGAA

-793 CCACTTCACCCTGTTTCTTTCTCCATCCTCACAGTAGAGACGTCATCTAAGAGGAGCTTGCAAGTCCAGAGGGCTTCCTGA

-712 AGGAAGTGAGCTGAGAATAATATGAAATTAACCAGACCAAGGGCCATTCCAGGAAGAGAGAATGGTACGTGAAACTGCC

ELF4 binding site 2

-633 CAGGAGTAGCAGGGTGGGAGCCAGGAGGCGTGGGTCATGGCAATCTATGTATAAAGAGCTATGGGAATCTATAGGAAAAT

-553 TTAAGGGAGTGGCTGACACAGTCAGATTTGTGTTGTAAGTATCACTCCGCAGGTTGCGCAGGGAACAAATGAGGGAGCAG

-473 AAGGAAGGGGTTCTCCTATCCGCTGCACGGCACTGCGCAGAAGACAGGGGAGCCAGGCATTCCCTGAAGGGTGAAAAGC

-394 AAGGAGTAGAGCTGGGTAGTAGACTAGAATTTAGGAGCCTGGCCTGGGGCCTGGGTGGGGCGAAAGAGGCGGAGCCTGA

-315 ATGGGGTGTGTATAGGGGGGTTGCGTGTAGGGGTGTGTGTATAGGCTGGGGCGGGGTCCCGGGAGTGGGCTGACTGGGTC

ELF4 binding site 1

-235 GGGGGCGGGGCTCTCCAGGTGGGCGGGGATCTTGGCCACCCCTGGCCACACCTCTCTCCGGCTCGAGCTGGTCTAGGCGG

-155 GGCGGGCCCGAGGGGGTGTGGCAGGAGGTGGGCGGGCCCGGGTGGGGGGGGGGGGGCGTGGAAGGAGGGGCGGGCC

-78 CGAGCAGGAGGGGGCGGGCCCGAGGGGCGGGGTGGGACAGGAGGTGGGCCGCTCGCGGCCACGCCGCCGTCGCGGGTA

+1 CATTCCTCGCTCCCGGCCGAGGAGCGCTCGGGCTGTCTGCGGACCCTGCCGCGTGCAGGGGTCGCGGCCGGCTGGAGCTG

Transcription starting site (nucleotide +1)

+81 GGAGTGAGGCGGCGGAGGAGCCAGGTGAGGAGGAGCCAGGTGAGCAGGACCCTGTGCTGGGCGCGGAGTCACGCAGGC

+159 TCGAGGTGAGCCGGAACCCTTGTGGGCCCGGGCTGCGCTCCCA

(b) ELF4 binding sites within the promoter regions of *FGFR4*. The sequences highlighted in color represent the four binding sites of ELF4 on the FGFR4 promoter, and the arrow represents the transcription initiation site.

SRC Promoter (-2000 ~ +200)

>NC_000020.11:37342699-37344898 Homo sapiens chromosome 20, GRCh38.p14 Primary Assembly

-2000 GGAGGGACAGCAGGAGGCAGGTAGAGGAGGGGAGGAGACCAGGGCCTGCACAGCACGACATCTCTGTGTAAACTGTG

-1923 AAGTGCAGTGCCAGGGGAGGTGCCAAGCCCATTCCACAGACTCAGGCTAGAGCCCATCCCCCACCCACCCTGTCCTGTA

-1844 GCCCTGAGCCTGGTGGCTTCAACTAGACCACTCACAGCACACCATGTGGAGAATGGCAGAACTGTTCTGGAGTTTCCCAC

-1764 TGAGGCCTGTGATGGGTCCAGTTTCCTGGCTGCTGCAGCAAGGGTGAGCTGGGCTGGGGCTGGGCATAGCAGATCAGCCA

-1684 TCATTGAGCCAGGGTCACCAGGGCACTAGGTGTGCTGTGGGCAGCACCTCTGATCCCATGTCATGGGACAACTCTGGCTC

-1604 GTGGGTTCACTGCCAGTCGTGGAGGGCCAGGCCTCACCCTACCCCCTCCACCCTGCCCTCAGTTGTGGCTTTGGCCCACC

-1524 CAGGTTGAGGGAAGGAGGGAAGAGGAGGTTTCTGGAGACAGAGGACTATACAACCAGCACTCACCATCTCAGCAATGTT

-1445 GACGTCAATAGCATCTATCATCTGCTGGGTGCTTTTGGGTTGGGAAGTGGTTCCTGTTGGTGTTCTAAGACTGGCCTGGGG

-1364 CCATATATACAGTTGGTAGTCAATAAATGCTTGCTGTCCATTAACGATGATTTGCAGGAATTAAGCAATGAGTTGCTGCTTTA
ELF4 binding site 4

-1281 CCAACAAAGAATGTTTTAAGCAAAATAATTTGCACAAAGAGTCACTTATCTACCAGGCCAGACCAGCAACTCCCAACTGC

-1201 ACAATCCTGGAACTTGCCAGGGTTGTTACCCTCGTTTTACTGATGAGTAAACTGAGGCCCAGAGAAGGCAGGTTCACATAG
ELF4 binding site 3

-1120 TCACTGAAGGGAAGAGCTGGAACTTGAACTCTTGTCTGTAGGTCCCAAGTCAGTGTGTGCTGCCTGGCCAACTGGGCAGT

-1040 GCCTGCTCCGTGCTGGGCATGGGCCAGGCCTTGGTCACTGCCCTCAGGGTGGGGACAGACACACTCAGAACGGACTTAGC

-960 ACGTGCCTCCATCAGGGCGTGTCAGGAAGCATGTTGTGGAGAAGGTGGCGTTTACTCTGGACTTTGAAGAACAAGGCGAG
ELF4 binding site 2

-880 GTAGCCAGGGACAGGGGCTGAGCCTTCCTGGCAGGGGTTACAGCCTAGCAAAAGCCCAGAGGGGTAGTTAAAAATAAAG

-801 AGTGCTTCAAACGCTCAATAATGGAACCATTGTGCTGGCCACATCCTCCTGAGAACCTCAGCCGCTGAGCCAGGCCCTTG

-721 AAGTCGGCAGTGGCTTCAGGTGGGAAGCTGCATCTGTGCTTGCAGAACCTTTACTTGTCTAAGTTGCCTCCTCTCTGATAA
ELF4 binding site 1

-640 GAGCATGTAGGGTATGAATGAGAAAGAGATTTCACATGTGCTGAGCGGGCATCACCTCATTTCATCCTTACATGCGTCTTCT

-558 GGGGGCTGTGATATCTTTAGCCCACTCTGCAGGTTGGCATACTGAGGCTTCTTACATTATCTGCAAAGTGGCAGAGTTGAGA

-476 CTTGAACCTGGGTCTGGTCCAAAATCTGTGCTTTTAAGCTCTCTGTCATCCCAGTTCTCGAAAAGCCCAGAATTTTCTGTTG

-394 AACTCCTGGGGATGAGTTTAGTGATGTGCTCGTACATATTGAATAACCAGCTCTCTGGAAGAAAAAAGAGGCTTGATTGGT

-313 AGCGTATTCCGATGTCCAAGGGGTAAATACTCCCACTGTGGCTGACATGGAGCTATCAGTGTGACATTCTTGAATATGGAGT

-231 TGGGAAGAGATGAGCACAGTTGGTTCTTGCAAGTAGGTAAGGGCCAGCTCCAGCGTGTCACTGGGTAAATGTCCCTGCCTC

-150 CCCCCCGAGCCCAGTATGGCCAGTAAAGGCCTTTAGACATGTCTAAGGAATGGAAGAGGGCTGAGAAACCTGCTCCTCCA

-70 GCTGGGGGCCCGCCCTGAGCCCTGGGAAGCTGCGGTTAATCTTTAAGCCAGCCTTGCAAACAAGTGCGGCCATTTCACCA
Transcription starting site (nucleotide +1)

+11 GCCCAGGCTGGCTTCTGCTGTTGACTGGCTGTGGCACCTCAAGCAGCCCCTTTCCCCTCTAGCCTCAGTTTATCACCGCAA

+92 GAGCTACCATTCATCTAGCACAACCTGACCATCCTCACACTGGTCAGTTCCAACCTTCCCAGGAATCTTCTGTGGCCATGTT

+174 CACTCCGGTTTTACAGGTGAGGGGGCT

(c) ELF4 binding sites within the promoter regions of *SRC*. The sequences highlighted in color represent the four binding sites of ELF4 on the SRC promoter, and the arrow represents the transcription initiation site.

Supplementary Data 1.

List of genes differentially expressed in SW480-ELF4 versus SW480-Control cells using RNA-seq.

Genes	logFC	t	P.Value	adj.P.Val	B
MUCL1	-3.31472	-29.1899	3.39E-07	0.004783	6.63854
NOX5	-3.35894	-26.4636	5.77E-07	0.004783	6.35216
PIP5K1C	2.885608	24.33179	9.09E-07	0.004783	6.083033
PHB2	2.18241	23.36406	1.13E-06	0.004783	5.945137
FMNL3	3.270146	21.34424	1.84E-06	0.00535	5.619851
FGFR4	4.474486	21.22749	1.90E-06	0.00535	5.599328
SRC	4.335984	18.88132	3.57E-06	0.006379	5.140284
PDK1	1.768735	18.4262	4.07E-06	0.006379	5.039807
VASP	1.906714	18.34333	4.17E-06	0.006379	5.021068
ILK	1.821931	18.09171	4.49E-06	0.006379	4.963308
CD24	1.923868	17.8395	4.84E-06	0.006379	4.904085
FOXC1	2.792924	17.66935	5.10E-06	0.006379	4.863357
USP9X	1.992378	17.0704	6.13E-06	0.006379	4.714847
HAX1	1.636979	16.9885	6.29E-06	0.006379	4.693896
PIP5K1B	4.137584	16.7266	6.84E-06	0.006379	4.625824
PAK3	3.948549	16.51201	7.33E-06	0.006379	4.568797
ROCK1	2.988215	16.19966	8.12E-06	0.006379	4.483711
CHI3L1	-1.98104	-16.1871	8.15E-06	0.006379	4.480225
SPDL1	1.59486	16.06812	8.48E-06	0.006379	4.447121
CDC42	2.571272	16.027	8.59E-06	0.006379	4.435589
CD63	1.674099	15.90022	8.97E-06	0.006379	4.399753
LOC105373314	-2.1303	-15.6968	9.61E-06	0.006379	4.34134
RAC1	1.547423	15.69124	9.62E-06	0.006379	4.339725
H3C7	-1.78478	-15.5924	9.95E-06	0.006379	4.310923
MSX1	1.168632	15.53051	1.02E-05	0.006379	4.292732
PIP5K1A	2.152869	15.52133	1.02E-05	0.006379	4.290027
ARC	2.414299	15.32344	1.09E-05	0.006379	4.231113
EMP2	1.908222	15.28647	1.11E-05	0.006379	4.219981
RHOA	1.539107	15.27789	1.11E-05	0.006379	4.217393
MARCHF4	2.349416	15.22145	1.13E-05	0.006379	4.200307
NCK1	2.609739	14.88514	1.28E-05	0.006955	4.096538
S1PR4	-1.71192	-14.6023	1.41E-05	0.0071	4.006587
CTSS	1.445002	14.53068	1.45E-05	0.0071	3.983407
WWC1	2.202436	14.35136	1.55E-05	0.0071	3.924651
BIK	-1.55062	-14.3085	1.57E-05	0.0071	3.910447
PTEN	1.539926	14.29679	1.58E-05	0.0071	3.906562
TFR2	1.264132	14.23609	1.62E-05	0.0071	3.886326
RND1	2.591303	14.10913	1.70E-05	0.0071	3.843602
AKT1	1.589262	14.09414	1.71E-05	0.0071	3.838519
ISLR	-1.31279	-14.0388	1.74E-05	0.0071	3.819682

CD8B	-1.33389	-14.002	1.77E-05	0.0071	3.807109
PTK6	2.159468	13.84119	1.88E-05	0.0071	3.75163
VTN	3.148735	13.83015	1.89E-05	0.0071	3.747786
PAK2	1.805264	13.82948	1.89E-05	0.0071	3.747553
LCP1	4.229559	13.82538	1.89E-05	0.0071	3.746124
HOXA5	1.627235	13.6529	2.02E-05	0.007425	3.685494
MUC3A	1.671741	13.46495	2.18E-05	0.007822	3.61821
ABCB1	-2.90531	-13.4026	2.23E-05	0.00785	3.595616
SUSD2	1.695722	13.23231	2.39E-05	0.00823	3.53312
CRK	1.834598	13.13824	2.48E-05	0.008377	3.498129
LGALS9	1.707524	13.00825	2.61E-05	0.008657	3.449221
ADRA2B	1.636504	12.86888	2.77E-05	0.008989	3.396044
EPHA3	3.793415	12.78539	2.86E-05	0.009076	3.363817
FDPS	1.037893	12.70267	2.96E-05	0.009076	3.33161
SDC1	1.586779	12.69998	2.97E-05	0.009076	3.330556
CD47	2.065118	12.66704	3.01E-05	0.009076	3.317651
SLC35D3	-1.80029	-12.586	3.11E-05	0.009162	3.285711
GNAI1	-1.45278	-12.514	3.21E-05	0.009162	3.257093
NOG	1.366994	12.50397	3.22E-05	0.009162	3.253095
DAPK3	1.145329	12.44596	3.30E-05	0.009162	3.229863
CACTIN	2.841983	12.44179	3.31E-05	0.009162	3.228186
GPX5	-4.60333	-12.2753	3.55E-05	0.009679	3.160697
MCAM	1.332637	12.22309	3.63E-05	0.009688	3.139264
USP45	2.48935	12.18793	3.69E-05	0.009688	3.124776
CRKL	2.10335	12.16382	3.73E-05	0.009688	3.114809
MCTP1	-1.90558	-12.1127	3.81E-05	0.009739	3.093596
MMP17	1.675937	12.0648	3.89E-05	0.009739	3.073605
TNNC1	1.227909	12.04827	3.92E-05	0.009739	3.066685
GDA	1.542286	11.90284	4.18E-05	0.010233	3.00526
MIEN1	1.938345	11.78396	4.40E-05	0.010635	2.954336
ITPKC	1.598249	11.62154	4.74E-05	0.01128	2.88369
ARF4	1.276297	11.48652	5.04E-05	0.011649	2.824002
F13A1	2.040248	11.44309	5.14E-05	0.011649	2.804621
MTA1	1.170883	11.43931	5.15E-05	0.011649	2.802927
GMFG	2.279513	11.40183	5.24E-05	0.011649	2.786115
LAMC1	2.022921	11.397	5.25E-05	0.011649	2.783943
NXPH3	-1.28107	-11.3739	5.31E-05	0.011649	2.773539
MMP11	1.944714	11.30362	5.48E-05	0.011881	2.741737
SAA1	2.490759	11.19383	5.77E-05	0.012347	2.691557
FGFR1	1.471536	11.05419	6.16E-05	0.012992	2.626843
CXCR2	2.632991	11.01951	6.27E-05	0.012992	2.610616
GRB2	1.21325	11.00732	6.30E-05	0.012992	2.604895
PEAK1	2.503494	10.97683	6.40E-05	0.013024	2.590558

GPC1	1.117331	10.818	6.90E-05	0.013727	2.515072
ATN1	1.480367	10.79185	6.99E-05	0.01374	2.502514
SNAI2	-1.40433	-10.7243	7.22E-05	0.014036	2.469907
MEGF9	2.302191	10.66831	7.43E-05	0.014262	2.442686
PRSS36	1.288572	10.59247	7.71E-05	0.014638	2.405547
DAAM2	3.642922	10.51247	8.02E-05	0.01506	2.36602
CHRNB4	-1.24497	-10.451	8.27E-05	0.015358	2.335413
BCL2L2-PABPN1	-1.56522	-13.354	9.30E-05	0.0167	2.296074
OTUD6A	-1.41316	-13.3536	9.30E-05	0.0167	2.295964
KANK4	-2.18566	-10.3265	8.80E-05	0.016172	2.272739
TTC30A	-1.12484	-10.2002	9.39E-05	0.0167	2.208237
SCN3B	-1.56273	-10.077	1.00E-04	0.017553	2.144405
RTP3	-1.22461	-10.0627	0.000101	0.017553	2.136962
COL28A1	-1.88129	-10.0386	0.000102	0.017592	2.124357
ITGB1	1.83245	9.916167	0.000109	0.018503	2.059729
MMP9	1.894072	9.884256	0.000111	0.018503	2.042735
TGFB1	1.223059	9.821165	0.000114	0.018763	2.00895
ZAR1	-1.07167	-9.7981	0.000116	0.018771	1.996536
SYPL2	-1.57934	-9.70501	0.000122	0.019076	1.946095
CRYGN	-1.48807	-9.70038	0.000122	0.019076	1.943571
KCNS3	1.415457	9.632252	0.000126	0.019602	1.906282
PTK2	1.721016	9.595414	0.000129	0.019638	1.885994
GGT1	1.297669	9.429779	0.000141	0.021104	1.793673
LRG1	1.420072	9.414374	0.000142	0.021104	1.784994
LOC105378979	3	23.13951	6.62E-05	0.013324	1.766807
KIF2A	2.777738	9.354398	0.000147	0.021623	1.751052
ITGB2	2.657258	9.318897	0.00015	0.021781	1.730846
CCR7	-1.40609	-9.27261	0.000154	0.021781	1.704374
IFITM10	-1.67792	-9.26162	0.000155	0.021781	1.698066
ITGAX	1.346857	9.243628	0.000156	0.021781	1.68772
KRT77	-1.28661	-11.3814	0.000186	0.023305	1.677689
DKK4	-2.51567	-9.20924	0.000159	0.021781	1.667888
FAT1	2.606147	9.204059	0.00016	0.021781	1.664893
MVP	1.247793	9.203989	0.00016	0.021781	1.664852
TGFB2	2.468858	9.191687	0.000161	0.021781	1.657733
LOC107985728	2.168935	18.16023	0.000152	0.021781	1.637788
EPHB2	1.344812	9.132537	0.000167	0.022164	1.623354
RAB3B	1.243169	9.11148	0.000169	0.022254	1.611056
HLA-DRB1	1.697314	9.090637	0.000171	0.022344	1.598852
FBP1	1.428722	9.054238	0.000174	0.022463	1.577466
SLC28A1	-1.84912	-11.0729	0.00021	0.024647	1.56698
PTPRZ1	-3.60828	-9.02687	0.000177	0.022612	1.561323

LY6G6D	-1.10281	-9.01609	0.000178	0.022612	1.554953
GPC4	2.363505	8.990432	0.000181	0.022775	1.539748
CD44	1.805956	8.896542	0.000191	0.023443	1.483712
MMP10	3.366806	8.889127	0.000191	0.023443	1.479259
IFI27	-1.72498	-8.86676	0.000194	0.023577	1.465806
KRT37	-2.15526	-8.83406	0.000198	0.023858	1.446063
KRT6A	-1.56569	-8.82144	0.000199	0.023863	1.438424
AMZ1	-1.5617	-8.71477	0.000212	0.024647	1.37338
NTRK2	-1.22655	-8.70878	0.000213	0.024647	1.369704
PAK4	1.531042	8.707876	0.000213	0.024647	1.369149
SAA2	2.452637	8.698537	0.000214	0.024647	1.363408
MAOB	-1.22989	-8.68162	0.000216	0.024669	1.352995
LOC105372412	1.395424	8.625571	0.000223	0.02484	1.318332
PTCHD3	-1.31468	-10.395	0.000275	0.026926	1.308232
GGT5	1.852645	8.606689	0.000226	0.024856	1.3066
IL20RA	-1.34221	-8.59161	0.000228	0.024856	1.297215
SYNDIG1L	1.542146	8.5635	0.000232	0.024949	1.279664
AK5	1.011442	8.542765	0.000235	0.024949	1.266681
THPO	-3.00106	-8.51644	0.000238	0.025188	1.250149
H3C8	-1.46855	-8.4856	0.000243	0.0255	1.230715
PTCH2	-1.21551	-8.4506	0.000248	0.025885	1.208561
CNGB1	2.806318	8.417397	0.000253	0.026249	1.187463
SYT8	-2.87041	-8.39542	0.000257	0.026279	1.173448
NUDT7	1.06986	8.372567	0.00026	0.026487	1.158834
OR2T8	1.805717	14.57525	0.000321	0.029316	1.148827
ADAM9	2.017617	8.351381	0.000264	0.026507	1.145248
GABRA3	-2.37457	-8.31021	0.00027	0.02687	1.118743
ANKRD30A	-2	-15.4263	0.000265	0.026507	1.113777
CD8B2	-1.05245	-8.2756	0.000276	0.026926	1.096355
NT5E	1.650439	8.274581	0.000276	0.026926	1.095695
VEGFA	1.116555	8.269037	0.000277	0.026926	1.092099
ZACN	-1.74447	-14.1984	0.000351	0.030537	1.083869
SLC26A9	2.008585	8.212615	0.000287	0.027447	1.055363
MMP13	2.903028	8.181163	0.000293	0.027793	1.034771
DOCK11	-1.89518	-8.15827	0.000297	0.027986	1.019731
ZDHHC22	-1.1157	-8.15213	0.000298	0.027986	1.015687
CEND1	-1.84007	-8.12002	0.000304	0.028396	0.994503
TAGLN	-1.14138	-8.05982	0.000316	0.029316	0.954551
ITGB7	3.391247	8.045582	0.000319	0.029316	0.945058
GNAL	-1.11363	-8.02613	0.000323	0.029316	0.932063
ONECUT3	1.518001	8.011528	0.000326	0.029352	0.922281
TMEM132E	-3.27842	-8.00731	0.000326	0.029352	0.919454
NOTCH2	2.074236	7.993197	0.000329	0.029459	0.90998

CPVL	-1.59628	-7.96919	0.000334	0.02971	0.893827
CCL7	-1.84739	-7.95136	0.000338	0.029781	0.881797
SAA2-SAA4	2.566423	9.37238	0.000429	0.033678	0.872949
GALNT12	1.008123	7.931384	0.000343	0.030007	0.868285
POF1B	1.372917	7.888003	0.000352	0.030537	0.838826
COL13A1	1.268128	7.80274	0.000372	0.031616	0.780445
PRSS8	1.227344	7.787214	0.000376	0.031776	0.769745
NCKAP1L	-2.40368	-9.13691	0.000478	0.036383	0.764173
HLA-DPA1	1.521974	7.769581	0.00038	0.031977	0.757567
SLC25A48	-1.73227	-7.75714	0.000383	0.031977	0.748957
CXCL3	-2.10251	-7.68986	0.000401	0.033036	0.70216
PARM1	-2.24996	-7.63809	0.000415	0.033678	0.665874
KLF2	-1.10408	-7.62631	0.000418	0.033678	0.657581
PDE4B	2.116133	7.614762	0.000421	0.033678	0.649442
NTRK3	4.092671	7.614057	0.000421	0.033678	0.648944
SYTL2	-1.67246	-7.59603	0.000426	0.033678	0.636212
ABCC8	-1.52438	-7.57874	0.000431	0.033678	0.623968
SYP	-1.03659	-7.57465	0.000432	0.033678	0.621072
ROCK2	2.304285	7.490137	0.000457	0.035309	0.560809
CD151	1.507756	7.476688	0.000462	0.035468	0.551158
CREB1	1.671807	7.467297	0.000465	0.035531	0.544409
REN	2.808512	8.611891	0.000614	0.042559	0.508871
CPLX3	-1.16181	-7.41596	0.000481	0.036454	0.507368
PLEKHS1	2.782937	8.590695	0.000621	0.04283	0.498175
ANXA1	1.049956	7.375008	0.000494	0.037314	0.477636
MSMO1	1.625468	7.34379	0.000505	0.037947	0.454866
IGF1	2.428242	7.293533	0.000523	0.039102	0.41801
VSTM1	1.890834	7.275449	0.000529	0.03912	0.404689
DBH	-1.52134	-7.2737	0.00053	0.03912	0.403399
SYK	-1.92768	-7.26246	0.000534	0.039252	0.395104
RTN1	-1.87147	-8.37627	0.000691	0.043734	0.38821
CNNM1	-1.21462	-8.364	0.000695	0.043841	0.381819
IL21R	1.819422	7.240644	0.000542	0.039675	0.378959
RFTN1	1.18471	7.184989	0.000564	0.040882	0.33757
GJC2	-1.25359	-7.14483	0.00058	0.041606	0.307513
MUC16	-2.41835	-7.14143	0.000581	0.041606	0.304961
TLL2	-1.25248	-7.1154	0.000592	0.042015	0.285384
KLHL13	-1.57704	-7.09802	0.000599	0.04217	0.272274
ATP8B3	1.508708	7.08524	0.000604	0.04217	0.262617
KLK15	-1.41612	-7.08058	0.000606	0.04217	0.259088
IGF2	-1.43537	-7.0335	0.000627	0.042889	0.223347
TMEM150B	-2.03241	-7.00685	0.000639	0.043182	0.203012
SMAD4	2.388574	6.97327	0.000654	0.043344	0.177285

CYP2E1	1.316509	6.959805	0.00066	0.043344	0.166936
RAF1	1.04109	6.952712	0.000664	0.043344	0.161477
HSPB8	-1.84756	-6.94322	0.000668	0.043344	0.154161
CDK15	1.296303	6.94169	0.000669	0.043344	0.152984
DLGAP3	-1.03861	-6.93899	0.00067	0.043344	0.150904
SMO	-1.0894	-6.937	0.000671	0.043344	0.149365
TEX29	1.919482	6.917622	0.000681	0.043581	0.134395
INSIG1	1.722762	6.907649	0.000686	0.043729	0.126675
SPRY3	-1.17575	-6.89926	0.00069	0.043734	0.120176
HSF5	-1.32193	-10.1963	0.001076	0.055135	0.103405
CHADL	-1.34484	-6.8644	0.000707	0.044277	0.093072
CDH5	3.761801	6.844603	0.000717	0.044552	0.077629
TPGS1	-1.11315	-6.8379	0.000721	0.044552	0.072393
PEBP4	1.456881	10.08434	0.001117	0.056425	0.072047
ERG	-1.73322	-10.0809	0.001118	0.056425	0.071084
IL10RA	-1.26217	-6.83553	0.000722	0.044552	0.070532
ERFL	1.61871	6.797375	0.000742	0.045472	0.040613
CFAP73	-1.5303	-6.79115	0.000746	0.045514	0.035716
NFILZ	1.461994	6.780859	0.000751	0.045691	0.027613
ACOX2	-1.42424	-6.72787	0.000781	0.046882	-0.0143
CPNE9	-1.49664	-6.70145	0.000797	0.046882	-0.0353
PADI3	1.155364	6.698308	0.000798	0.046882	-0.03781
JUN	1.074111	6.678102	0.00081	0.047324	-0.05393
AR	-1.15282	-6.67425	0.000813	0.047324	-0.05701
RBM24	1.066595	6.670818	0.000815	0.047324	-0.05975
FAM189A2	-1.13089	-6.63695	0.000835	0.048036	-0.08691
PGLYRP2	-2.33228	-6.63558	0.000836	0.048036	-0.08801
PRRX1	-3.11726	-6.63224	0.000838	0.048036	-0.09069
LOC105375809	3.490829	6.600523	0.000858	0.048644	-0.11625
LIPG	1.529746	6.594428	0.000862	0.048644	-0.12118
PLD1	1.461858	6.592786	0.000863	0.048644	-0.1225
PITX2	1.180189	6.58045	0.000871	0.048931	-0.13249
PTPN13	-1.2266	-6.53689	0.0009	0.049939	-0.16787
UTS2B	-1.18927	-6.53209	0.000904	0.049939	-0.17178
CYP4F11	1.946701	7.345628	0.001198	0.058358	-0.18913
RBBP8NL	-2.99812	-6.47046	0.000947	0.052069	-0.22223
ISM1	-1.21767	-6.44656	0.000964	0.052069	-0.24192
PHACTR3	1.461036	6.446279	0.000964	0.052069	-0.24215
UNC5A	1.466341	6.445177	0.000965	0.052069	-0.24305
PRR9	-1.64884	-9.01366	0.001626	0.067505	-0.25796
KRT31	-1.51153	-6.42607	0.000979	0.052367	-0.25884
ANO4	-1.37109	-6.42059	0.000983	0.05242	-0.26337
FBLN5	-1.34414	-6.40881	0.000992	0.052603	-0.27313

SMAD2	1.326741	6.397481	0.001001	0.052853	-0.28253
CXorf49B	2.567302	7.169975	0.001325	0.061479	-0.29635
CXorf49	2.567302	7.169975	0.001325	0.061479	-0.29635
H4C8	-1.27674	-6.3524	0.001036	0.054086	-0.32008
FAM184A	-1.05578	-6.34552	0.001041	0.054086	-0.32584
DAPP1	2.640498	6.341046	0.001045	0.054086	-0.32958
FOXD4	-1.93784	-8.77212	0.00178	0.068928	-0.33005
DUSP8	-1.12334	-6.30899	0.001071	0.055027	-0.35647
HDAC9	1.827526	6.279764	0.001096	0.055947	-0.38109
CSF2RB	1.407464	6.265236	0.001108	0.056284	-0.39336
DIRAS3	-2.02346	-6.97087	0.001489	0.064681	-0.42127
HES2	1.0781	6.210042	0.001157	0.057527	-0.44022
TPSG1	-1.22357	-6.20365	0.001163	0.057527	-0.44567
CACNA1E	-2.74294	-6.20213	0.001164	0.057527	-0.44697
ACTA1	-2.03064	-6.92104	0.001533	0.065303	-0.45312
TMEM204	-1.22483	-6.16785	0.001196	0.058358	-0.47629
SLC8A1	2.711903	8.36787	0.002082	0.073491	-0.48671
DAB2	-1.39733	-6.12999	0.001232	0.059497	-0.50883
ABCB4	-1.44275	-6.08505	0.001277	0.061305	-0.54768
CHST8	-1.03131	-6.7631	0.001686	0.068467	-0.55563
C4orf47	-1.11385	-6.06347	0.001299	0.061479	-0.56642
ALPI	-1.62364	-6.05935	0.001303	0.061479	-0.57001
IL1RAPL1	1.033172	6.055884	0.001307	0.061479	-0.57303
MUC13	1.090697	6.05093	0.001312	0.061479	-0.57734
HHEX	1.244102	6.049876	0.001313	0.061479	-0.57826
COL11A2	-1.15304	-6.04846	0.001314	0.061479	-0.5795
TTPA	-1.38766	-5.99859	0.001368	0.062668	-0.62311
ALPK2	2.365837	5.959184	0.001412	0.063902	-0.6578
CD69	-2.14483	-6.60418	0.001859	0.070357	-0.66126
ERVFRD-1	-1.1112	-7.90106	0.002517	0.080576	-0.68041
MSRB3	-1.0487	-5.91847	0.00146	0.064534	-0.69384
ALK	-1.46005	-5.91732	0.001461	0.064534	-0.69486
CXCL2	-1.67854	-5.90893	0.001471	0.064534	-0.70232
PPP1R14D	1.168813	5.90744	0.001473	0.064534	-0.70364
SOX14	-1.34029	-5.87008	0.001518	0.065303	-0.73695
CFAP100	-1.37933	-5.86776	0.001521	0.065303	-0.73902
CHGA	-2.11019	-5.86014	0.001531	0.065303	-0.74583
AKR1C1	-1.24566	-5.86	0.001531	0.065303	-0.74596
H2BC14	2.229698	6.477979	0.002012	0.073061	-0.74697
COL4A4	1.331965	5.840825	0.001555	0.065766	-0.76315
LOC105376747	-2.51329	-12.2944	0.002925	0.085404	-0.7669
GAS2L2	1.292481	6.446332	0.002053	0.073456	-0.76872
RBFOX3	1.433069	6.429507	0.002075	0.073491	-0.78033

GDF5	-2.05664	-6.41676	0.002092	0.073589	-0.78915
H2AC15	-1.99959	-11.9323	0.003144	0.087207	-0.81416
CERKL	-1.86165	-7.52403	0.002957	0.085404	-0.82573
KLK5	-1.86165	-7.52403	0.002957	0.085404	-0.82573
ATP2B4	1.170922	5.752019	0.001674	0.068467	-0.84335
CD68	1.588017	5.747425	0.00168	0.068467	-0.84753
CHRNE	-1.13152	-7.52001	0.002962	0.085404	-0.84956
SYNE1	-1.415	-5.72781	0.001708	0.068467	-0.86539
CHST1	1.019071	5.726248	0.00171	0.068467	-0.86681
AVIL	-1.53184	-5.7218	0.001716	0.068467	-0.87087
LOC112268119	-1.09167	-7.41356	0.003104	0.087105	-0.87388
MLC1	3.385649	5.718474	0.001721	0.068467	-0.87391
NELL2	-1.22592	-5.70791	0.001736	0.06881	-0.88356
CR2	-1.71703	-5.69612	0.001753	0.068928	-0.89435
HOXA3	-1.17868	-5.69165	0.00176	0.068928	-0.89845
IGSF21	-2.14776	-5.68514	0.00177	0.068928	-0.90441
IL2RG	1.106599	5.683948	0.001771	0.068928	-0.90551
UTS2R	-1.05543	-5.68026	0.001777	0.068928	-0.90889
C1QTNF9	-1.53722	-5.66689	0.001797	0.069312	-0.92119
NMU	-1.08092	-5.66474	0.0018	0.069312	-0.92317
ANKRD30B	1.807355	11.07422	0.003765	0.093808	-0.93842
ARX	1.629564	5.624533	0.001862	0.070357	-0.96026
CDH4	-1.82464	-6.17361	0.002447	0.079248	-0.96061
HSD11B1	2.899489	5.612109	0.001882	0.070667	-0.97177
RBM14-RBM4	-1.49112	-5.59809	0.001904	0.071214	-0.98478
CENPVL3	-2.75522	-6.13803	0.002505	0.080576	-0.98624
FNDC5	-1.117	-5.58433	0.001927	0.071733	-0.99757
HLA-DRB3	2.145921	7.132986	0.003522	0.090491	-1.00043
GPRC5D	1.736966	10.64293	0.004143	0.098357	-1.00817
PRPH2	1.736966	10.64293	0.004143	0.098357	-1.00817
LRP2	-2.13732	-5.56322	0.001962	0.072395	-1.01724
NEURL1	-1.48096	-5.55705	0.001972	0.072618	-1.02301
KRT71	-1.53126	-5.54837	0.001987	0.07284	-1.03112
ZP1	-2.49191	-5.53404	0.002011	0.073061	-1.04454
RNF165	-1.3023	-5.48229	0.002102	0.073589	-1.09322
GRIA1	-1.16096	-6.98354	0.003774	0.093808	-1.10623
DPYSL3	-1.53089	-5.45763	0.002148	0.074539	-1.11655
FOXI3	-2.10455	-5.44105	0.002179	0.074933	-1.13228
CCDC3	-1.02656	-5.43469	0.002191	0.074933	-1.13832
TTLL7	-1.29487	-5.43025	0.002199	0.074933	-1.14255
CCN1	1.718808	5.424711	0.00221	0.074933	-1.14782
KCNK4	1.940643	5.891794	0.002955	0.085404	-1.16757
SRSF12	-1.53889	-5.40178	0.002254	0.075598	-1.16968

ABCG5	1.584963	9.711558	0.005163	0.107367	-1.17836
LOC107985876	1.584963	9.711558	0.005163	0.107367	-1.17836
FAM9C	1.584963	9.711558	0.005163	0.107367	-1.17836
NLGN4Y	-1.58496	-9.71156	0.005163	0.107367	-1.17836
RUNDC3B	-1.6133	-5.38709	0.002283	0.075837	-1.18373
MYT1	-2.32576	-5.36074	0.002336	0.077131	-1.209
SPINK9	2.298468	9.511806	0.005427	0.109599	-1.21876
FLG2	-1.43975	-5.81721	0.00311	0.087105	-1.22388
PVALB	-1.23552	-5.33617	0.002387	0.0782	-1.23264
GPR1	-1.50975	-5.32329	0.002414	0.078633	-1.24507
GPR39	2.034487	5.76261	0.00323	0.088203	-1.26553
VSIG2	1.935502	5.750755	0.003257	0.088504	-1.27462
PRR15L	1.064215	6.552633	0.004642	0.104471	-1.28314
TCF20	1.227338	5.276622	0.002516	0.080576	-1.2903
FAM221A	1.071793	5.241862	0.002595	0.081674	-1.32418
SPANXA2	1.890384	8.967976	0.006249	0.116597	-1.33666
CD36	1.452048	5.670393	0.003445	0.089589	-1.33668
DLL1	1.149357	5.228595	0.002626	0.081923	-1.33716
NAV2	-1.13013	-5.22198	0.002641	0.081923	-1.34364
HEPACAM2	-1.53519	-8.91242	0.006342	0.116597	-1.3494
GPR32	1.305512	6.512282	0.004735	0.105097	-1.35145
C19orf84	1.031766	5.21396	0.00266	0.081923	-1.3515
ZNF502	-1.11353	-5.20924	0.002671	0.081948	-1.35614
CCDC116	1.261268	5.205186	0.002681	0.082097	-1.36012
SPATA19	1.531505	8.821873	0.006499	0.117929	-1.37044
TRPM6	-1.28496	-5.18941	0.002719	0.082992	-1.37564
MUC20	-1.32391	-5.18702	0.002725	0.082992	-1.37799
XYLT1	-1.5763	-5.1714	0.002763	0.083263	-1.39339
KRT17	-1.38728	-5.16472	0.00278	0.083616	-1.39999
TMEM233	-1.41504	-8.67037	0.006774	0.119638	-1.40648
CRB2	-1.08441	-5.15232	0.002811	0.084111	-1.41224
CDH11	3.272205	5.138902	0.002845	0.084679	-1.42554
FZD8	1.090656	5.111429	0.002917	0.085404	-1.45284
ASCL2	-1.09595	-5.10021	0.002947	0.085404	-1.46402
FSTL4	-1.35592	-5.0966	0.002957	0.085404	-1.46762
DNAAF1	-1.48755	-5.09416	0.002963	0.085404	-1.47005
VCX3A	-2.14438	-8.3934	0.007319	0.122855	-1.47513
TMEM88	-1.28609	-5.05836	0.003061	0.086964	-1.50587
SLC22A1	-2.71827	-5.04951	0.003086	0.087105	-1.51476
TKTL1	-1.15068	-5.04105	0.00311	0.087105	-1.52326
ZCCHC12	-1.60175	-5.03475	0.003128	0.087105	-1.5296
LYNX1	1.028393	5.01942	0.003172	0.08731	-1.54505
LGR5	-1.32193	-8.09986	0.007967	0.127644	-1.55203

ANKUB1	2.082733	6.146356	0.005706	0.111625	-1.55594
SESN2	-1.06622	-4.98235	0.003282	0.088578	-1.58255
LOC105376525	-1.22403	-4.95969	0.003352	0.088578	-1.60556
SLC38A8	-1.11513	-4.95819	0.003356	0.088578	-1.60709
NEXN	-1.24439	-4.94585	0.003395	0.088962	-1.61966
PCDHGA3	-1.71648	-7.84808	0.008588	0.132685	-1.62161
CCN3	-1.47338	-4.93941	0.003415	0.089086	-1.62623
ZSWIM5	-1.35054	-4.91154	0.003505	0.090491	-1.65472
KIF5C	-1.49428	-4.90866	0.003514	0.090491	-1.65768
TDGF1	-1.46708	-4.90831	0.003515	0.090491	-1.65803
KCNK9	1.309904	5.259027	0.004641	0.104471	-1.66708
KRT38	-1.07264	-4.89799	0.003549	0.09104	-1.66862
PRODH2	1.438599	7.637116	0.009161	0.136315	-1.68264
POMC	-1.28493	-7.63629	0.009164	0.136315	-1.68289
ICAM4	-1.07309	-4.87893	0.003613	0.092395	-1.68821
ADAMTS19	-1.37685	-4.85778	0.003685	0.093271	-1.71001
TNC	-1.06293	-4.85644	0.00369	0.093271	-1.71139
ARHGEF33	-1.37643	-5.20083	0.004848	0.105247	-1.7156
PPP1R16B	-1.37643	-5.20083	0.004848	0.105247	-1.7156
DUOX1	-2.30343	-5.1883	0.004894	0.105247	-1.72611
IGFL1	2.032985	4.838145	0.003754	0.093808	-1.73031
OPRK1	-1.77983	-4.81205	0.003848	0.094641	-1.75737
SP7	-1.51477	-5.69015	0.007299	0.122724	-1.76272
IL6	-1.33904	-7.29255	0.010219	0.143222	-1.78806
LOC101928120	-1.14601	-4.77237	0.003995	0.096804	-1.79873
ANKRD33B	1.354965	4.757054	0.004053	0.097462	-1.81475
PCSK9	1.233992	4.755028	0.004061	0.097511	-1.81687
RAB33A	1.439518	4.747945	0.004089	0.097908	-1.8243
MMD2	-1.16993	-7.16849	0.010642	0.145889	-1.82785
SATL1	-1.16993	-7.16849	0.010642	0.145889	-1.82785
SLC9A4	-1.68954	-4.74158	0.004114	0.098025	-1.83097
LAMP5	-1.76936	-5.03625	0.005496	0.10993	-1.85531
ADH1C	-1.77116	-5.53659	0.00796	0.127644	-1.85651
ANKRD34A	1.039384	4.714131	0.004223	0.09914	-1.85984
C6orf163	-1.12569	-5.01738	0.005576	0.110499	-1.87156
XCR1	-1.27481	-4.68189	0.004356	0.100453	-1.8939
SLC25A52	-1.46446	-6.94739	0.011458	0.150247	-1.90134
LPA	-1.49179	-4.67375	0.00439	0.100878	-1.90252
CXCL17	-1.20402	-4.94577	0.005896	0.113734	-1.9337
PCDHGA7	1.115477	6.834875	0.011907	0.151674	-1.94004
EYA2	-1.66899	-4.63083	0.004576	0.103576	-1.94814
LOC107987243	1.245927	6.785467	0.012112	0.152541	-1.95732
CXCL1	-1.4114	-4.60623	0.004686	0.104916	-1.97442

SLC7A8	-1.42082	-4.60078	0.004711	0.104959	-1.98026
FRMPD3	-2.69227	-4.60067	0.004712	0.104959	-1.98037
FCGR1A	-1.78791	-4.8829	0.006194	0.116201	-1.98885
COLGALT2	-1.21479	-4.58563	0.004781	0.105097	-1.9965
KLK4	-1.03109	-4.5849	0.004785	0.105097	-1.99728
PCDHB15	-1.45345	-6.64544	0.012721	0.155337	-2.00728
MUC5B	1.624823	4.571517	0.004848	0.105247	-2.01166
HLA-DPB1	1.151974	4.568233	0.004863	0.105247	-2.01519
AQP6	-1.1104	-4.56369	0.004885	0.105247	-2.02008
SLC2A12	1.015309	4.535889	0.00502	0.10713	-2.05007
ADGRV1	-1.2478	-4.53218	0.005038	0.10727	-2.05408
LOC101928841	-1.15414	-4.52303	0.005083	0.10727	-2.06398
ZCWPW2	1.389003	4.522446	0.005086	0.10727	-2.06461
PNMA6A	-1.39516	-4.52198	0.005089	0.10727	-2.06512
CNTNAP1	1.078851	4.517836	0.00511	0.107367	-2.06961
LOC107987175	-1.54294	-4.51506	0.005124	0.107367	-2.07262
OXGR1	-1.07023	-4.50863	0.005156	0.107367	-2.07959
LOC101059949	-1.05574	-6.41975	0.013794	0.162087	-2.09096
FGFBP1	2.271082	4.488084	0.005262	0.108349	-2.10191
RSC1A1	-1.20324	-4.48368	0.005285	0.108538	-2.10671
TSPAN10	-1.05056	-4.48073	0.0053	0.108592	-2.10992
ECRG4	-1.09707	-6.36895	0.014053	0.163364	-2.11036
TP53TG3	1.792481	6.360581	0.014096	0.163531	-2.11357
ZC3H11B	-1.39656	-4.46618	0.005377	0.109322	-2.12578
SMAD6	1.038736	4.465666	0.00538	0.109322	-2.12635
TRIM6	1.118981	4.465433	0.005381	0.109322	-2.1266
GSDMA	1.060329	4.460333	0.005408	0.109476	-2.13217
SYNE4	1.178217	4.459183	0.005415	0.109476	-2.13343
PRRT4	-1.03579	-4.45091	0.005459	0.10992	-2.14246
TBX1	1.100757	4.44787	0.005476	0.10992	-2.1458
C3orf80	-1.9945	-4.44552	0.005489	0.10992	-2.14837
ARMCX2	-1.6667	-4.44305	0.005502	0.10993	-2.15108
KIF5A	-1.07415	-4.43238	0.005561	0.110499	-2.16277
KRT72	-1.89461	-5.05816	0.01057	0.145486	-2.16702
LOC112267971	-1.16297	-6.17385	0.015112	0.16817	-2.18682
FAM3B	1.529148	4.382181	0.005847	0.113334	-2.21801
USP17L11	3.459432	14.11766	0.015888	0.170084	-2.2181
RIMS4	-1.26482	-4.37056	0.005916	0.113854	-2.23086
GOLGA6D	-3.37851	-13.7874	0.016439	0.17321	-2.2312
SPATA48	-1.05774	-6.03472	0.015936	0.170488	-2.24333
KCNB1	-1.18144	-4.35293	0.006022	0.114379	-2.25038
SPON1	1.186596	4.328743	0.00617	0.116095	-2.27725
TEKT3	-1.44519	-4.32698	0.006181	0.116095	-2.27922

GAP43	-3.05682	-4.30759	0.006304	0.116597	-2.30083
ADCY4	1.244901	4.303243	0.006332	0.116597	-2.30568
CALCB	-1.55198	-4.5317	0.008234	0.130174	-2.3075
ESRRB	-1.82098	-4.29991	0.006353	0.116597	-2.3094
CD300C	1.374617	4.288092	0.00643	0.117375	-2.32262
FAM71D	1.661841	4.286363	0.006441	0.117455	-2.32455
PPY	-1.42251	-4.2835	0.00646	0.117633	-2.32776
SLC35F3	-1.92466	-4.28275	0.006465	0.117633	-2.32859
DNAJB13	1.157965	4.824069	0.01224	0.152839	-2.32972
NPR3	-2.80735	-11.4566	0.021457	0.193723	-2.35093
ADAM23	-2.92438	-4.25541	0.006648	0.119226	-2.35927
TNNI2	-2.19182	-4.78097	0.012583	0.154907	-2.36049
KRT83	1.453945	4.254163	0.006657	0.119226	-2.36068
PSTPIP1	-1.29288	-4.25294	0.006665	0.119226	-2.36205
SNAP91	-1.34325	-4.24913	0.006691	0.119299	-2.36634
HSPA1B	1.539813	4.241839	0.006741	0.119562	-2.37454
KISS1	2.509289	4.230921	0.006817	0.119951	-2.38685
GRK1	-1.5128	-4.44638	0.008845	0.134113	-2.3877
KCNA6	-2.07014	-4.23015	0.006822	0.119951	-2.38772
SLFN12L	-1.15445	-4.21114	0.006957	0.120589	-2.40919
PLA2G5	2.486524	4.209643	0.006967	0.120589	-2.41089
TBX18	1.134979	4.207936	0.00698	0.120599	-2.41282
MAP3K15	-1.07202	-4.20621	0.006992	0.120599	-2.41478
DMD	2.584963	10.54902	0.024155	0.203734	-2.41501
GALR2	-2.04042	-4.18162	0.007172	0.122488	-2.44265
CTAGE6	2.459432	10.03674	0.025942	0.210011	-2.45708
PDZK1IP1	1.798556	4.163916	0.007304	0.122724	-2.46279
MAGEC1	-1.18527	-4.15821	0.007347	0.123084	-2.46928
PON1	-1.83062	-4.62245	0.013953	0.162991	-2.47593
SLC13A4	1.788291	4.348751	0.00961	0.139555	-2.48082
C20orf202	2.369234	9.668653	0.027369	0.214272	-2.49041
GRM6	-1.18281	-4.13407	0.007534	0.124318	-2.49683
LOC107986453	-1.47899	-4.13345	0.007539	0.124318	-2.49755
C10orf67	-1.16692	-4.13198	0.00755	0.124318	-2.49923
CACNG6	1.015629	4.130846	0.007559	0.124318	-2.50052
HLA-DRA	1.023258	4.12355	0.007617	0.124996	-2.50887
UGT2B15	2.321928	9.475603	0.028171	0.217428	-2.50903
LITAFD	2.321928	9.475603	0.028171	0.217428	-2.50903
BCL2L14	1.689224	4.120722	0.007639	0.125121	-2.51211
APCDD1	-1.01967	-4.11839	0.007658	0.125188	-2.51478
DEFB131B	-2.17102	-5.40543	0.020562	0.190894	-2.5215
HTRA1	-1.46908	-4.10876	0.007735	0.125678	-2.52582
BHMG1	-1.65541	-4.07961	0.007974	0.127644	-2.55932

LOC105372798	-1.17668	-4.07831	0.007985	0.127698	-2.56082
TNFAIP8L2	1.0025	5.32199	0.021311	0.193723	-2.56137
USP17L22	2.169925	8.85529	0.031035	0.227189	-2.5748
SLC6A17	-2.16993	-8.85529	0.031035	0.227189	-2.5748
NEUROG2	-2.16993	-8.85529	0.031035	0.227189	-2.5748
FHL1	1.139054	4.048386	0.00824	0.130174	-2.59536
WNT16	-1.52805	-4.04629	0.008258	0.130217	-2.59778
ALPP	-1.02171	-4.04363	0.008282	0.130218	-2.60086
MFAP4	-1.13152	-5.17352	0.022739	0.197954	-2.6342
GIP	-1.69816	-4.01464	0.008539	0.132661	-2.63449
EOMES	-1.29624	-4.01117	0.00857	0.132685	-2.63851
SLC46A2	-1.34619	-4.18606	0.011068	0.147424	-2.63932
TMEM140	-2.03847	-8.31885	0.033931	0.236628	-2.64004
UNC13C	-1.05664	-4.00946	0.008586	0.132685	-2.64051
HAS2	-1.05577	-4.00179	0.008656	0.133007	-2.64942
MIXL1	-1.04916	-4.00054	0.008667	0.133007	-2.65088
COL25A1	-2	-8.16184	0.034866	0.23947	-2.66079
IGLL5	2	8.16184	0.034866	0.23947	-2.66079
ZMAT4	-2	-8.16184	0.034866	0.23947	-2.66079
SEZ6	-2	-8.16184	0.034866	0.23947	-2.66079
MYO3A	-2	-8.16184	0.034866	0.23947	-2.66079
WDR72	-1.70007	-3.98591	0.008803	0.134052	-2.66793
DSCAML1	1.251629	3.984822	0.008813	0.134086	-2.6692
SIRPG	-1.51324	-5.10153	0.023479	0.201247	-2.6704
FOXP1	1.114952	3.981246	0.008847	0.134113	-2.67337
H2AC7	1.96801	8.031291	0.035676	0.24199	-2.67868
MYL4	-2.874	-4.35318	0.016738	0.174728	-2.68045
PGLYRP1	-1.50706	-4.13872	0.01154	0.15071	-2.68622
HLA-DRB5	2.427318	3.969286	0.00896	0.134982	-2.68734
SLC26A8	-1.20244	-3.96712	0.008981	0.134982	-2.68987
CSAG2	1.943416	7.930927	0.036321	0.244394	-2.69283
CD300LF	-1.9386	-7.91127	0.03645	0.244394	-2.69564
CCDC200	1.159513	3.956537	0.009083	0.13598	-2.70226
JPH4	-1.77275	-3.9499	0.009147	0.136315	-2.71003
POU4F3	-1.02191	-4.10907	0.011848	0.151609	-2.71578
TAMALIN	-2.39409	-3.93219	0.009322	0.137734	-2.73082
CDH2	-1.56464	-4.97681	0.024842	0.206036	-2.73453
MTRNR2L7	-1.00428	-4.91267	0.025585	0.207916	-2.76822
EML1	-1.30595	-3.89937	0.009656	0.139977	-2.76946
ASB15	-1.80735	-7.37567	0.040273	0.254383	-2.77793
CD177	1.215661	4.038023	0.012626	0.154907	-2.78719
LOC645202	-1.7885	-7.29871	0.040877	0.256563	-2.79071
ZBED3	-1.06004	-3.87848	0.009876	0.141824	-2.79415

TSPAN12	-1.16013	-3.87671	0.009894	0.141894	-2.79625
HSPA8	1.203956	3.874875	0.009914	0.141894	-2.79841
SPATA31A3	1.160964	4.318698	0.017144	0.176898	-2.80395
CD101	-1.36562	-3.86927	0.009974	0.142394	-2.80505
HTR3E	-1.76553	-7.20501	0.041634	0.258276	-2.80661
RASGEF1C	-1.01174	-3.86736	0.009995	0.142525	-2.80731
HLF	-1.58496	-3.86131	0.01006	0.14256	-2.81449
GZMM	-1.30137	-4.30518	0.017306	0.177392	-2.81484
CLEC2B	-1.16181	-4.17167	0.019015	0.184829	-2.82456
PDE2A	1.120422	3.852129	0.010161	0.143222	-2.82538
THEMIS	1.160964	4.804447	0.026911	0.212659	-2.82621
SLC47A2	1.160964	4.804447	0.026911	0.212659	-2.82621
MUC4	-1.82602	-3.84772	0.010209	0.143222	-2.83062
KRT81	1.70881	3.843817	0.010253	0.143222	-2.83525
TNS1	-1.41326	-3.84003	0.010295	0.143456	-2.83976
PCDHGA12	1.716207	7.003703	0.043342	0.263331	-2.84213
KRT7	1.789021	3.836851	0.01033	0.143833	-2.84354
GOLGA8G	-1.70044	-6.93936	0.043913	0.264024	-2.85389
SPATA18	-1.07991	-3.81826	0.010541	0.14533	-2.86568
GAL3ST2	-1.13242	-3.81325	0.010599	0.145769	-2.87167
OR2B6	-1.58684	-3.8059	0.010684	0.145889	-2.88044
BPI	1.468458	3.80537	0.01069	0.145889	-2.88107
ZBTB7C	-1.44593	-3.80293	0.010719	0.145889	-2.88399
AQP5	-1.27189	-3.80135	0.010737	0.145889	-2.88587
CCL20	-1.92307	-3.79671	0.010791	0.145955	-2.89142
MEX3B	1.056073	3.793648	0.010828	0.146298	-2.89509
SFTA2	1.646363	6.718676	0.045971	0.270117	-2.89578
TRIML1	-1.22972	-4.19779	0.018665	0.183581	-2.90235
TAL1	-2.10544	-3.92026	0.014052	0.163364	-2.90734
OR10AD1	-1.62803	-6.64387	0.046705	0.272131	-2.91055
GALNT5	2.156987	3.777438	0.011021	0.147054	-2.9145
LRRTM2	-1.07627	-3.90446	0.014257	0.164613	-2.92363
MPL	-1.09734	-3.76417	0.011182	0.148363	-2.93043
CLIP4	1.423642	3.761937	0.01121	0.14861	-2.93311
CD22	1.005165	3.760358	0.011229	0.148751	-2.93501
LOC100505502	1.730882	4.025697	0.021132	0.193396	-2.94426
CT47A2	1.584963	6.468105	0.048509	0.275523	-2.94644
GIPC3	-1.58496	-6.46811	0.048509	0.275523	-2.94644
FBN1	1.584963	6.468105	0.048509	0.275523	-2.94644
C3orf56	1.584963	6.468105	0.048509	0.275523	-2.94644
POTED	-1.58496	-6.46811	0.048509	0.275523	-2.94644
CD300LB	-1.58496	-6.46811	0.048509	0.275523	-2.94644
CYP24A1	1.302662	3.747713	0.011386	0.150241	-2.95021

SPTLC3	-2.74345	-3.74663	0.0114	0.150247	-2.95152
ASCL5	-1.16079	-3.74441	0.011428	0.150247	-2.9542
NPNT	-1.35222	-3.74274	0.011448	0.150247	-2.9562
LILRB5	-1.09416	-3.74237	0.011453	0.150247	-2.95664
SKOR1	-1.73283	-3.86692	0.01476	0.166768	-2.9625
RTL5	-1.05363	-3.73641	0.011528	0.150707	-2.96382
C8orf34	-1.23211	-3.86068	0.014845	0.167064	-2.96899
NTM	-1.15299	-3.73067	0.011601	0.151012	-2.97075
GPX2	-1.35395	-3.72875	0.011626	0.151012	-2.97307
TSPAN7	-1.4183	-3.84932	0.015002	0.16815	-2.98081
CACNA1D	-1.41235	-3.71831	0.011761	0.151172	-2.98568
PPP1R2B	-1.20276	-3.84275	0.015094	0.16817	-2.98765
FER1L5	-1.57142	-3.71289	0.011831	0.151609	-2.99222
H3C15	-1.06023	-3.83257	0.015238	0.168674	-2.99828
PTPRN	-1.57142	-3.70773	0.011899	0.151674	-2.99846
GCNT2	-1.33817	-3.69551	0.012061	0.152452	-3.01326
CXCL8	-2.13036	-3.6949	0.012069	0.152452	-3.014
CYP4X1	-2.29787	-3.6949	0.012069	0.152452	-3.014
PRB3	-1.00452	-3.6946	0.012073	0.152452	-3.01436
NFKBIZ	-1.3104	-3.68789	0.012163	0.152745	-3.0225
IL7R	2.087492	3.68778	0.012164	0.152745	-3.02263
LAMC2	1.193403	3.686031	0.012188	0.152745	-3.02475
CSDC2	-1.57507	-3.80572	0.015624	0.169202	-3.02637
TEX13C	2.056642	3.682866	0.012231	0.152839	-3.02859
LOC112267947	-1.4833	-3.79396	0.015797	0.169716	-3.03871
LOC107986244	-1.84432	-3.67278	0.012368	0.153515	-3.04084
PIWIL2	-1.58758	-3.6595	0.012552	0.154907	-3.057
PDE3B	-1.06833	-3.65514	0.012613	0.154907	-3.0623
OTOG	-1.91232	-3.65302	0.012643	0.154907	-3.06489
KRTAP5-6	-1.61598	-3.76853	0.016178	0.171632	-3.06548
KCNS1	1.103007	3.628211	0.012998	0.157194	-3.09516
GGTLC2	-1.06006	-3.74035	0.016613	0.174172	-3.09526
APCDD1L	-2.22591	-3.61892	0.013133	0.158116	-3.10652
RASL10A	1.246309	3.615811	0.013179	0.158411	-3.11032
HMGCS1	1.347161	3.605018	0.013339	0.159414	-3.12354
NUTM2E	-1.28206	-3.71254	0.017056	0.176226	-3.12479
CYP4A22	-1.20752	-4.28485	0.034792	0.239451	-3.12576
CLEC1A	1.335	3.593319	0.013516	0.160557	-3.13789
CCL27	-1.10455	-3.58855	0.013588	0.160729	-3.14374
RETREG1	-1.53808	-3.56984	0.013877	0.162667	-3.16674
MROH2A	1.362825	3.672777	0.017714	0.179187	-3.16722
CACNA1G	1.100361	3.553979	0.014128	0.163674	-3.18629
EDN3	-1.97543	-3.65371	0.01804	0.180334	-3.18765

KRT86	1.315089	3.550284	0.014187	0.16418	-3.19085
PAX3	1.008961	4.175408	0.036846	0.245023	-3.19363
TSPAN19	-1.00896	-4.17541	0.036846	0.245023	-3.19363
KRT2	1.03787	3.531975	0.014483	0.165639	-3.21347
PRR22	-1.12211	-3.52564	0.014588	0.166405	-3.22131
LOC728392	3.027678	3.523183	0.014628	0.166424	-3.22435
PRKAR2B	-1.03991	-3.52125	0.01466	0.166424	-3.22673
CNTNAP2	-1.66667	-3.51895	0.014699	0.166631	-3.22958
CTAGE1	-1.36848	-3.81147	0.024799	0.206036	-3.23222
CDK14	-1.19499	-3.68726	0.027288	0.214023	-3.23534
EN1	-1.56848	-3.50748	0.014891	0.16736	-3.2438
MAT1A	-1.06858	-3.49631	0.015082	0.16817	-3.25767
CA2	-1.51477	-3.65787	0.027922	0.216795	-3.26154
AKR1C2	1.096705	3.492685	0.015144	0.168303	-3.26217
BCL2L10	-1.03372	-3.48781	0.015228	0.168674	-3.26823
UGT1A4	-1.42366	-3.46235	0.015677	0.169285	-3.29993
TCF24	1.026712	3.456533	0.015782	0.169696	-3.30719
MT3	-1.11752	-3.44663	0.015962	0.170532	-3.31955
OR2A4	1.396124	3.709993	0.02681	0.212659	-3.32292
MEGF10	-1.63563	-3.43364	0.016201	0.171695	-3.3358
ANKRD22	1.481595	3.565112	0.030044	0.224906	-3.34521
LOC107986762	-1.09181	-3.41998	0.016458	0.17321	-3.35291
PITX3	-1.77888	-3.49179	0.021108	0.19336	-3.36364
CUX2	-1.18878	-3.41141	0.016621	0.174172	-3.36364
B3GAT2	-1.37917	-3.41059	0.016636	0.174229	-3.36468
IDI1	1.068843	3.407948	0.016687	0.174543	-3.36799
CYP7B1	2.163951	3.487513	0.021197	0.193465	-3.36835
RNF182	-1.07873	-3.40322	0.016778	0.174847	-3.37392
G6PC	-1.22891	-3.4004	0.016833	0.175093	-3.37747
PSORS1C1	-1.1445	-3.39768	0.016886	0.175108	-3.38089
TEDDM1	-1.03519	-3.62464	0.02866	0.219662	-3.40055
CXCR1	-1.57899	-3.37827	0.017268	0.177392	-3.4053
TENM2	-1.33459	-3.37818	0.01727	0.177392	-3.40542
CDKL2	-1.84362	-3.37732	0.017287	0.177392	-3.4065
DSEL	-1.24222	-3.44797	0.022041	0.194827	-3.41202
RNF180	-1.8562	-3.44733	0.022054	0.194827	-3.41272
CLUL1	-1.45641	-3.3704	0.017427	0.17809	-3.41523
GABRA2	-1.25163	-3.43133	0.022407	0.19675	-3.43046
NUTM2B	-1.11379	-3.35753	0.017688	0.179187	-3.43145
LRRC69	-1.02594	-3.35358	0.01777	0.17964	-3.43645
EVX1	-2.68068	-3.35122	0.017818	0.179809	-3.43942
DNAJC12	-1.68934	-3.35039	0.017835	0.179876	-3.44047
LOC112268350	-1.3101	-3.34408	0.017966	0.180334	-3.44844

NRG4	-1.25135	-3.33454	0.018167	0.180814	-3.46052
LCE1B	1.265257	3.766	0.046186	0.270696	-3.46372
H2AC17	-1.51914	-3.33163	0.018228	0.181043	-3.4642
ABCA6	-1.06547	-3.3199	0.018479	0.182555	-3.47905
SLFN14	-1.2678	-3.31816	0.018517	0.18282	-3.48126
CLDN2	1.380411	3.384461	0.023478	0.201247	-3.48267
CEMIP	-1.50163	-3.31333	0.018621	0.183581	-3.48739
STC1	-1.33644	-3.31076	0.018677	0.183581	-3.49065
NLRP1	1.495807	3.310679	0.018679	0.183581	-3.49075
C2orf88	-1.16395	-3.30525	0.018798	0.183956	-3.49765
TBX19	1.124627	3.301137	0.018888	0.184347	-3.50287
C10orf90	1.81816	3.300583	0.0189	0.184347	-3.50357
SDR42E2	-1.13152	-3.50417	0.031547	0.22896	-3.51224
ADGRG2	-1.33442	-3.29374	0.019052	0.185082	-3.51228
IL36G	-2.63922	-3.2884	0.019172	0.185297	-3.51906
KRT84	-1.28473	-3.28833	0.019173	0.185297	-3.51915
CXCL6	-1.89895	-3.28169	0.019323	0.185686	-3.5276
EPN3	-1.04052	-3.27694	0.019431	0.186204	-3.53365
KRT32	-1.41885	-3.27426	0.019492	0.186351	-3.53706
BIRC3	1.077543	3.273424	0.019511	0.18643	-3.53813
KIF19	1.45899	3.266464	0.019671	0.186822	-3.547
TBC1D10C	-1.05023	-3.259	0.019844	0.187336	-3.55653
PSG5	1.559619	3.25738	0.019882	0.187336	-3.55859
ABCA13	-1.7664	-3.25563	0.019923	0.187336	-3.56083
GRHL3	1.320513	3.255326	0.01993	0.187336	-3.56121
CTSE	1.105407	3.254671	0.019945	0.187336	-3.56205
AVPR1B	1.584963	3.44959	0.032973	0.233588	-3.56366
KIAA1549L	1.242628	3.250826	0.020036	0.187418	-3.56696
PREX2	-1.33333	-3.24829	0.020096	0.187703	-3.5702
ZNF415	-1.93977	-3.29054	0.025807	0.209386	-3.58836
MMP7	1.984358	3.270149	0.026348	0.211835	-3.61149
SPDYE18	-2.59347	-3.21532	0.020892	0.192243	-3.61241
SLC16A14	-1.91746	-3.21102	0.020999	0.193007	-3.61791
URAD	-1.84674	-3.20337	0.02119	0.193465	-3.62773
H3-5	1.822893	3.263333	0.038472	0.249192	-3.62793
HSPA1A	1.412249	3.19786	0.021328	0.193723	-3.63481
SERPINB2	1.880455	3.197109	0.021347	0.193723	-3.63578
NUDT11	-1.39666	-3.19507	0.021399	0.193723	-3.63839
TP53AIP1	-1.35188	-3.19074	0.021509	0.193723	-3.64396
SERPINE1	1.53839	3.189955	0.021529	0.193723	-3.64497
PPP1R32	1.107184	3.188793	0.021559	0.193723	-3.64646
ANKRD53	-1.72729	-3.18783	0.021583	0.193723	-3.6477
MRGPRE	-1.13752	-3.18649	0.021618	0.193723	-3.64942

CAPN12	1.025251	3.185456	0.021644	0.193723	-3.65076
SAXO1	-1.37145	-3.1829	0.02171	0.193723	-3.65404
CPED1	-1.46054	-3.1719	0.021995	0.194827	-3.6682
GIMAP6	1.539326	3.169594	0.022056	0.194827	-3.67118
LOC102724265	-1.00597	-3.16944	0.02206	0.194827	-3.67137
LOC105370733	-1.68085	-3.16852	0.022084	0.194827	-3.67257
LOC107984832	-1.05067	-3.20891	0.040286	0.254383	-3.68065
PPIAL4G	1.39068	3.321589	0.036639	0.245023	-3.68629
CTLA4	-1.33089	-3.31704	0.036778	0.245023	-3.69069
ZNF90	-1.23808	-3.19561	0.028438	0.218295	-3.69662
GDF15	-1.3779	-3.12599	0.023233	0.20046	-3.72747
HMGCS2	-1.14654	-3.12466	0.02327	0.200581	-3.7292
ALPK1	1.00636	3.123359	0.023306	0.200581	-3.73089
AMN	1.136897	3.121075	0.02337	0.200824	-3.73384
SLC6A2	-1.36848	-3.16249	0.029428	0.22206	-3.73473
MPPED2	-1.36776	-3.11769	0.023465	0.201247	-3.73823
LIPN	1.376428	3.112385	0.023614	0.202019	-3.7451
ZNF300	-1.61748	-3.13795	0.042812	0.261812	-3.75019
CYP39A1	-1.13384	-3.10758	0.023751	0.202404	-3.75133
CPO	-1.19499	-3.0991	0.023993	0.203285	-3.76232
LOC107985021	-1.52832	-3.09318	0.024164	0.203734	-3.77001
ALOX15	-2.003	-3.08645	0.02436	0.204856	-3.77874
ITIH3	-1.32241	-3.10036	0.044228	0.264875	-3.78739
MKX	-1.74986	-3.07807	0.024607	0.205644	-3.78964
COL4A3	2.129195	3.075786	0.024675	0.205999	-3.79261
SLC44A5	-1.0096	-3.0739	0.024731	0.206033	-3.79507
TIGD2	-1.13721	-3.06815	0.024903	0.206038	-3.80255
EGR2	-1.57941	-3.06665	0.024948	0.206038	-3.80451
SAA4	1.305115	3.064659	0.025008	0.206297	-3.80709
ALOX5AP	1.306818	3.063829	0.025033	0.206403	-3.80817
DUX4	-1.18342	-3.05618	0.025265	0.206902	-3.81814
HMCN2	1.359334	3.051779	0.025399	0.207001	-3.82387
HFM1	-1.36425	-3.05593	0.045976	0.270117	-3.83169
ZIK1	-2.03814	-3.07418	0.032266	0.231337	-3.83718
PPM1E	-1.41086	-3.0314	0.026032	0.210463	-3.85045
TNFRSF11B	-1.02389	-3.01952	0.02641	0.211835	-3.86599
ABCG2	-1.05664	-3.01822	0.026451	0.211835	-3.86768
RUBCNL	1.015168	3.017319	0.02648	0.211835	-3.86886
TNFRSF8	-1.38006	-3.00694	0.026816	0.212659	-3.88244
TOMM20L	-1.50911	-3.00427	0.026903	0.212659	-3.88594
SCRT2	-1.23697	-3.11555	0.043649	0.264024	-3.88971
CAPN3	-1.42678	-2.98786	0.027445	0.214767	-3.90745
ART4	-1.20853	-2.98106	0.027673	0.215735	-3.91636

PDE1B	1.020269	2.971209	0.028007	0.216948	-3.92929
LOC107985555	-1.87434	-2.97075	0.028023	0.216948	-3.9299
TLR9	1.562211	2.96934	0.028071	0.217053	-3.93174
GPA33	-2.30818	-2.96233	0.028312	0.21814	-3.94095
PTGER2	-1.05286	-2.94498	0.028918	0.220778	-3.96377
IGFBPL1	-1.11161	-2.93848	0.029149	0.221175	-3.97233
NFIB	-1.14245	-2.93459	0.029288	0.221403	-3.97745
UNC45B	-1.37744	-3.02565	0.047215	0.273296	-3.9808
PCDHGA5	-1.10858	-2.94981	0.036813	0.245023	-3.98349
REG3A	-1.56651	-2.94265	0.037096	0.245609	-3.99197
CNIH2	-1.10289	-2.92263	0.029721	0.223374	-3.99322
CYP3A4	1.114903	2.932824	0.037489	0.24628	-4.00364
CNIH3	-1.34339	-2.91245	0.030094	0.225182	-4.00666
MYH7B	-1.09032	-2.91011	0.030181	0.225318	-4.00973
TRAPPC3L	-1.17781	-2.91	0.030185	0.225318	-4.00988
TPSAB1	-2.20723	-2.90594	0.030336	0.225318	-4.01525
RTP4	1.03112	2.891043	0.030897	0.226966	-4.03493
HIGD1C	-1.30485	-2.88352	0.031184	0.227531	-4.04488
GP1BB	1.434064	2.876519	0.031455	0.228482	-4.05415
FLRT3	-1.869	-2.87085	0.031675	0.229204	-4.06165
LOC112267876	-1.80842	-2.86382	0.031952	0.230068	-4.07097
LZTS1	-1.04072	-2.86317	0.031977	0.230102	-4.07183
COL8A1	-1.81981	-2.85515	0.032296	0.231337	-4.08245
GHR	-1.06594	-2.84839	0.032567	0.232404	-4.09142
OSCAR	1.01963	2.843437	0.032767	0.233205	-4.098
FOXC2	-1.19232	-2.81137	0.034096	0.237271	-4.14061
PABPC3	1.067613	2.80679	0.034291	0.237603	-4.1467
DSC3	-1.10731	-2.80511	0.034363	0.237853	-4.14894
MUC22	-1.63291	-2.81057	0.042796	0.261807	-4.14994
KRT85	3.334535	2.80156	0.034515	0.238319	-4.15366
NBPF7	-1.09711	-2.7998	0.03459	0.238579	-4.15601
FOSB	2.288576	2.797219	0.034702	0.239024	-4.15945
ALLC	-2.06471	-2.78332	0.035308	0.241178	-4.17796
EXOC3L2	-1.77513	-2.77897	0.0355	0.2416	-4.18377
STMND1	-1.42286	-2.77186	0.035816	0.242565	-4.19324
LGALS4	-1.25482	-2.77164	0.035826	0.242565	-4.19355
LOC105377310	-1.50553	-2.75949	0.036374	0.244394	-4.20976
KRT36	-1.22408	-2.75493	0.036582	0.244985	-4.21585
SERPINA2	1.038049	2.753439	0.03665	0.245023	-4.21784
C11orf91	-1.45805	-2.75303	0.036669	0.245023	-4.2184
DEFB134	-1.35233	-2.74614	0.045932	0.270117	-4.22786
SPSB4	-1.29632	-2.72429	0.047054	0.27306	-4.25441
LOC107985433	-1.06636	-2.72571	0.037946	0.2475	-4.25493

KLHL14	-1.25902	-2.72524	0.037968	0.2475	-4.25555
GNGT2	1.242732	2.714644	0.038476	0.249192	-4.26974
NACA2	-1.51212	-2.70888	0.038756	0.249941	-4.27746
BHLHE23	-1.14111	-2.7074	0.038828	0.250117	-4.27944
JSRP1	1.136526	2.698563	0.039262	0.251064	-4.29128
PATE4	-1.35555	-2.69768	0.039306	0.251064	-4.29247
GPR150	1.351939	2.688655	0.048952	0.276443	-4.29784
LOC105377650	-1.51632	-2.69207	0.039584	0.251759	-4.29999
SPATA32	1.473256	2.677964	0.040294	0.254383	-4.31892
CHGB	-1.7514	-2.66631	0.040891	0.256563	-4.33456
TSNAXIP1	-1.3388	-2.65374	0.041546	0.258209	-4.35146
DSG1	-1.0755	-2.64874	0.041809	0.258866	-4.35818
IGFL2	-1.02446	-2.63389	0.042603	0.261099	-4.37815
SHC4	-1.0106	-2.61444	0.043667	0.264024	-4.40434
FILIP1L	-1.19875	-2.61333	0.043728	0.264024	-4.40583
GPER1	-1.62392	-2.60573	0.044153	0.264562	-4.41608
EXOC3L1	-1.81749	-2.59485	0.044768	0.266821	-4.43074
ABCG8	-1.09825	-2.59139	0.044965	0.267716	-4.43541
PLA2G2D	-1.11368	-2.58461	0.045355	0.268714	-4.44455
ECSCR	1.374629	2.576133	0.045847	0.270114	-4.45599
LOC107984590	-2.53814	-2.57513	0.045906	0.270117	-4.45735
SERTAD4	-1.42537	-2.55069	0.04736	0.273405	-4.49035
PBX1	-1.1011	-2.54908	0.047458	0.273775	-4.49252
SCGB3A1	-1.53652	-2.53883	0.048084	0.275231	-4.50637
NLGN1	-1.3023	-2.53707	0.048192	0.275387	-4.50875
ANO2	1.262735	2.527725	0.048772	0.276167	-4.52139
DRD4	1.110339	2.525033	0.048941	0.276443	-4.52503
C7orf61	1.010439	2.520467	0.049228	0.276904	-4.53121
KRT35	-1.94308	-2.51444	0.04961	0.277943	-4.53936

Supplementary Data 2.

Top 10 GO terms and KEGG pathways of DEGs between SW480-ELF4 group and SW480-control group.

GO:BP

Description	GeneRatio	p.adjust
Locomotion	154/703	3.55E-14
Biological adhesion	128/703	2.36E-14
Cell migration	128/703	4.01E-12
Cell population proliferation	125/703	1.36E-06
Cell-cell signaling	124/703	8.48E-10
Regulation of cell differentiation	122/703	4.84E-10
Regulation of transport	122/703	1.99E-08
Positive regulation of signaling	119/703	9.58E-08
Homeostatic process	115/703	7.56E-05
Response to endogenous stimulus	114/703	8.95E-08

GO:CC

Description	GeneRatio	p.adjust
Intrinsic component of plasma membrane	137/594	2.24E-11
Plasma membrane region	104/594	1.73E-10
Synapse	92/594	1.80E-05
Neuron projection	90/594	1.02E-04
Golgi apparatus	89/594	2.83E-02
Supramolecular complex	78/594	9.07E-03
Secretory vesicle	76/594	2.32E-05
Cell surface	70/594	1.26E-05
Supramolecular polymer	67/594	1.12E-03
Secretory granule	60/594	1.10E-03

GO:MF

Description	GeneRatio	p.adjust
Signaling receptor binding	119/656	5.08E-07
Molecular transducer activity	93/656	4.68E-03
Protein containing complex binding	74/656	3.85E-02
Calcium ion binding	49/656	1.70E-02
Receptor regulator activity	45/656	1.17E-03
Cell adhesion molecule binding	44/656	2.13E-03
G protein coupled receptor binding	26/656	1.20E-02
Glycosaminoglycan binding	25/656	1.47E-03
Peptide binding	25/656	4.88E-02
Integrin binding	24/656	1.96E-06

KEGG

ID	Description	GeneRatio	p.adjust
hsa05200	Pathways in cancer	48/329	6.40E-06
hsa04510	Focal adhesion	32/329	7.54E-09
hsa04060	Cytokine-cytokine receptor interaction	30/329	8.47E-05
hsa04062	Chemokine signaling pathway	29/329	1.07E-07
hsa04151	PI3K-Akt signaling pathway	28/329	4.90E-03
hsa05205	Proteoglycans in cancer	27/329	3.99E-06
hsa04010	MAPK signaling pathway	25/329	3.72E-03
hsa04810	Regulation of actin cytoskeleton	23/329	3.00E-04
hsa04915	Estrogen signaling pathway	22/329	3.18E-06
hsa04514	Cell adhesion molecules (CAMs)	22/329	5.80E-06

References:

1. Xia L, Huang W, Bellani M, Seidman MM, Wu K, Fan D, et al. CHD4 Has Oncogenic Functions in Initiating and Maintaining Epigenetic Suppression of Multiple Tumor Suppressor Genes. Cancer Cell. 2017; 31: 653-68 e7.
2. Feng W, Huang W, Chen J, Qiao C, Liu D, Ji X, et al. CXCL12-mediated HOXB5 overexpression facilitates Colorectal Cancer metastasis through transactivating CXCR4 and ITGB3. Theranostics. 2021; 11: 2612-33.
3. Smolinski MP, Bu Y, Clements J, Gelman IH, Hegab T, Cutler DL, et al. Discovery of Novel Dual Mechanism of Action Src Signaling and Tubulin Polymerization Inhibitors (KX2-391 and KX2-361). J Med Chem. 2018; 61: 4704-19.
